# Serum biomarker-based early detection of pancreatic ductal adenocarcinomas with ensemble learning

**DOI:** 10.1038/s43856-023-00237-5

**Published:** 2023-01-20

**Authors:** Nuno R. Nené, Alexander Ney, Tatiana Nazarenko, Oleg Blyuss, Harvey E. Johnston, Harry J. Whitwell, Eva Sedlak, Aleksandra Gentry-Maharaj, Sophia Apostolidou, Eithne Costello, William Greenhalf, Ian Jacobs, Usha Menon, Justin Hsuan, Stephen P. Pereira, Alexey Zaikin, John F. Timms

**Affiliations:** 1 Department of Women’s Cancer, EGA Institute for Women’s Health, University College London, 84-86 Chenies Mews, London, WC1E 6HU UK; 2 Institute for Women’s Health, University College London, Cruciform Building 1.1, Gower Street, London, WC1E 6BT UK; 3 Institute for Liver and Digestive Health, University College London, Upper 3rd Floor, Royal Free Campus, Rowland Hill Street, London, NW3 2PF UK; 4 Department of Mathematics, University College London, London, WC1H 0AY UK; 5 Wolfson Institute of Population Health, Queen Mary University of London, Charterhouse Square, EC1M 6BQ, London, UK; 6 Babraham Institute, Babraham Research Campus, Cambridge, CB22 3AT UK; 7 National Phenome Centre and Imperial Clinical Phenotyping Centre, Department of Metabolism, Digestion and Reproduction, IRDB Building, Imperial College London, Hammersmith Campus, London, W12 0NN UK; 8 Section of Bioanalytical Chemistry, Division of Systems Medicine, Department of Metabolism, Digestion and Reproduction, Imperial College London, South Kensington Campus, London, SW7 2AZ UK; 9 MRC Clinical Trials Unit at UCL, Institute of Clinical Trials and Methodology, UCL, 90 High Holborn, 2nd Floor, London, WC1V 6LJ UK; 10 Department of Molecular and Clinical Cancer Medicine, University of Liverpool, Liverpool, UK; 11 Liverpool Experimental Cancer Medicine Centre, University of Liverpool, Liverpool, L69 3GL UK; 12 University of New South Wales, Sydney, NSW 2052 Australia

**Keywords:** Cancer, Computational biology and bioinformatics, Biomarkers

## Abstract

**Background:**

Earlier detection of pancreatic ductal adenocarcinoma (PDAC) is key to improving patient outcomes, as it is mostly detected at advanced stages which are associated with poor survival. Developing non-invasive blood tests for early detection would be an important breakthrough.

**Methods:**

The primary objective of the work presented here is to use a dataset that is prospectively collected, to quantify a set of cancer-associated proteins and construct multi-marker models with the capacity to predict PDAC years before diagnosis. The data used is part of a nested case-control study within the UK Collaborative Trial of Ovarian Cancer Screening and is comprised of 218 samples, collected from a total of 143 post-menopausal women who were diagnosed with pancreatic cancer within 70 months after sample collection, and 249 matched non-cancer controls. We develop a stacked ensemble modelling technique to achieve robustness in predictions and, therefore, improve performance in newly collected datasets.

**Results:**

Here we show that with ensemble learning we can predict PDAC status with an AUC of 0.91 (95% CI 0.75–1.0), sensitivity of 92% (95% CI 0.54–1.0) at 90% specificity, up to 1 year prior to diagnosis, and at an AUC of 0.85 (95% CI 0.74–0.93) up to 2 years prior to diagnosis (sensitivity of 61%, 95% CI 0.17–0.83, at 90% specificity).

**Conclusions:**

The ensemble modelling strategy explored here outperforms considerably biomarker combinations cited in the literature. Further developments in the selection of classifiers balancing performance and heterogeneity should further enhance the predictive capacity of the method.

## Plain language summary

Pancreatic cancers are most frequently detected at an advanced stage. This limits treatment options and contributes to the dismal survival rates currently recorded. The development of new tests that could improve detection of early-stage disease is fundamental to improve outcomes. Here, we use advanced data analysis techniques to devise an early detection test for pancreatic cancer. We use data on markers in the blood from people enrolled on a screening trial. Our test correctly identifies as positive for pancreatic cancer 91% of the time up to 1 year prior to diagnosis, and 78% of the time up to 2 years prior to diagnosis. These results surpass previously reported tests and should encourage further evaluation of the test in different populations, to see whether it should be adopted in the clinic.

## Abstract (web summary)

Nené et al. construct machine learning models based on serum protein biomarker data to detect pancreatic ductal adenocarcinoma in a nested case-control study from the UKCTOCS cohort. Their ensemble modelling approach outperforms existing combinations of biomarkers.

## Introduction

Pancreatic ductal adenocarcinoma (PDAC) is associated with dismal 5-year survival rates (~3–7%) and is projected to become the second cause of cancer deaths by 2030^1–3^. A non-specific clinical course leading to late-stage diagnosis is a feature of pancreatic cancer, and only 15% of the patients are diagnosed at early stages with resectable tumours^2–5^. Following surgery and adjuvant therapy however, less than 30% of patients survive 5 years after diagnosis^5^, compared with a <10% 5-year survival in those with unresectable disease^6^. The development of new tests that could improve detection of early-stage disease is pivotal for optimal outcomes for pancreatic cancer patients. Indeed, it has been shown that if tumour size at detection can be reduced from 3 to 2 cm, improved oncological resection rates (7% to 83%, respectively) and increased median survival (from 7.6 to 17.2 months) can be achieved^7,8^. CA19-9^9,10^ is the only serological tumour marker used routinely for confirmation of diagnosis and monitoring of PDAC progression, however, with 79–81% test sensitivity and 82–90% specificity at best^11^. Despite this, we have recently shown that CA19-9 (and CA125) can be used to detect pancreatic cancer up to 2 years before clinical diagnosis, using samples from a repository collected as part of the UK Collaborative Trial of Ovarian Cancer Screening (UKCTOCS)^12^. These samples, which were prospectively collected months and years prior to diagnosis, enabled the detection of rising levels of potential serological biomarkers ahead of PDAC diagnosis with high reliability. We proposed that CA19-9 in combination with markers identified in this cohort and their use in multi-marker algorithms, may improve performances and enable early detection of PDAC.

In a rapidly evolving omics era, multi-cancer early detection tests (MCETs) are increasingly reported. Two outstanding MCETs include the CancerSEEK^13^ and the Galleri (GRAIL) tests^14,15^. These multi-analyte tests analyse circulating tumour (cell free) DNA for specific genetic mutations in combination with proteins (CancerSEEK) or cancer-associated methylation patterns (Galleri). With a median sensitivity of 67% (at ~99% specificity) for 12 cancers, CancerSEEK test sensitivity for detecting pancreatic cancer (stages I–III) was reported as 72% (99% specificity)^13^. With variable sensitivities across 12 cancer types (11.2% for prostate to 93.5% for liver/biliary tract cancers), the reported overall sensitivity (at 99.5% specificity) for PDAC detection was 83.7%. For early PDAC (stage I–II) test sensitivity was around 60%. The performance of such tests in larger cohorts in which the low prevalence of PDAC is more accurately reflected, however, requires further validation prior to their implementation as screening tools.

Combining markers into multi-marker models has traditionally involved the application of simple cut-off rules and machine learning methods such as multivariate logistic regression^12^, neural networks and support vector machines^16–18^. Here, to attain better performances and robustness, we applied an ensemble modelling technique^19–22^ and a repeated cross-validation resampling strategy. Ensemble methods have been immensely successful in producing accurate predictions for many complex classification tasks^19,21,23^, because they address fundamental problems in data analysis. For example, these methods avoid overfitting by combining single learners with a local search heuristic which decreases the risk of obtaining a local performance minimum. Issues surrounding dimensionality are also addressed with ensemble models. By allowing each classifier to focus on sub-spaces of features, the burden of large search spaces is reduced. This specific field in machine learning is consistent with the well-known Condorcet’s jury theorem, which states that if each classifier has a probability larger than 0.5 of being correct then increasing the pool of classifiers increases the probability of making the correct decision by majority voting. The task of finding successful ensembles is, nevertheless, more complex, and dependent on a balance between diversity and consensus among classifiers. A definitive recipe to achieve this goal has yet to be completely defined^19,21,23^. Two widely cited examples following the ensemble paradigm that focus on complementary and heterogeneity are, for instance, stacking, a form of meta-learning^21^, and ensemble selection^19^. Stacking constructs a higher-level predictive model over the predictions of base classifiers. Ensemble selection, on the other hand, uses an incremental strategy to select base predictors for the ensemble while balancing diversity and performance^19^.

Here we apply the stacking approach due to its simplicity and computational efficiency. The use of multi-datasets and multi-platform integration in pancreatic cancer studies^24^ are essential for early detection and aligns at a fundamental level with the data analysis methodology applied in our work. We demonstrate how using a stacked ensemble approach which relies on a panel of 20 features, including cancer-associated proteins and clinical covariates, outperforms state-of-the-art multi-biomarker combinations previously applied in pancreatic ductal adenocarcinoma early detection.

## Methods

### Study design

This nested case control discovery study was approved by the Joint UCL/UCLH Research Ethics Committee A (Ref. 05/Q0505/57). Written informed consent for the use of samples in the UKCTOCS trial and secondary ethically approved studies was obtained from donors and no data allowing identification of patients was provided. The study set comprised serum from post-menopausal women aged 50–74 recruited to UKCTOCS between 2001 and 2005 and collected according to an SOP^25,26^. All participants were ‘flagged’ with the national agencies for cancer registrations and deaths using their NHS number. Women subsequently diagnosed with pancreatic ductal adenocarcinoma (cases) were identified by cross-referencing with the Health and Social Care Information Centre cancer registry codes and death codes (ICD10 C25.0/1/2/3/9). Confirmation of diagnosis was sought from GPs and consultants through questionnaires and from the Hospitals Episode Statistics database. In total, 143 cases were identified (with 218 associated serum samples) that had not been registered as having any other cancer since randomization and that had a confirmed diagnosis of pancreatic cancer. Matched non-cancer controls, i.e., with no cancer registry code, from individual women were selected based on collection date, age, and centre to minimize variation due to handling and storage. From this set, 249 controls were selected. 35 of the PDAC cases had longitudinal data, with between 2 and 6 annual longitudinal samples per individual years before diagnosis (Table 1). Due to the design of the UKCTOCS study, PDAC stage for all the cases at the time of diagnosis was not available. The number of cases and controls selected from UKCTOCS for the study presented here as well as other characteristics such as body-mass index (BMI) and Age at diagnosis can be seen in Table 1. Detailed diabetes information for the UKCTOCS participants selected for this study was not available or was incomplete. Disease duration was also unavailable. Any stratification based on diabetes type was therefore not done.

**Table 1:** Study dataset description.

Variable	Cases	Controls	*P* value
No. samples	218	249	
Tumour site			
Tail	10	na	
Body	22	na	
Body/tail	3		
Head	88	na	
Unspecified	95	na	
Mean time to spin (h) (range)	21.7 (0.48–46.53)	21.82 (0.32–46.24)	0.93
Mean age at sample draw (yr) (range)	64.94 (51.19–74.87)	62.48 (50.44–76.86)	<0.0001 (OR = 1.06)
Mean BMI (kg/m^2^) (range)	27.46 (17.80–43.74)	26.64 (17.91–44.39)	0.078
Mean time from sample collection to diagnosis (months) (range)	26.07 (0.99–70.09)	na	
HRT use (at randomisation)			
Yes	19	48	0.0010
No	199	201	(OR = 0.41)
OCP use (ever)			
Yes	132	127	0.039
No	86	122	(OR = 1.47)
Diabetes			
Yes	42	11	<0.0001
No	176	238	(OR = 4.99)

*P* values were calculated according to a logistic regression model with a bias reduction method (see ‘Methods’), for the whole UKCTOCS sample distribution. For the same variables in the subsets used as training and test sets see Supplementary Tables 1 and 2. This table corresponds to taking all samples from all time-groups, i.e., 0 to 4+ years to diagnosis (*n* = 467). See also Supplementary Figs. 1, 2.

*BMI* body mass index, *OCP* oral contraceptive pill use, *HRT* hormone replacement therapy.

Diabetes status was collected from a UKCTOCS first follow-up questionnaire, from in-patient and out-patient Hospital Episode Statistics or from death certificates. The full dataset used in our work included only 44 individuals for which type was determined. For the rest, only yes/no information was available with respect to diabetes. 24 PDAC cases with diabetes mellitus (DM) were non-insulin-dependent, 3 were classified as insulin-dependent, 3 had both a classification of insulin-dependent and non-insulin-dependent DM, therefore inconclusive, 1 had a non-insulin-dependent and other specified DM, 1 had a non-insulin-dependent and unspecified DM, 5 had an only unspecified DM classification. Regarding the controls, only 1 had insulin-dependent DM and 5 were classified as non-insulin-dependent. Due to the smaller size of the subset for which DM type was available and the incomplete nature of this information, we decided to not incorporate type into our analysis.

As an external validation cohort, we resorted to the Accelerated Diagnosis of neuro Endocrine and Pancreatic TumourS (ADEPTS) study^27^ (UCL/UCLH Research Ethics Committee reference 06/Q0512/106), which is an early biomarker study aiming to detect pancreatic cancer in patients at a much earlier stage. The ADEPTS study, previously referred to as TRANSlational research in BILiary tract and pancreatic diseases (TRANSBIL) study, collected serum samples from adult patients who presented to University College London and the Royal Free London Hospitals between 2017 and 2019 with abdominal symptoms suggestive of hepatobiliary disorders and pancreatic cancer. For the purpose of this work, samples from patients with no underlying gastrointestinal disorders or samples from cases diagnosed with pancreatic cancer were used. The number of cases and controls selected for external validation of the PDAC signature developed in the UKCTOCS samples presented above can be seen in Table 2 (see also Supplementary Table 3), as well as other sample characteristics. 17 PDAC cases and 17 controls were available for the work presented here. The controls from the ADEPTS study are the closest to the control population collected from UKCTOCS as they did not present underlying gastrointestinal pathology. The PDAC cases used here had been matched by age, gender and diabetes status. Hormone replacement therapy (HRT) use at randomization and oral contraceptive pill (OCP) use (ever) information was not collected for the female participants. All patients have given written consent for the use of their samples for research purposes and data were anonymized. The samples were processed according to NIHR standards^28^ and diagnoses were confirmed by interrogating patient hospital electronic records at University College London and the Royal Free Hospitals.

**Table 2:** External validation set characteristics.

Variable	Cases	Controls	*P* value
No. samples	17	17	
Tumour site			
Tail	Not collected	na	
Body	Not collected	na	
Body/tail	Not collected		
Head	Not collected	na	
Unspecified	Not collected	na	
Mean time to spin (h) (range)	(0.5–1.5)	(0.5–1.5)	–
Mean age at sample draw (yr) (range)	71.94 (43.00–88.00)	54.47 (27.00–89.00)	0.0054 (OR = 1.08)
Mean BMI (kg/m^2^) (range)	24.66 (12.04–41.35)	23.84 (18.02–29.20)	0.58
Gender			
Male	9	5	0.17
Female	8	12	
Diabetes			
Yes	1 (Type II)	4 (3 Type II, 1 unspecified)	0.18
No	16	13	

*P* values were calculated according to a logistic regression model with a bias reduction method (see ‘Methods’). Samples were collected from the ADEPTS cohort (see ‘Methods’, *n* = 34). See also information on pancreatic ductal adenocarcinoma (PDAC) stage in Supplementary Table 3.

*BMI* body mass index.

### Serum analyte measurements

**Fig. 1:**
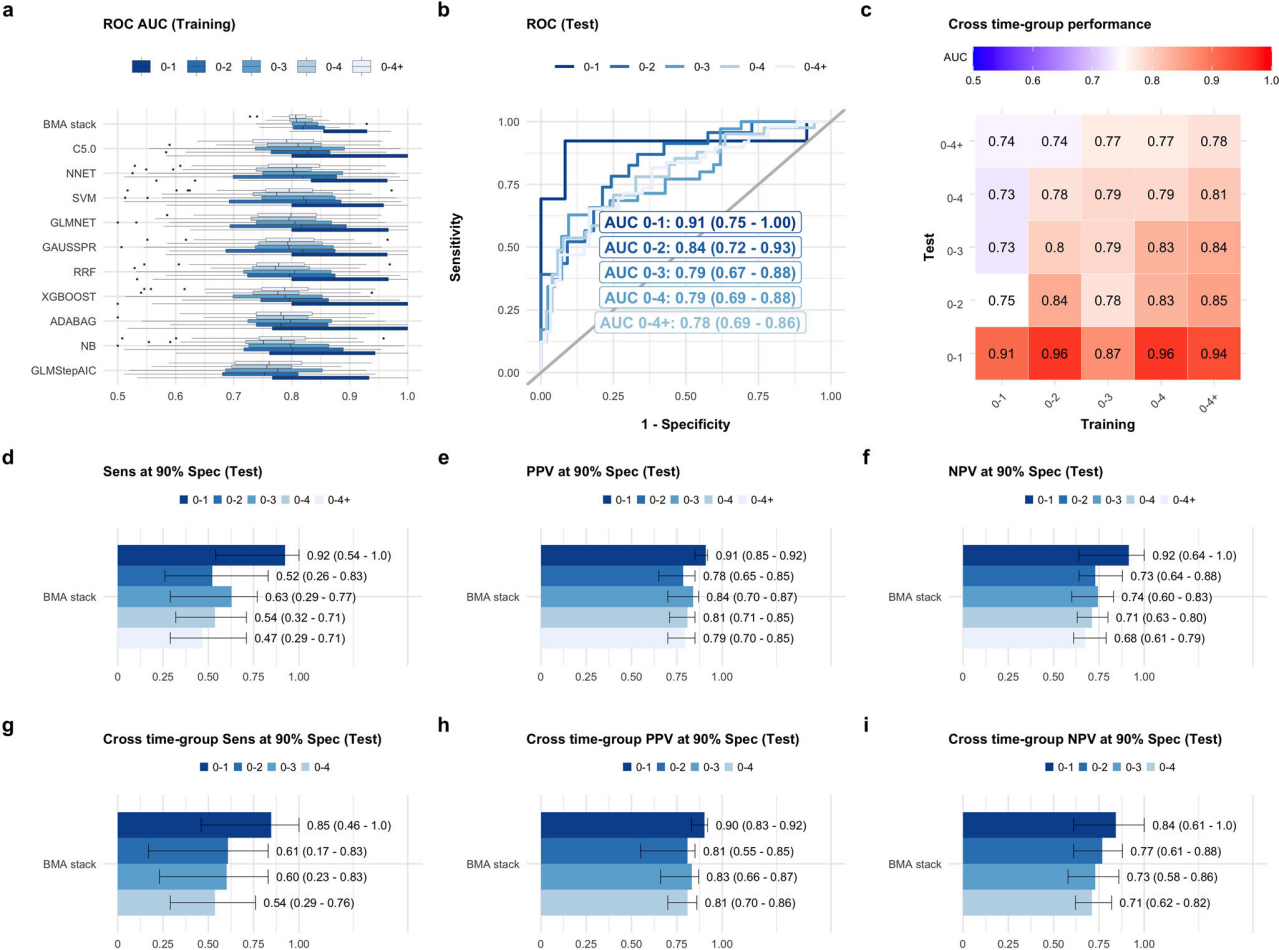
Ensemble model performance per joined/combined time-group. **a** Distribution of receiver operating curve (ROC) area under the curve (AUC) across training folds for each of the base-learners and the Bayesian Model Averaging (BMA) stack meta-learner (Joined Time Group 2 Layer (JTG2L) model, see ‘Methods’ section on statistical analysis). See also Supplementary Figs. 24, 25 to 28 for alternative stacking methods. **b** ROC curves in the test set for the BMA stack per joined time-group. AUC 95% Confidence Intervals (CI) were determined by stratified bootstrapping. **c** Cross-time group performance of the BMA stack developed in the training set and evaluated in specific time-groups in the test set. 95% CI for AUCs are not shown but the predictions were all significant. **d** Sensitivity (Sens), **e** Positive predictive value (PPV) and **f** Negative predictive value (NPV) at 90% Specificity (Spec) corresponding to **b**. **g**–**i** Cross time-group performances for the ensemble trained in 0-4+ samples (last column in **c**). See also Supplementary Fig. 29 for other stacking methods. For the Matthew correlation coefficients corresponding to **d**–**i**, see Supplementary Fig. 30. In **a**, **b**, **d**–**i**, shades of blue from dark to light correspond to results obtained in 0-1, 0-2, 0-3, 0-4 and 0-4+ years to diagnosis samples, respectively. The number of independent training samples was *n* = 107 (0-1), *n* = 180 (0-2), *n* = 252 (0-3), *n* = 309 (0-4) and *n* = 363 (0-4+). The number of independent test set samples was *n* = 26 (0-1), *n* = 60 (0-2), *n* = 82 (0-3), *n* = 98 (0-4) and *n* = 114 (0-4+). See Supplementary Table 12 for further details on case and control samples. See ‘Statistical analysis’ in Methods for further details and Supplementary Data 1–3.

**Fig. 2:**
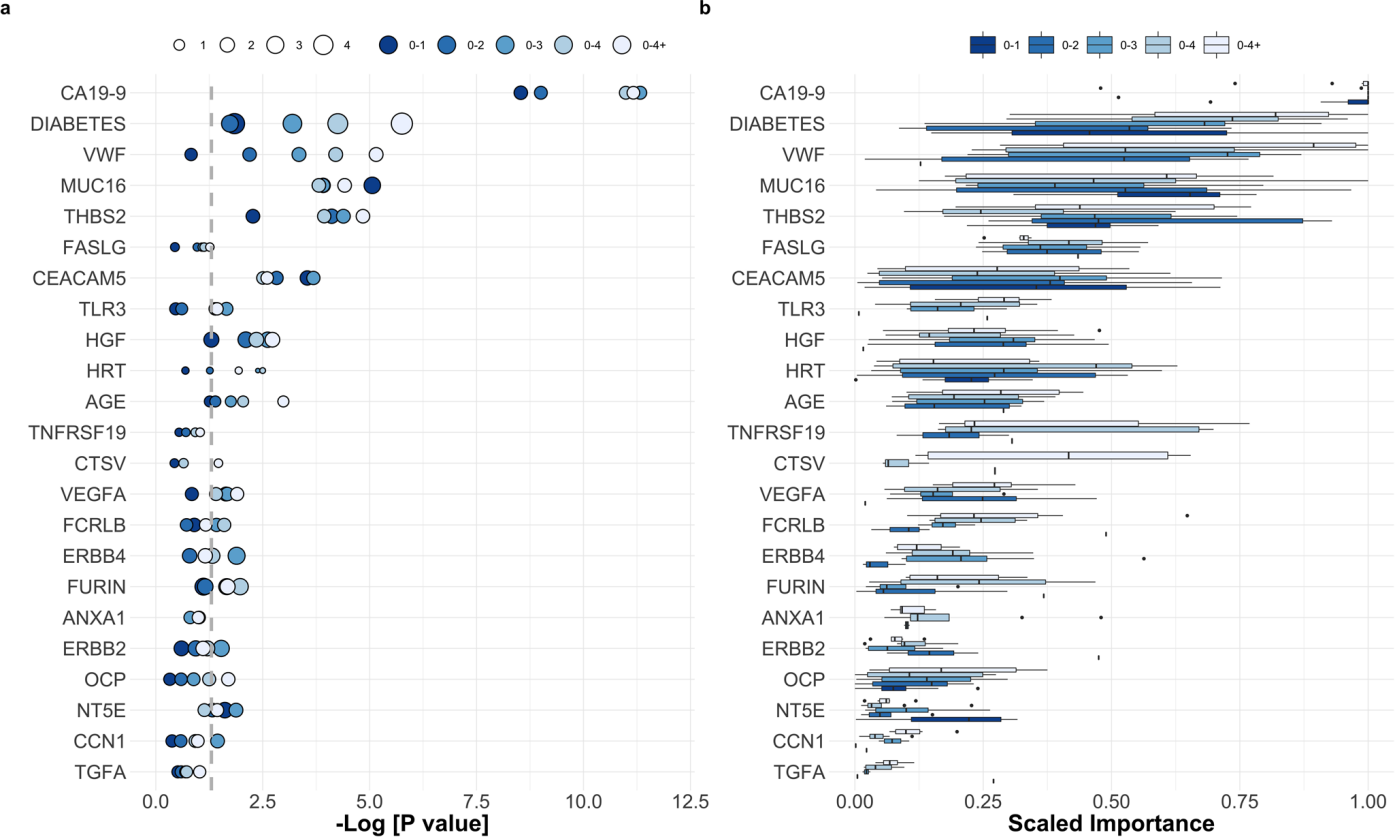
Feature importance across pancreatic ductal adenocarcinoma base-learner signatures. **a** Odds-ratios (represented proportionally by the size of the circles) and *P*-values for the ranking procedure according to a logistic regression model using Firth’s bias reduction method in the training set. **b** Feature importance across all base learners and joined time-groups. All the features (biomarkers and clinical covariates) presented in this figure were selected when training/optimizing the ensemble approach with 0-4+ samples. The importance plotted for the remaining joined time-groups is the importance of each feature in their respective models. See also Supplementary Fig. 33 for the full plots and additionally Supplementary Fig. 34 for models developed with single time-groups. In **a** and **b** shades of blue from dark to light correspond to results obtained in 0-1, 0-2, 0-3, 0-4 and 0-4+ years to diagnosis samples, respectively. See ‘Statistical analysis’ in Methods for further details and Supplementary Data 4, 5. OCP oral contraceptive pill use. HRT hormone replacement therapy.

**Fig. 3:**
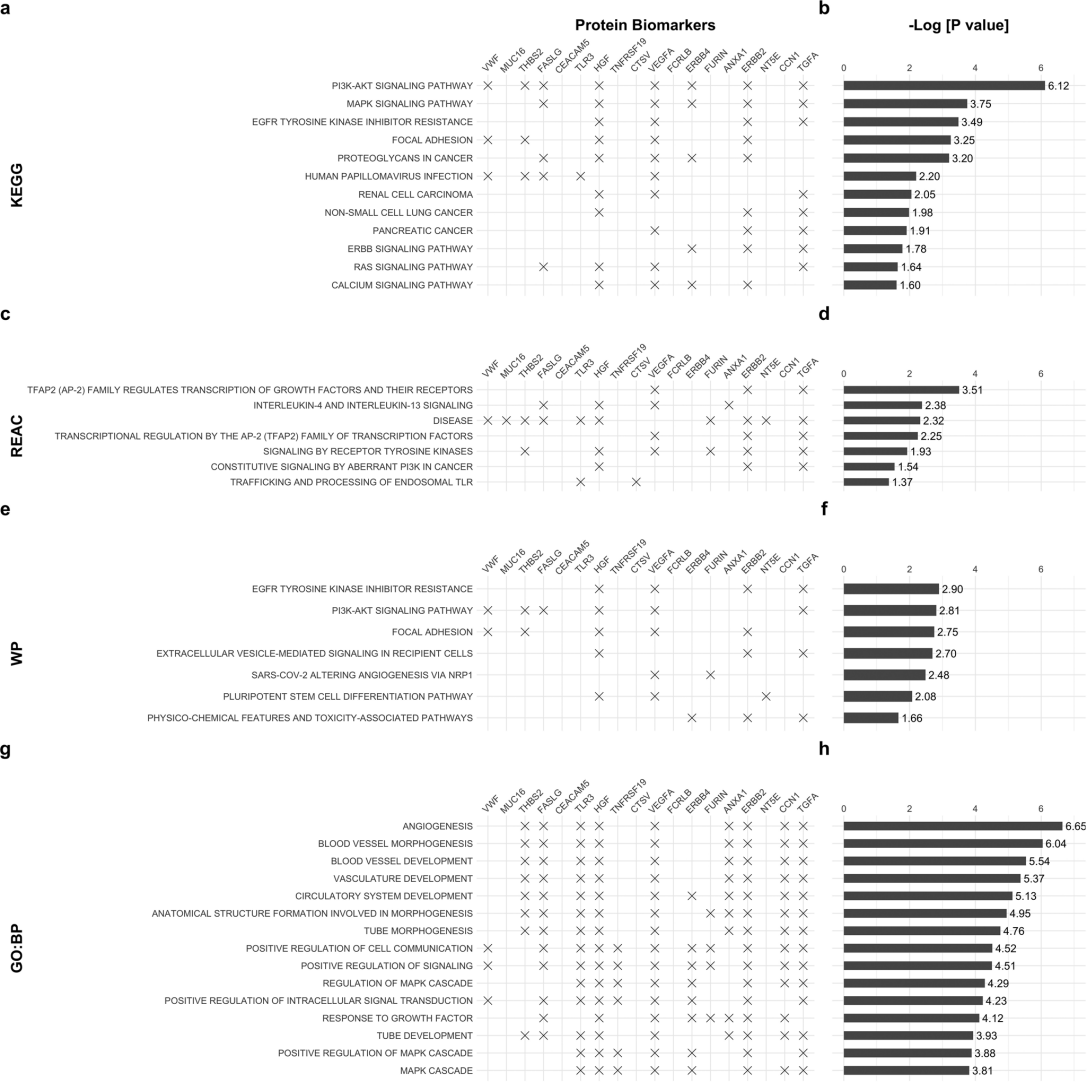
Enrichment analysis. g:Profiler terms for the set of features selected by the optimal classifier trained in 0-4+ samples. **a** Kyoto Encyclopaedia of Genes and Genomes (KEGG) pathways. **c** Reactome Pathway Database (REAC). **e** WikiPathways (WP). **g** Gene ontology terms biological process (GO: BP). The respective adjusted *p*-values associated with each enrichment term or pathway are plotted in (**b**), (**d**), (**f**) and (**h**). See also Fig. 2. See ‘Statistical analysis’ in Methods for further details and Supplementary Data 6.

**Fig. 4:**
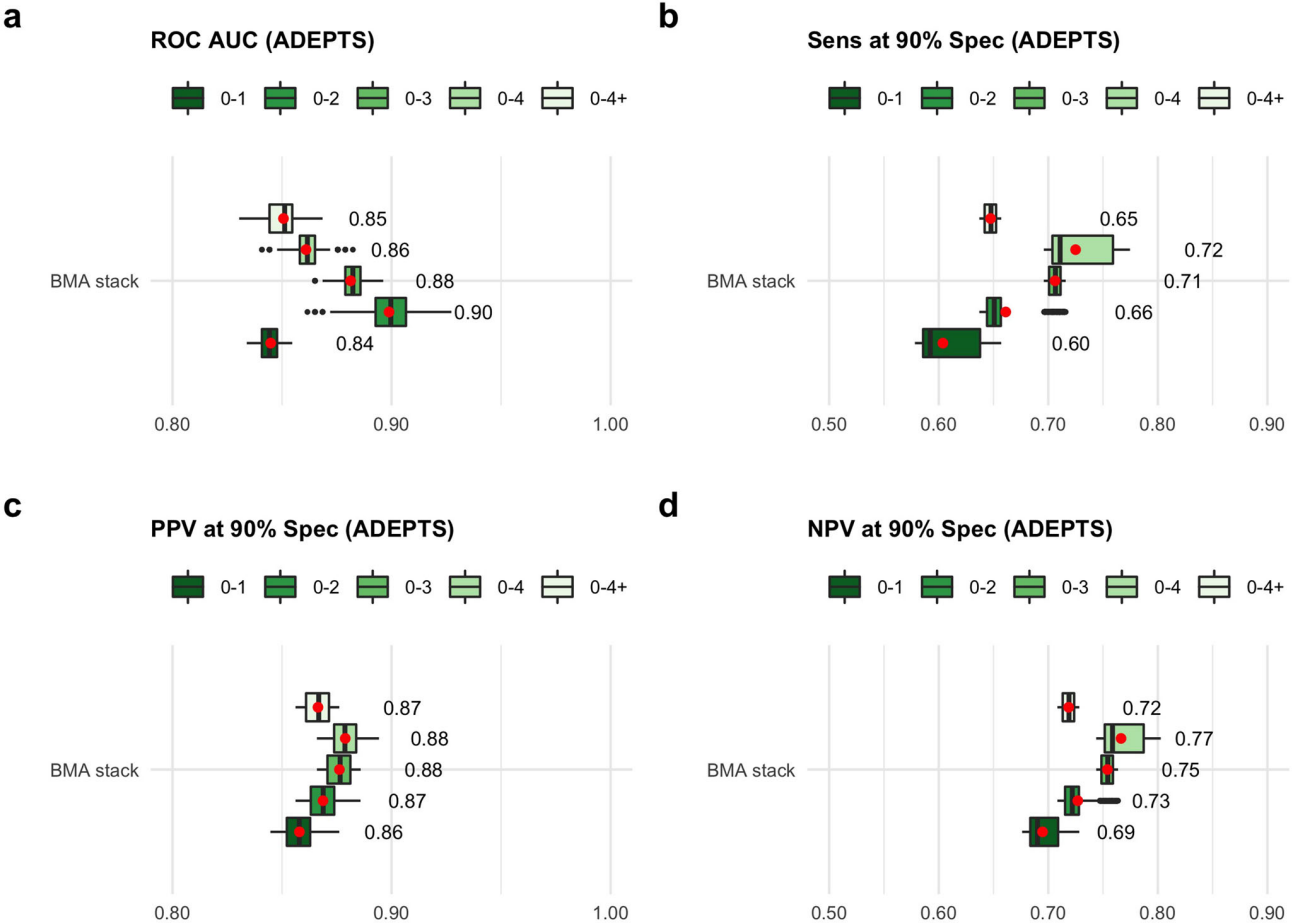
Performance in an external validation set. **a** Receiver operating curve (ROC) area under the curve (AUC) in the Accelerated Diagnosis of neuro Endocrine and Pancreatic TumourS (ADEPTS) external validation set for the Joined Time Group 2 Layer (JTG2L) Bayesian Model Averaging (BMA) stack models developed and selected in the UKCTOCS training set in the respective joined time-group samples (see Fig. 1), coloured in shades of green from dark to light for 0-1, 0-2, 0-3, 0-4, 0-4+ YTD samples. **b** Sensitivity (Sens), **c** Positive predictive value (PPV) and **d** Negative predictive value (NPV) at 90% specificity (Spec) (see also Supplementary Fig. 39 for the corresponding Matthew’s correlation coefficient value). The performances correspond to 1000 datasets whose difference from the original ADEPTS subset selected for this study is the random allocation of the missing features hormone replacement therapy (HRT) and oral contraceptive pill use (OCP) to female participants. The red dots and respective numbers correspond to estimates of the mean performance in ADEPTS (by bootstrapping with the *boot* R package (version 1.3–25)) for the respective model developed in UKCTOCS time-grouped samples. The number of independent ADEPTS samples was *n* = 34. See ‘Study design’ and ‘Statistical analysis’ sections in Methods for further details, and Supplementary Data 7.

All UKCTOCS serum samples were randomized for testing. Supplementary Table 4 summarizes dilution factors and coefficients of variation. Carbohydrate antigen 19-9 (CA19-9(A)) was measured using the Mucin PC/CA19-9 ELISA Kit (Alpha Diagnostic International) according to the manufacturer, using a 1:4 serum dilution. CA125(A), Mucin-16 (MUC16) assay was performed using the Cobas CA125 II CLIA with a CA125 II Calibrator Set (Roche and Fujirebio Diagnostics) on a Cobas E411 analyzer with PreciControl Tumour Marker to monitor assay imprecision. Leucine-rich alpha-2-glycoprotein (LRG1) level was assessed using the human LRG1 ELISA Assay Kit (Immuno-Biological Laboratories) at a 1:2000 serum dilution. Polymeric immunoglobulin receptor (PIGR) was measured using the human secretory component (SC) ELISA Kit (Cusabio) at a 1:500 serum dilution. Regenerating family member 3 alpha (REG3A/PAP) level was determined using the PANCREPAP ELISA Kit (DynaBio) at a 1:100 serum dilution and Factor XII (F12) using the Factor XII Human ELISA Kit (Abcam) at a 1:1000 serum dilution. For the von Willebrand factor (VWF), we resorted to the Von Willebrand Factor Human ELISA Kit (Abcam) at a 1:100 serum dilution. Thrombospondin-1 (THBS1/TSP1) level was evaluated using the Quantikine Human Thrombospondin-1 Immunoassay (R&D Systems) at a 1:100 serum dilution. Anterior gradient protein 2 homolog (AGR2) was calculated using the Anterior Gradient Protein 2 ELISA kit (USCN Life Science) at a 1:25 serum dilution. Alpha-1 antitrypsin (A1AT/SERPINA1) was measured by α-1-Antitrypsin ELISA kit (Immunodiagnostik AG) and Interleukin 6 signal transducer (IL6ST/IL6RB) by Quantikine human soluble gp130 (R&D Systems), according to manufacturer recommendations. Thrombospondin-2 (THBS2/TSP2) was measured using the Quantikine Human Thrombospondin-2 Immunoassay (R&D Systems) at a 1:10 serum dilution, TEK Receptor Tyrosine Kinase (TEK) using the Quantikine human TIE-2 ELISA Assay Kit (R&D Systems) at a 1:10 serum dilution and Insulin-like growth factor binding protein 1 (IGFBP1) by human IGFBP1 ELISA Assay Kit (Abcam) at a 1:50 serum dilution. Finally, Interleukin 17 receptor A (IL17RA) was measured using the human IL17RA ELISA Assay Kit (Abnova) and Alpha Fetoprotein (AFP) by using the Human AFP Quantikine Immunoassay, according to manufacturer recommendations. Assays were performed on singlet test samples and values not measurable on the standard curves were given the value ‘low’. For the main results shown in Figs. 1–4, from the markers listed above only CA19-9, CA125/MUC16, VWF, THBS2 and IL6ST were used. See Supplementary Discussion, Supplementary Figs. 3, 9 and Supplementary Tables 5–10 for further details and results on the use of a portion of the markers listed above but for a much smaller set of participants. All samples were also tested using Olink’s multiplex immunoassay Oncology II panel. Known cancer antigens, growth factors, receptors, angiogenic factors and adhesion regulators were measured (see Supplementary Table 11).

As with the UKCTOCS samples, the same assays were run in the subset of samples collected from the ADEPTS study, as well as the same Olink platform of biomarkers. This secured that the full biomarker signature developed in UKCTOCS samples could be validated in a different cohort.

### Statistical analysis

Our main dataset is part of a nested case-control study within UKCTOCS^25,26^ and is comprised of 143 individuals with PDAC and 249 controls (see Table 1 and Supplementary Tables 1 and 2). Thirty-five of the PDAC-diagnosed patients provided longitudinal samples, ranging between 2 and 6 annual samples per individual collected prior to diagnosis, with an average of 1.53 samples per individual (see Table 1). Despite the fact that 35 of the PDAC cases had longitudinal data, all samples were taken as independent, and no intra-individual correlation was imposed or explicitly modelled during data analysis in this instance. For the purpose of data analysis, we divided all samples prior to any classifier development into a training (2/3) set and test (1/3) set, by stratifying for age quartile, HRT use at randomization, OCP use (ever), diabetes status (Yes/No), BMI quartile, PDAC or control status and for sample single time-group, i.e., 0-1,1-2,2-3,3-4 and 4+ years to diagnosis (YTD). Sample single time groups were attributed to each sample and determined by the time to diagnosis at sample collection (compare Table 1 with Supplementary Tables 1 and 2, see also Supplementary Table 12 for the total number of cases and controls per single time-group). The stratification outlined above enabled a clearer evaluation of PDAC classifier panel performances in collected samples not used in training, i.e., the test set, and ensured that the results are realistic and representative, and are not biased by data or information leakage^29^.

Receiver operating characteristic (ROC) curves were constructed for each model to assess diagnostic accuracy. The area under the curve (AUC) for the ROC curves was used as the performance metric during optimization. Models were selected based on their rank in the training set across cross-validation folds. ROC curves were generated with the *pROC* R package (version 1.18.0, https://cran.r-project.org/web/packages/pROC/index.html). 95% CI for AUCs were determined by stratified bootstrapping. All AUC confidence intervals crossing 0.5 were deemed insignificant. In addition, sensitivity, positive and negative predictive values and Matthews correlation coefficients at 90% specificity are also reported. Comparison of ROC curves was performed with a bootstrap test in *pROC*.

In order to evaluate the association between each of the single markers, including the clinical covariates (see Table 1), and PDAC status, we resorted to the logistic regression model implemented in the *logistf* R package (https://cran.r-project.org/web/packages/logistf/index.html, version 1.24.1). This approach fits a logistic regression model using Firth’s bias reduction method. The reported confidence intervals for odds ratios and tests were based on the profile penalized log likelihood and incorporate the ability to perform tests where contingency tables are asymmetric or contain zeros. *P* values were used to rank markers. The performance of single marker models was also verified in the test set (see Supplementary Figs. 10, 24 and Supplementary Discussion for further details and results).

Multi-dimensional analysis of the data was performed under two separate frameworks: a brute-force algorithm scanning through combinations of up to 3 markers and fitting a logistic regression model (see Supplementary Discussion), and a stacked ensemble algorithm with 10 base-learners. The models stemming from the brute-force approach were ranked according to their performance across cross-validation training folds (Supplementary Figs. 21, 22). The ensemble models relied on the performance of the base-learners presented in Fig. 1 (see also Supplementary Fig. 23) and highlighted below, run through caret (version 6.0-93, https://cran.r-project.org/web/packages/caret/index.html): decision trees and rule-based models for pattern recognition (C50, version 0.1.6, https://cran.r-project.org/web/packages/C50/index.html); support vector machines with radial basis function kernel (SVM, version 0.9-31, https://cran.r-project.org/web/packages/kernlab/index.html); regularized random forests (RRF, version 1.9.4, https://cran.r-project.org/web/packages/RRF/index.html); neural networks with feature extraction (NNET, version 7.3-17, https://cran.r-project.org/web/packages/nnet/index.html); gaussian process with radial basis function kernel (GAUSSPR, version 0.9-31, https://cran.r-project.org/web/packages/kernlab/index.html); lasso and elastic-net regularized generalized linear models (GLMNET, version 4.1-4, https://cran.r-project.org/web/packages/glmnet/index.html); bagged Adaptive Boosting (ADABAG, version 4.2, https://cran.r-project.org/web/packages/adabag/index.html); extreme gradient boosting (XGBOOST, version 1.6.0.1, https://cran.r-project.org/web/packages/xgboost/index.html); generalized Linear Model with Stepwise Feature Selection with Akaike Information criterion (GLMStepAIC, version 7.3-58, https://cran.r-project.org/web/packages/MASS/index.html); naïve Bayes classifier (NB, version 1.7-1, https://cran.r-project.org/web/packages/klaR/index.html).

The selection of base-learners was grounded on covering a number of state-of-art methods and algorithmic families, from bagging and boosting to general linear models with in-built feature extraction, previously referenced in the literature^19,22^, that would be able to capture different aspects of the data with an efficient computational effort and that had, for the most part, typical hyperparameter ranges published in the literature^22^, some with applications in biology^21^. Due to the size of the datasets, we narrowed down the size of the set of base-learners to 10. Further work on ensemble selection from libraries of models should contribute to clarifying if other techniques provide additional value^19^ by testing performance against base-learner pool diversity^21^. The training of the base-learners was executed in two ways: by taking joined/combined time-group samples, i.e., collected 0-1, 0-2, 0-3, 0-4, 0-4+ YTD or by training the set of base-learners in each single time-group specific samples, i.e., 0-1, 1-2, 2-3, 3-4, 4+ YTD. The first model forces the base-learners to learn specific and cross-time-group details together, whereas the second model creates specialized groups of base-learners per single time-group. We tested several staking procedures, i.e. the meta-learners: by Bayesian Model Averaging (BMA) (version 3.18.17, https://cran.r-project.org/web/packages/BMA/index.html) with an underlying logistic regression model (BMA stack), by averaging with an arithmetic mean (MEAN stack) and geometric mean across the probabilities attributed by each base-learner (GEOMEAN stack), or by taking the maximum probability of being a case across all base-learners (MAX stack). This class is named throughout this paper as Joined Time Group 2 Layer (JTG2L) (see Fig. 1 and Supplementary Figs. 24 and Supplementary Table 13 for the optimal hyperparameters found through a random selection of 1000 combinations for each base-learner). For the second model we tested a 2-layer and a 3-layer stacked model. The first, referred to as Single Time Group 2 Layer (STG2L) (Supplementary Fig. 25), took the base learners trained in each single time-group and applied the 4 stacks mentioned above, although to a larger stack input space. If, for example, we are training with samples belonging to every single time-group, i.e., 0-1, 1-2, 2-3, 3-4, 4+, the stack feature input space will have 10 times 5 dimensions; each base-learner is trained on each single time-group, giving 5 models per base-learner and a total of 50 base-models (Supplementary Fig. 25). Subsequently, the probability output from each base-learner model is concatenated and fed into the meta-learner. For the specific case of the STG2L protocol, we also tested an average neural network meta-learner model (AVNNET stack) trained on the concatenated probability matrix created from each base-learner probability output. The second, named Single Time Group 3 Layer (STG3L) (Supplementary Fig. 26), stacks twice and, therefore, has 3 layers. First it stacks the base-learners per single time-group with a BMA stack and, subsequently, stacks the result, a 5-dimension feature space of probabilities with either a BMA stack, a MEAN stack, a GEOMEAN stack or a MAX stack, if, for example, we are training with samples belonging to every single time-group (see Supplementary Fig. 26 for further details). Other combinations of time-groups were also tested, e.g., 0-1 plus 1-4, 0-2 plus 2-4, etc., but the stacked classifiers either underperformed or were not robust.

All base-models were trained by 5 times repeated 10-fold cross-validation with over-sampling of the minority class, in our dataset the PDAC cases (see Table 1). In order to further avoid overfitting, we ranked each of the features, both biomarkers and clinical covariates, with the logistic regression model mentioned before, and scanned the ranked feature input space, in increments of 10 features, with the objective of finding the optimal performance across cross-validation folds, without bias^30^. Despite some features not being significant according to the logistic regression model with bias correction when evaluated as a single predictor, the protocol we applied scanned over all features, clinical covariates included. As described, the stacked models were trained on the probability matrices, i.e., generated by concatenating the vectors whose entries are the probability of being a PDAC case according to each base-learner. We opted for a 10-times 10-fold cross-validation resampling strategy for the meta-learner to further secure that the choice of the best models was robust. The extensive resampling strategy secured both at the base-learner level as well as the meta-learner level that the models learned performed robustly across a large number of diverse folds. We also developed other classifiers trained in both real and synthetic data by applying state of the art techniques such as SMOTE^31^ and non-parametric algorithms^32^ during resampling (see Supplementary Discussion and Supplementary Figs. 31, 32 for details).

The fact that the PDAC cases had longitudinal samples which we considered as independent, did not affect the training of the models. The 5-times repeated 10-fold cross-validation resampling strategy secured that the random allocation of samples to training and validation folds during training avoided a systematic use of samples from the same individual in hyperparameter optimization. Regarding the stratification of training and test sets, given that this was done also with information of sample single time-groups, there wasn’t a consistent presence of samples from the same individual which would interfere with the performances reported here.

The variable importance routine selected for evaluating feature importance in each base-learner (see for example Fig. 2) was a model-agnostic method based on a simple feature importance ranking measure^33^, implemented in the R package *vip* (version 0.3.2, https://cran.r-project.org/web/packages/vip/index.html). Model-agnostic interpretability separates interpretation from the model, is a more flexible approach and can be applied to any supervised learning algorithm. It was crucial in our case for understanding the variance in importance attributed by each base-learner.

The enrichment analysis for each of the signatures developed with single and joined time-groups was performed with the *gprofiler2* R package (version 0.2.1, https://cran.r-project.org/web/packages/gprofiler2/index.html) version 0.2.0. In Fig. 3 only up to 15 significant terms are shown. Threshold for multiple comparison correction under false discovery rate was set at 0.05.

### Reporting summary

Further information on research design is available in the Nature Portfolio Reporting Summary linked to this article.

## Results

### Dataset characteristics

Time intervals between UKCTOCS sample collection and serum isolation, i.e., time to spin, were comparable between PDAC cases and controls. There was no significant difference in the mean time to spin between cases and controls for the whole study dataset (Table 1), with the ranges being also very similar. The same is observed in the training and test sets (Supplementary Tables 1 and 2). The distribution of ages at sample draw showed a significant association (OR = 1.06, *P* < 0.0001) with PDAC status, with cases having a mean value at 64.94 years and controls at 62.48 years. Once again, a similar observation regarding significance can be made for the training and test sets. In both, we verify odds-ratios favouring PDAC status (Supplementary Tables 1 and 2). Through further analysis in the training set, we verified that age at sample draw was, nevertheless, only significantly associated with PDAC in training samples obtained 4+ years prior to diagnosis (Supplementary Figs. 1, 10). Moreover, by applying the logistic regression model used for the ranking of individual features which was developed in the training set (see ‘Methods’), we observed that age did not generate significant AUCs in the test set, and the sensitivities were very low (Supplementary Figs. 1, 10). To qualify as a predictive marker, participant age had to be combined as a covariate in a multi-marker model as is reported below (see also Supplementary Discussion for an extensive study on typical biomarker combinations in simple state-of-the-art logistic regression models). As a single variate, participant BMI was neither a significant predictor in the entire dataset (Table 1) nor was it in the training (Supplementary Table 1). It was also unable to achieve significant performances in the test set with a simple general linear model (Supplementary Table 2 and Supplementary Fig. 10c, d). Regarding HRT, it was significant in all datasets (Table 1, Supplementary Tables 1 and 2). In the training set, this result stemmed mostly from the association of HRT use with a lower risk of pancreatic cancer (OR = 0.22 < 1, 95% CI 0.04–0.85, *P* = 0.027, coloured in blue in Supplementary Fig. 10e) in the 2-3 single time-group samples. Its significant predictive potential for distinguishing between cases and controls, however, was not reproducible in the test set 2-3 time-group by applying a logistic regression model developed in the training set, AUC = 0.56 (95% CI 0.50–0.67) (Supplementary Fig. 10f). Similarly to age, HRT only added value when part of a larger PDAC predictive index (see below). Although demonstrating an association with PDAC status across the entire study set (Table 1) and the training set (Supplementary Table 1), OCP was not a significant predictor in any of the single time-group samples evaluated independently (Supplementary Fig. 10e). Overall, diabetes was the strongest predictor of risk for PDAC among the clinical covariates (Table 1 and Supplementary Table 1). While consistent across both the entire study set and our training set in all single time-groups excluding the 1-2 YTD samples, the largest risk was in fact observed in the 3-4 YTD subgroup (OR = 13.26 (95% CI 1.36–1781.23), *P* = 0.022) and 4+ YTD (OR = 8.43 (95% CI 1.64–84.86), *P* = 0.0090). These findings were, however, not reproducible in our test (Supplementary Fig. 10e, f and Supplementary Tables 1 and 2). Once again, diabetes had to be combined with other covariates to enhance performances (see section below on ensemble stacking of multi-marker models).

Regarding the potential effects of the fact that CA19-9 expression is absent in 8–10% of Caucasians^11^ on the performance of the classifiers presented here, please check the dedicated section to this topic in Supplementary Discussion.

We also used the best classifier developed in the UKCTOCS training set (see section below on ensemble stacking of multi-marker models) to test its predictive PDAC capacity in a subset of cases and controls collected from an external cohort, the ADEPTS study^27^ (see data description in Table 2). Regarding this subset of cases and controls collected from the ADEPTS cohort, less information was available (see Table 2). HRT and OCP use were not collected for any of the women in the study. Yet, clinical covariates such as Diabetes, Age, Gender and BMI were available (Table 2). Apart from Age (OR = 1.08 (95% CI 1.03–1.16), *P* = 0.0054), no other had an association with PDAC, but the dataset available for analysis here was relatively small.

### Ensemble learning multi-marker models improve performance

The group of base-learners chosen for the ensemble analysis reported here covered a large and diverse set of approaches (see Methods, Fig. 1a and Supplementary Fig. 23), which was beneficial, as different characteristics of the training set were captured by different classifier techniques (see comments below on variable importance attributed by each classifier). This, in turn, will increase classifier heterogeneity and therefore the likelihood of success when predicting outcomes in unseen data^19–22^. Here, we will focus on the results of the JTG2L BMA stack ensemble classifier, which was trained on all samples collected 0-1, 0-2, 0-3, 0-4 or 0-4+ YTD (described in the ‘Methods’ section and in Supplementary Fig. 24), which allows for larger numbers of sets during cross-validation, better performances in the training set and smaller confidence intervals (Supplementary Fig. 27).

For the results on other stacking ensemble strategies see the ‘Methods’ section and the Supplementary Discussion section on ensemble classifiers specialized in single time-group samples (see also Supplementary Figs. 25–27d, i).

As we increased the interval of joined time-groups from 0-1 to 0-4+, and thus the number of samples used to train, the performances in the training (Fig. 1a, Supplementary Fig. 27a, b) and the test set decreased (when the training and testing time-groups are the same, Fig. 1b and Supplementary Fig. 27c), as expected, since the median time to diagnosis increased (see also the diagonal values of the heatmap in Fig. 1c). A similar trend was roughly observed for the sensitivity, positive predictive value (PPV) and negative predictive value (NPV) achieved (see Supplementary Figs. 28a–c for the training set, and Fig. 1d–f for the test set), with the 0-2 group appearing as the outlier. The use of the JTG2L BMA stacked ensemble was beneficial both for improving performance in the test set as well as decreasing variability across training folds (Fig. 1a, Supplementary Fig. 27a). Models trained in each joined time-groups attained, nevertheless, better performances in the test set, in certain instances, when evaluated in samples belonging to narrower time-groups, e.g. AUC^test^_(0-3)_ (0-3, training) = 0.79 was considerably smaller than AUC^test^_(0-3)_ (0-4+, training) = 0.84, with the difference being borderline statistically insignificant (*P* = 0.06) (Fig. 1c, off diagonal values in the heatmap). This result probably stemmed from the additional non-specific information contained in the single 3-4 and 4+ time-groups, which helped to correct for the poor performances in the 1-2 single time-group (Supplementary Fig. 29). Under normal circumstances, when evaluating the PDAC risk for samples in newly collected data, early diagnosis would only be a comprehensive effort if evaluated with the classifier developed with 0-4+ joined time-group training set samples, as time to diagnosis is obviously not available for data collected from new patients. Reassuringly, the cross time-group performances in the test set, generated with the 0-4+ classifier, were not statistically different from those with narrower joined time-groups (*P*^*test*^_(0-1)_ (0-4+, training) = 0.66, *P*^*test*^_(0-2)_ (0-4+, training) = 0.68, *P*^*test*^_(0-3)_ (0-4+, training) = 0.06, *P*^*test*^_(0-4)_ (0-4+, training) = 0.41, when comparing the last column in Fig. 1c with the diagonal), under this broad classifier, which justifies its use in external validation sets. In addition, the JTG2L BMA stack ensemble approach outperformed the single marker and multi-marker models relying on simple logistic regression (Supplementary Figs. 21, 22); developed with 0-4+ training set samples and evaluated in the 0-1 subgroup of the test set, it reached an AUC^test^_(0-1)_(0-4+, training) = 0.94 (95% CI 0.83–1), which far exceeded the results with CA19-9 alone (AUC^test^_(0-1)_(0-1, training) = 0.73 (95% CI 0.52–0.93), Sens^test^_(0-1) _= 0.62 (95% CI 0.38–0.85) at 90% Spec.) or in combination with THBS2, MUC16 or CEACAM5 when predicting PDAC status in the same time-group (see Supplementary Figs. 21, 22 and Supplementary Discussion section on single and multi-marker combination models developed with simple logistic regression techniques). The sensitivity at 90% specificity and the respective PPV and NPV were also enhanced for the JTG2L BMA stack ensemble model (see Fig. 1g–i). An improvement in performance with respect to previous studies in pre-diagnostic samples was also achieved with samples collected up to 2 years; there a sensitivity of 0.406 at 0.905 specificity was reported^12^.

Despite any other test specificity than 100% used for asymptomatic patient screening will result in a higher rate of false positives^34^, especially in a setting of low disease incidence and prevalence such as that characterizing PDAC, this represents an ideal scenario and currently used molecular or imaging modalities are far from such performance. For CA19-9, which is the only clinically applicable marker, the performance reported in the literature ranges between 72–81% for sensitivity, at 82–90% specificity^11^, in symptomatic patients. The accuracy of cross-sectional imaging modalities, although dependent on disease stage and extension stands at 92% specificity at best^35^. Therefore, for the purpose of comparing the results obtained with the technique developed in this paper with the results reported in the literature, we chose to also report model metrics at 90% specificity.

The JTG2L BMA stack was capable of attaining a performance of 0.84 (0.72–0.93), when trained in samples belonging to the same joined time-group, i.e., 0-2 (Fig. 1b), or 0.85 (95% CI 0.74–0.93), when trained in 0-4+ samples (Fig. 1c), the difference between the two was insignificant (*P*^*test*^_(0-2)_ (0-4+, training) = 0.68). The use of the larger feature input space characterising the JTG2L BMA stack is further justified given that the significant single marker models in the training set (Supplementary Fig. 17) did not reach significance in the test set in 0-2 samples, with exception of CA19-9 and CEACAM5, both with an AUC^test^_(0-2)_ <0.75 (AUC^test^_(0-2)_(CA19-9) = 0.63 (95% 0.51–0.81), AUC^test^_(0-2)_(CEACAM5) = 0.73 (95% 0.53–0.86)). A previously published logistic regression model combining CA19-9 and MUC16 gave AUCs of 0.76 for the up to 1 year group in a previous study validation set^12^. In the current dataset, the same bi-dimensional model predicted PDAC with an AUC^training^_(0-1)_ (CA19-9, MUC16) = 0.81 (95% CI 0.72–0.88), and AUC^training^_(0-2)_ (CA19-9, MUC16) = 0.74 (95% CI 0.67–0.81), in the training set (reported in Supplementary Discussion). The same models tested at roughly the same level in the test set: AUC^test^_(0-1)_ (CA19-9, MUC16) = 0.76 (95% CI 0.55–0.93) and AUC^test^_(0-2)_ (CA19-9, MUC16) = 0.66 (95% CI 0.51–0.80) (see Supplementary Discussion section on simple multi-marker models), the latter considerably lower than the JTG2L BMA stack performance of 0.84 (trained in 0-2 samples, *P* = 0.0060) and 0.85 (trained in 0-4+ samples, *P* = 0.0061) (see Fig. 1c). The sensitivity, PPV and NPV at 90% specificity also improved with the ensemble algorithm proposed here, with sensitivities as high as 0.92 (95% CI 0.54–1), 0.61 (95% CI 0.17–0.83) and 0.63 (95% CI 0.29–0.77), and PPVs as high as 0.91 (95% CI 0.85–0.92), 0.81 (95% CI 0.55–0.85) and 0.84 (95% CI 0.70-0.87), in 0-1, 0-2 and 0-3 test samples, depending on the joined/combined time-group used to train (see Fig. 1d, i and Supplementary Figs. 10, 21 for comparison with CA19-9 and other widely referenced biomarkers). This outcome came at a cost of increased model complexity and input space dimensionality. The features selected from the 101-marker input panel are plotted in Fig. 2. The selected panel of biomarkers for the JTG2L BMA stack meta-learner developed with all time-group training samples combined (0-4+), shows typical gene ontology and pathway terms with ‘Constitutive signalling by aberrant PI3K in cancer’ [REAC], ‘Pancreatic cancer’ and ‘Proteoglycans in cancer’ [KEGG], being significantly over-represented (Fig. 3).

One of the striking aspects of the 0-4+ PDAC predictive index was the presence of commonly cited markers such as CA19-9, MUC16, THBS2, CEACAM5 and VWF and 4 of the clinical variables, excluding BMI. Diabetes was ranked just below CA19-9 according to the median importance across base-learners and time-groups, something which is consistent with what is observed in Supplementary Fig. 21 (see also the section on single biomarker association with PDAC and multi-dimensional models reported in Supplementary Discussion). Also remarkable was the variance in the importance attributed to the same markers across models developed in narrower time-groups, except for CA19-9 which almost always was allocated a scaled importance of 1. These observations are in line with those reported in the literature as strong arguments for using stacking and ensembles of classifiers, e.g., a pool of base-learners outperforms single classifiers by enabling heterogeneity within the pool of the base-learners and thus robustness in the predictions^19,21^. There is, nevertheless, a caveat to this as diversity and performance are not strictly directly proportional and while there is a strong dependency between the former and the latter, diversity might hinder performance of the ensemble^21,36^. Given that we started with a reduced pool of base-learners, the true relationship between prediction heterogeneity/diversity and performance could not be analysed extensively. Yet, upon searching all possible combinations of base-learners from the 10-dimensional input space, the use of all 10 classifiers in the stack always outperformed the rest across a set of 10 times 10-fold cross-validation strategy. Although there wasn’t a clear trend for base-learner pair-wise diversity with time-group across base-learners (see Cohen k-statistic in Supplementary Figs. 35, 36), the JTG2L BMA stack did, in fact, outperform the best base-learner in the training set (Fig. 1a), and any of the remaining stacks (see Supplementary Figs. 27, 28). This was particularly clear in the trend observed among stacks from best to worse in Supplementary Fig. 27a. From the performance across training cross-validation folds, the BMA stack is the obvious choice across time-groups and, consequently, constitutes the model of choice for validation in new data. It was interesting to note that the MEAN stack outperforms the GEOMEAN stack, a mixture of experts focusing on consensus among base-learners, and the MAX stack, choosing the maximum probability for PDAC status among the base-learners, which, effectively, amounts to highlighting the classifier that has the highest degree of certainty/highest probability of being a case for each sample. The BMA stack and the MEAN stack provide a balance between base-learners, the former being weighted, which also increased the performance in the test set, especially in the 0-1 and 0-2 joined time-groups.

One particular aspect of PDAC is its low prevalence in the general population, at 8–12 per 100,000 per year and a 1.3% lifetime risk of developing the disease^37^. The models developed and tested here relied on data where the prevalence was approximately between 40 to 50% (see Table 1 and Supplementary Table 12) and on the maximization of the ROC AUC, which is independent from prevalence. At these values, the BMA stack JTG2L reached PPVs as high as 0.91 (95% CI 0.85–0.92) in 0-1 samples and 0.84 (95% CI 0.70–0.87) in 0-3 samples (Fig. 1e). If the prevalence of the disease in the test set was changed the PPV and NPV at 90% specificity decreased and increased, respectively, at lower disease prevalence, as expected (Supplementary Fig. 37)^38^. The ROC AUC was nevertheless stable and the tendency with time-groups observed in Fig. 1 was also verified (see Supplementary Fig. 38). The larger variances at lower prevalence stem from the smaller datasets.

In addition to the test set generated from the total number of collected samples from UKCTOCS, we also evaluated the performance of the JTG2L ensemble classifier with a BMA stack in a subset of PDAC cases and controls which did not have an underlying gastrointestinal disorder, collected under the ADEPTS study^27^. These samples were post-diagnosis. Although the validation of the PDAC signature as an early detection tool is not applicable in these participants, the value of this validation lies in assessing whether the UKCTOCS PDAC signature is capable of distinguishing blinded cases from controls collected from a separate population. Since HRT and OCP use information was not available for the ADEPTS cohort, we tested the marginal performance of the best UKCTOCS classifier mentioned above (JTG2L BMA stack) by generating 1000 random allocations of Yes/No to the women in the ADEPTS subset (see Table 2) and No to all the men. This was to verify if the HRT and OCP covariates have a defining influence on the performance. Overall, the marginal performance across the classifiers developed in each time-group is above an AUC of approximately 0.84 and significant (Fig. 4a). The largest marginal performance is obtained with the JTG2L model developed in 0-2 UKCTOCS samples with a median value at 0.90 (Fig. 4a). Sensitivities, PPVs and NPVs values at 90% specificity range from median values at approximately 0.59 and 0.71, above 0.85 and below 0.90, approximately between 0.69 and 0.75, respectively, across all joined time-group models (see Fig. 4b–d as well as Supplementary Fig. 39 for the corresponding Matthew’s Correlation Coefficient values). An interesting feature of the trend observed with joined time groups is that it follows roughly from the median scaled importance attributed to HRT and OCP (see Fig. 2). Despite this observation, the dispersion observed in the marginal performance across the random samples created is low and the impact of missing HRT and OCP information is not sufficient to affect the predictive capacity of the UKCTOCS best classifier to the point of it not being significant. The performances when stratified by PDAC stage in the ADEPTS samples are not shown due to the numbers of cases in each class being low (see Supplementary Table 3) and, therefore, not reliable.

## Discussion

From the classifiers developed here, those trained with all available samples (0-4+) would be the most appropriate under a clinical setting, as time to diagnosis is not available in newly collected data. The features highlighted as predictive under these circumstances included important and widely referenced markers which we confirmed as having among the strongest association with PDAC, namely CA19-9, CEACAM5, MUC16 (CA125), THBS2 and diabetes (Fig. 2). Compared to the rest, CA19-9 was the most prominent marker as all base-learner classifiers attributed it almost always the highest importance. CA19-9 is an indicator of aberrant glycosylation in pancreatic cancer and it is considered as a biomarker, predictor, and promoter in pancreatic cancer, although it is often found elevated in benign pancreatic biliary diseases such as pancreatitis, cholangitis and obstructive jaundice, giving a substantial rise in false positives^11,39^. Moreover, CA19-9 expression is absent in 8–10% of Caucasians with a Lewis-negative blood group, as the CA19-9 epitope is in fact, the sialylated Lewis A blood group antigen^11^. Although CA19-9 levels are routinely used in detection, determination of resectability and monitoring of PDAC progression^40,41^, its low predictive value and a low prevalence of pancreatic cancer in the general population exclude it as a robust screening tool^3,42^. The combination of CA19-9 with other markers proposed here is, therefore, advantageous. Regarding CEACAM5 and the role of carcinoembryonic (CEA) related cell adhesion molecules (CEACAMs) 1, 5 and 6 in progression of solid tumours (such as colorectal, lung, melanoma, breast, liver) including pancreatic cancer is well established, and their expression varies between different tumour histological subtypes^43–45^. With respect to pancreatic cancer, CEACAM5 has been widely described as having a variable diagnostic value in PDAC detection, while its expression has also been reported to inversely correlate with disease stage^46^. In a recent report, CEACAM5 was found to be persistently elevated up to 26.5 months prior to pancreatic cancer diagnosis, in a cohort of longitudinally sampled participants^47^. The predictive performance of CEACAM5 as a single analyte in pancreatic cancer, however, is poor^44^. In our work, CEACAM5 taken as a single predictor achieves only significant performances in 0-1 YTD single time-group samples but is among the top covariates in terms of importance across base-learners, which confirms the necessity for non-linear multi-marker models.

MUC16/CA125 also ranked high in importance across the base-learner classifiers and time-groups despite not performing well as a single predictor. MUC16 is a cell surface glycoprotein which can be elevated in tissue and sera of patients with various cancers and is mostly used in the diagnosis and prognostication of ovarian cancer^48,49^. MUC16-mediated metabolic reprogramming in pancreatic cancer is associated with cellular invasion and motility^49^. Due to its overexpression on the surface of pancreatic cancer and absence from normal tissue, the value of MUC16 as a biomarker of PDAC has been investigated^50^. A progressive change in expression of MUC16 throughout different stages of disease progression is already evident at pre-malignant (pancreatic intra-epithelial neoplasia; PaNIN) stages, highlighting its potential value as a diagnostic marker of early cancer^48,51^. With respect to its diagnostic performance, one meta-analysis which included 1235 patients reported a pooled sensitivity of 0.59 (95% CI 0.54–0.62; at 0.78 specificity, 95% CI 0.75–0.82) for detecting PDAC using CA125 (an epitope of MUC16)^52^ as a single marker^53^. When combined with CA19-9 (AUC 0.85) CA125 increased the overall accuracy of the former (AUC 0.89), demonstrating superior diagnostic accuracy compared to CA125 (or CA19-9) alone^53^. The clinical value of MUC16 in prediction of metastasis and prognosis in PDAC has been also previously reported^54,55^. Serum levels of MUC16/CA125 have been shown to be the strongest predictor of metastatic disease (AUC of 0.892 at 95% CI 0.846–0.938, *p* < 0.001) and survival (HR: 1.804, 95% CI 1.22–2.66, *p* = 0.003) compared to other markers (including CEA) in 180 PDAC patients^55^.

THBS2 and THBS1 were both significantly associated with PDAC, albeit not across all time-groups, the latter only in a subset of samples (see Supplementary Discussion section on PDAC signatures developed with additional proteomic data for a sub-group of participants for the discussion on the performances associated with these models). Thrombospondins are glycoproteins which mediate cancer growth and progression through cell-cell and cell-matrix interactions, tissue remodelling and regulation of inflammation, immunity, and angiogenesis^56,57^. A specific role for THBS2 in tumour-associated vascularisation has been reported in various cancers (including colon, liver, lung and melanoma) in which its aberrant expression was reported to be of both diagnostic and prognostic value^56,58^. With respect to PDAC, recent observations in a cohort of 493 (263 with PDAC) showed that THBS2 serum levels significantly differed and differentiated PDAC from high-risk individuals (familial pancreatic cancer patients) with a 55.9% test sensitivity (at 100% specificity; 100% PPV and 66.5% NPV). Moreover, PDAC patients with higher serum THBS2 (also termed TSP-2) levels showed worse clinical outcomes (hazard ratio = 1.54, 95% CI 1.143–2.086, *P* = 0.005). Interestingly, when combined with CA19-9 an improved panel sensitivity for PDAC (90.5% at 98.7% specificity) was reported^56^. Taken as part of a signature as was presented here, THBS2 is expected to help robustly with early detection of PDAC in other datasets.

In this study, among all interrogated clinical covariates, diabetes was the strongest predictor of a higher risk for PDAC (OR = 13.26, 95% CI 1.36–1781.23, *P* = 0.022), in our 3-4+ YTD cohort. An association between long-standing type 2 diabetes and a 1 to 1.5-fold increased risk of PDAC compared to non-diabetics has been reported^59,60^. The value of diabetes as a clinical covariate in a multi-marker panel is supported by the fact that the presence of new onset diabetes (NOD) (less than 3 years prior to diagnosis of PDAC) increases this risk 5-fold and is induced by various pancreatic conditions including chronic pancreatitis and PDAC-induced hyperglycaemia (recently described as type 3c diabetes). Abnormal fasting glucose or glucose intolerance are observed in the majority (>80%) of PDAC patients^3,60,61^; PDAC as an underlying cause of NOD, is found in around 1% of individuals aged over 50 and therefore NOD might be considered an early warning sign of a pancreatic malignancy^61,62^. Not having detailed information on duration and type of diabetes for all participants selected for the current study was an unavoidable aspect given that it was not known at the time of sample collection (see ‘Results’). Therefore, the distinction between PDAC marker signatures present in long-standing type 2 diabetes and NOD was not possible. Had we had detailed information, the specific importance of biomarkers with respect to diabetes status would have been improved and adapted signatures for populations at a higher risk^3,63^ been determined. We should emphasize, nevertheless, that collection of this information was not within the aims of the main UKCTOCS trial focusing on ovarian cancer.

Despite numerous reports of recent biomarker discoveries, lack of standardisation in sample identification and handling, methodologies of analysis as well as data capture and bioanalytical interpretation challenge their later validation in larger cohorts^64^. Moreover, due to the biological complexity in PDAC, tested biological fluids and the overlapping features with high-risk conditions (e.g. chronic pancreatitis), the predictive capabilities of single analytes are clearly insufficient to meet acceptable diagnostic performances^65^. Considering their individual, relative poor performance (79–81% test sensitivity and 82–90% specificity in the case of CA19-9 for example) as well as the low prevalence of PDAC in the general population, accepting low values for test sensitivity and specificities results in an increased number of false positives. The combination of multiple analytes in diagnostic panels clearly enhances CA19-9 performance, as well as being able to compensate for cases in which CA19-9 detection is limited (Lewis body negative patients)^66^. A mere combination of multiple biomarkers with low sensitivities at high specificities based on individual levels, however, would be insufficient, and their independent contribution to the overall risk should be considered^67^. With this in mind, the aim of the ensemble modelling strategy explored here was to improve on single classifiers by combining diverse techniques in a way such that the predictive performance of the ensemble would be greater and more robust in newly collected datasets when resorting to multi-marker models, thus addressing the issues highlighted above. The pool of chosen classifiers proved to be useful in highlighting different aspects of the data by selecting a larger panel of biomarkers and allocating different importance to each. Despite finding that the stacking protocols explored offered substantial and statistically significant improvements over the previous state-of-the-art prediction methods, we expect that scanning the space of all classifier combinations from available code libraries will improve the results. Yet, we must resort to other methods for finding better performing ensembles when predicting PDAC. Early detection is a notoriously difficult problem due to the extreme class imbalance, missing values and the necessity to create predictive indices performing well at representative disease prevalence. Because the robustness of ensembles is an emergent, not an explicit property, different directions for future work should be taken by formalizing the effects of calibration on heterogeneous ensemble performance and explicitly incorporating diversity in the search^21,68^. In fact, this will be even more prominent when combining longitudinal methods^69^ with ensemble selection and stacking techniques. To our knowledge, longitudinal samples and associated time-series classification techniques have never been applied to early detection of pancreatic cancer. Dynamic changes of CA19-9 and MUC16 identified in previous work^12^ are an indication of the importance of these types of studies. The importance of trends and rates of change in each biomarker taken separately or in multi-marker models has also proven to be advantageous in improving early detection in ovarian cancer longitudinal models^69^ and mechanistic tumour and biomarker secretion models^70^. Further developments in PDAC longitudinal datasets and ensemble model selection routines should highlight the importance of early and late biomarker panel dynamic changes and increase performance in a clinical setting, in addition to contributing with invaluable methodologies to the field of machine learning and artificial intelligence in early detection of cancer^71^.

Our study based on UKCTOCS samples has several limitations mostly related to participant gender. UKCTOCS samples were only collected from postmenopausal women and the classifiers developed might not reflect the levels of biomarkers associated with pancreatic tumours in the general population, especially when males are at a higher risk^72^. We have, nevertheless, proven that at least in samples collected in an independent, post diagnosis cohort (recruited under the ADEPTS study)^27^, the performances are comparable to those observed in the UKCTOCS test set presented here, in samples closer to diagnosis.

The importance associated with HRT in the ensemble technique described here might not be a characteristic of the UKCTOCS cohort only. HRT has, in fact, been observed to reduce the risk of pancreatic cancer^73^. OCP use, on the other hand, might reduce risk in postmenopausal women but the results at this point are unclear^74^. Another drawback of the current study lies with the lack of or insufficient information on grading, staging, tumour size, and diabetes type, but cohorts with samples taken years before diagnosis are scarce. The work reported here corresponds to a discovery phase of the performance of an ensemble modelling technique and the panel of markers that were available for the study. Alternative datasets collected from external longitudinal cohorts such as that being currently generated for the Pancreatic Cancer Early Detection Consortium (PRECEDE)^75^ will constitute a viable longitudinal external validation alternative, albeit with a caveat, all individuals have a family history of PDAC and/or are carrying pathogenic/likely pathogenic germline variants in genes linked to PDAC.

## Supplementary information

Description of Additional Supplementary Files

Supplementary Information

Supplementary Data 1

Supplementary Data 2

Supplementary Data 3

Supplementary Data 4

Supplementary Data 5

Supplementary Data 6

Supplementary Data 7

Reporting Summary

## Data Availability

Data requestors will need to sign a data access agreement and in keeping with patient consent for secondary use obtain ethical approval for any new analyses. Source data for the main figures are available in excel format as Supplementary Data 1, 7.

## Code availability

All packages used in the pre-processing of data and subsequent analysis have been identified, together with their versions, to secure full reproducibility. We have also identified the resampling strategies in detail and all base-learner stacking approaches tested are also described in detail in the ‘Methods’ section and in Supplementary Figures (see Supplementary Figs. 24–26). The application of each of the used algorithms follows the respective package recommendation. The stacking strategy with joined time-groups JTG2L with over-sampling of the minority class and synthetic data generation with HD-DPM or SMOTE are available in R^76^. For the results obtained with the JTG2L ensemble (see Fig. 1 and Supplementary Fig. 24), see Supplementary Table 13 for the optimal hyperparameters found through a random selection of 1000 combinations for each base-learner.
