# Supplementary material for: Serum biomarker-based early detection of pancreatic ductal adenocarcinomas with ensemble learning
Source: Commun Med (Lond). 2023 Jan 20;3:10. doi: 10.1038/s43856-023-00237-5 (PMC9860022; doi:10.1038/s43856-023-00237-5)
Supplement: Supplementary file 1 — Description of Additional Supplementary Files [file 43856_2023_237_MOESM1_ESM.pdf]

Description of Additional Supplementary Files

File Name: Supplementary Data 1

Description: numerical results allowing for the reproduction of Figure 1a

File Name: Supplementary Data 2

Description: numerical results allowing for the reproduction of Figure 1b,d,e,f

File Name: Supplementary Data 3

Description: numerical results allowing for the reproduction of Figure 1c,g,h,i

File Name: Supplementary Data 4

Description: numerical results allowing for the reproduction of Figure 2a

File Name: Supplementary Data 5

Description: numerical results allowing for the reproduction of Figure 2b

File Name: Supplementary Data 6

Description: numerical results allowing for the reproduction of Figure 3

File Name: Supplementary Data 7

Description: numerical results allowing for the reproduction of Figure 4
