# Supplementary material for: Serum biomarker-based early detection of pancreatic ductal adenocarcinomas with ensemble learning
Source: Commun Med (Lond). 2023 Jan 20;3:10. doi: 10.1038/s43856-023-00237-5 (PMC9860022; doi:10.1038/s43856-023-00237-5)
Supplement: Supplementary file 2 — Supplementary Information [file 43856_2023_237_MOESM2_ESM.pdf]

Supplementary Information

Title: Serum biomarker-based early detection of pancreatic ductal adenocarcinomas with ensemble learning

Authors: Nuno R. Nené[1,*], Alexander Ney[2], Tatiana Nazarenko[1,3], Oleg Blyuss[1,4], Harvey E. Johnston[1,5], Harry J. Whitwell[1,6,7], Eva Sedlak[1], Aleksandra Gentry-Maharaj[8], Sophia Apostolidou[8], Eithne Costello[9], William Greenhalf[10], Ian Jacobs[1,11], Usha Menon[8], Justin Hsuan[2], Stephen P. Pereira[2], Alexey Zaikin[1,3], John F. Timms[1,12].

Affiliations:

[1] Department of Women's Cancer, EGA Institute for Women's Health, University College London, 84-86 Chenies Mews, London, WC1E 6HU, UK.

[2] Institute for Liver and Digestive Health, University College London, Upper 3rd Floor, Royal Free Campus, Rowland Hill Street, London NW3 2PF, UK.

[3] Department of Mathematics, University College London, London WC1H 0AY, UK.

[4] Wolfson Institute of Population Health, Queen Mary University of London, Charterhouse Square, EC1M 6BQ, London, UK

[5] Babraham Institute, Babraham Research Campus, Cambridge, CB22 3AT, UK

[6] National Phenome Centre and Imperial Clinical Phenotyping Centre, Department of Metabolism, Digestion and Reproduction, IRDB Building, Imperial College London, Hammersmith Campus, London, W12 0NN, UK

[7] Section of Bioanalytical Chemistry, Division of Systems Medicine, Department of Metabolism, Digestion and Reproduction, Imperial College London, South Kensington Campus, London, SW7 2AZ, UK

[8] MRC Clinical Trials Unit at UCL, Institute of Clinical Trials and Methodology, UCL, 90 High Holborn, 2nd Floor, London, WC1V 6LJ, UK

[9] Department of Molecular and Clinical Cancer Medicine, University of Liverpool, Liverpool, UK

[10] Liverpool Experimental Cancer Medicine Centre, University of Liverpool, Liverpool, L69 3GL, UK

[11] University of New South Wales, Sydney, NSW, 2052, Australia

[12] Deceased

*To whom correspondence should be addressed:

Dr Nuno R. Nené (Email: nuno.nene.10@ucl.ac.uk), Institute for Women's Health, University College London, Cruciform Building 1.1, Gower Street, London, WC1E 6BT. Tel: +44 207 679 6598.

In this supplementary information we provide the additional tables and figures cited in the main text, as well as an extensive analysis of single biomarker association and previously reported multi-dimensional models. We also provide further results of ensemble classifiers specialized in single time-group samples, the effects of training the base-learners with real and synthetic data and the effect of samples not expressing CA19-9 on the performance of the stacked classifiers. For the sake of completeness, we also plot the performance results and the pancreatic ductal adenocarcinoma (PDAC) signatures developed with additional biomarker proteomic data that was generated only for a sub-group of participants.

Supplementary Discussion

Single biomarker association with PDAC and previously reported multi-dimensional models

Levels of the established tumour markers CA19-9, MUC16 and CEACAM5 showed a significant association with PDAC in samples taken less than 1 years to date (YTD) (Supplementary Figures 10 and 12), under a logistic regression model developed in the training set (see details in the Methods section), which is consistent with previous reports [1]. Notably, THBS2 also appeared to have a predictive value in training set samples taken up to a year to diagnosis (OR=1.73 (95% CI 1.17- 2.70), *P*= 0.0054, AUC=0.65 (95% CI 0.55-0.75)) (Supplementary Figures 10 and 12), between 1 to 2 years (OR=2.34 (95% CI 1.23 - 4.87), *P*= 0.0081, AUC=0.67 (95% CI 0.55-0.79)) (Supplementary Figures 10 and 13), and above 4 YTD (OR=2.63 (95% CI 1.17 - 6.84), *P*= 0.018, AUC=0.67 (95% CI 0.52-0.82)) (Supplementary Figures 10 and 16). A link between THBS2 and an increased risk for pancreatic cancer has been reported before [2] and was shown to improve the ability of CA19-9 to distinguish PDAC from pancreatitis, with a specificity of 98% and a sensitivity of 87%. Samples in that report, however, were collected after diagnosis. The samples used in our work were all gathered prior to diagnosis, the latest approximately 1 month before. THBS2, nevertheless, did not generate significant AUCs in the test set, whether with logistic regression models trained with single time-groups (Supplementary Figure 10) or combined time-groups (see also Supplementary Figure 12 and 17 to 20). The only other marker that remained a consistent predictor in more than one single time-group was CA19-9: $OR^{training}_{(0-1)}$=1.67 (P=2.92 x 10^{-9}), $AUC^{training}_{(0-1)}$=0.81 (95% CI 0.71 - 0.89) and $Sens^{training}_{(0-1)}$= 0.64 (95% CI 0.48 - 0.77) at 90% Spec; $OR^{training}_{(1-2)}$=1.67 (P=2.92 x 10^{-9}), $AUC^{training}_{(1-2)}$=0.66 (95% CI 0.53-0.78), $Sens^{training}_{(1-2)}$= 0.31 (95% CI 0.10 - 0.49) at 90% Spec; $OR^{training}_{(2-}$

$_{3)}$=1.60 (P=0.0017), $AUC^{training}_{(2-3)}$=0.71 (95% CI 0.59-0.83), $Sens^{training}_{(2-3)}$= 0.33 (95% CI 0.061 - 0.61) at 90% Spec (Supplementary Figure 10). When attempting to predict PDAC status in the test set with the logistic regression models trained with time-group 0-1 samples and used to rank biomarkers, we still verified that CA19-9 was one of the two biomarker models that achieved significant performances: $AUC^{test}_{(0-1)}$=0.73 (95% CI 0.52 - 0.93) , $Sens^{test}_{(0-1)}$= 0.62 (95% CI 0.38 - 0.85) at 90% Spec. The other was CEACAM5: $OR^{training}_{(0-1)}$=1.94 (95% CI 1.32 - 3.08), P= 0.00029 and $AUC^{training}_{(0-1)}$= 0.67 (95% CI 0.56 - 0.77) in the training set and $AUC^{test}_{(0-1)}$=0.84 (95% CI 0.65 -0.97) in the test set (see Supplementary Figure 10). The combination of CA19-9 and THBS2 improved the performance in both training and the test sets, for 0-1 samples, i.e., $AUC^{training}_{(0-1)}$ (CA19-9, THBS2) = 0.82 (95% CI 0.73 - 0.89) and $AUC^{test}_{(0-1)}$ (CA19-9, THBS2) = 0.74 (95% CI 0.51 - 0.92), although the difference with respect to the CA19-9 single marker model was not significant, i.e., P=0.19 and P= 0.69, respectively, under a DeLong method for comparing AUCs from 2 different ROC curves. Additionally, the combined 2-marker model CA19-9+CEACAM5 did not improve the diagnostic performance of CA19-9 alone in the training set ($AUC^{training}_{(0-1)}$=0.80 (95% CI 0.71-0.88)), but improved in the test set, i.e., $AUC^{test}_{(0-1)}$=0.81 (95% CI 0.62 - 0.95), although not sufficiently for the difference to be significant (P=0.24). MUC16/CA125, which was previously reported to provide additional sensitivity when combined with CA19-9 in a pre-diagnosis setting similar to ours [1], was ranked as significant in our study, with an $OR^{training}_{(0-1)}$=2.72 (95% CI 1.66 - 5.01), P= 8.7 x 10^{-6}, $AUC^{training}_{(0-1)}$= 0.72 (95% CI 0.62 - 0.82) and $Sens^{training}_{(0-1)}$= 0.64 (95% CI 0.29 - 0.67) at 90% Spec, but was borderline insignificant when tested against blinded samples, i.e., $AUC^{test}_{(0-1)}$=0.72 (95% CI 0.49 - 0.90). As was the case of the combined model with THBS2, the bi-dimensional model CA19-9+MUC16 had a similar performance to the CA19-9, i.e., $AUC^{training}_{(0-1)}$ (CA19-9, MUC16) = 0.81 (95% CI 0.72 - 0.88) and $Sens^{training}_{(0-1)}$= 0.58 (95% CI 0.46 - 0.75) at 90% Spec, and therefore with no significant improvement in performance (P= 0.91). When evaluated in the test set, the CA19-9 and MUC16 multi-marker model outperformed CA19-9 and the combination of CA19-9 and THBS2, i.e., $AUC^{test}_{(0-1)}$ (CA19-9, MUC16) = 0.76 (95% CI 0.55 - 0.93), but not significantly, P=0.76 and P=0.80, respectively. The CA19-9+CEACAM5 model was, therefore, the best multi-marker predictor in the test set built from frequently cited markers but once again the difference was not significant when compared to CA19-9 alone (P=0.44). We must stress that any of the bi-dimensional models reported above did not involve any interaction terms because if we resorted to non-linear terms in

the training set the results were not significant for any combinations of the typical panel of analytes reported in the literature.

Several other multi-marker combinations have been put forward to add value to traditionally used panels [3] (see Supplementary Figure 10 for the typical single biomarkers used). In fact, as was observed in the training set 0-4+ samples, a large group of markers appeared significant: CA19-9, VWF, THBS2, MUC16, HGF, CEACAM5, VEGFA, FURIN, CTSV, NT5E, TLR3 (see Supplementary Figure 20). These findings further stimulated the exploration of multi-dimensional models in order to reach higher performances and robustness across all time-groups. If we resorted once again to the simple logistic regression approach and generated models with up to 3 features, selected from those reported in the literature [3-5], e.g., CA19-9, CA125, CEACAM5, HGF, THBS2, CEACAM1, included clinical covariates and chose the top 10 according to their performance across training cross-validation folds, we concluded that despite generating a significant performance in the test set, a clear advantage in building models from this restricted pool of features was lacking, especially when the sensitivity at 90% specificity values were relatively low (see Supplementary Figure 21 and, additionally, Supplementary Figure 22 for results obtained with single time-groups). What was notable about these 3-marker models was the presence of the covariate DIABETES and AGE in top ranks, as we had predicted (see Main Text). DIABETES was a predictor of PDAC in samples allocated to all single time-groups in the training set, with exception of 1-2 YTD (see Supplementary Figure 10). There was, therefore, the need for a systematic search over a larger panel, in our work composed of in-house and Olink biomarkers (see Supplementary Table 11), and for the use of several different classifier types which we combined with an ensemble modelling technique (see Methods and Results sections in the Main Text).

Ensemble classifiers specialized in single time-group samples

We also developed classifiers specialized in each single time-group, i.e. 0-1, 1-2, 2-3, 3-4 and 4+, here referred to as STG2L and STG3L (see Statistical analysis in Methods section for details and Supplementary Figures 25 and 26), by resorting to the same framework, i.e. stacked ensemble with repeated cross-validation and identification of optimal size of input space, and combining the probability/risk of being a

case of each sample generated by each specialized model either by taking only the output that had the maximum probability, by averaging, i.e. with a Bayesian model or a simple arithmetic average, or utilizing a mixture of experts, in our case, by calculating the geometric probability mean across all models. The latter is more conservative since a participant is only classified as a Case if there is a central tendency in the probability vector of outputs, both across base-learner and single-time group. Both the maximum probability principle as well as the mixture of experts underachieved considerably in the test set when compared with the standard approach, the JTG2L (see Supplementary Figures 27 and 28). The problem with training specialized ensembles per single time-group lies with the sample size. Their performance in the training set indicates that these alternative models, despite the extensive re-sampling strategy are not robust both at the base-learner level (Supplementary Figure 23) and the meta-learner level (Supplementary Figures 27 and 28). Consequently, their potential as predictive indices is reduced systematically, despite the overall pair-wise diversity among base-learner predictions being high in the test set (Supplementary Figure 35, see also Supplementary Figure 34 for feature importance). Further expansion of the dataset should clarify if the use of single time-group specialized classifiers is advantageous. This is particularly important if a dataset is fully longitudinal, i.e., every single participant has multiple samples up to but excluding the date of diagnosis.

Performance considering Lewis positive or negative potential status

Another aspect of the data that should be reported is the presence of outliers with respect to CA19-9. Despite not having been confirmed, let us assume here that Lewis-antigen negative status for each participant could be determined by the lack of expression of CA19-9, verified by a peak at the minimum value in the data used here. Since CA19-9 is not expressed in 8-10% of Caucasians (Lewis-negative blood group), predictive indices should account for this phenomenon. The proportion of individuals in which CA19-9 was not detected was approximately the same in the training and test sets and, apart from samples belonging to the 0-1 single time-group, there was no significant association with PDAC status in the training set (Supplementary Figure 11). Furthermore, logistic regression models, trained to distinguish cases from

controls, based on the presence or absence of measurable CA19-9, did not perform well in the test set, with performances always around AUCs of 0.5 lacking statistical significance (Supplementary Figure 11).

Regarding the importance of developing a classifier that is reliant on features covering both the population that expresses CA19-9 (Lewis-positive) and does not express CA19-9 (Lewis-negative), we also tested the BMA stack model in the subset of participants in the test set that are Lewis-positive. Overall, the BMA stacked ensemble with joined-time groups was not affected substantially and, for example, a performance in the test set of AUC=0.89 (95% CI 0.70 -1.00) in 0-1 samples and of AUC= 0.83 (95% CI 0.70 – 0.93) in 0-2 samples was obtained. This confirms that the association between the CA19-9 peak at the level associated with absence of expression and PDAC was not affecting the results. Further tests in larger pools of confirmed Lewis-negative samples should clarify the performance of the ensembles developed here.

Training the base-learners with real and synthetic data

For the purposes of exploring the effects of re-balancing the imbalanced classes, in our work the PDAC and Control classes, across all time-groups, we also developed other classifiers under the JTG2L paradigm (see Methods for details and Supplementary Figures 24) but trained in both real and synthetic data that we generated by fitting a high-dimensional Dirichlet Process Gaussian Mixture (HD-DPM) Model [6] (with the *sklearn.mixtrue.BayesianGaussinaMixture* class, https://scikit-learn.org/stable/modules/mixture.html , sklearn version 1.1.2,) model to the cases of the training set and, independently, to the controls. The high-dimensionality character of the HD-DPM method is required to capture cross-biomarker correlations, which would not have been preserved had we fitted a one-dimensional DPM model to each biomarker independently. For the sake of completeness, we also tested the Synthetic Minority Oversampling Technique (SMOTE) [7] (http://www2.uaem.mx/r-mirror/web/packages/DMwR/index.html, version 0.4.1). The inclusion of the routines for synthetic data generation was done during re-sampling and prior to the application of each stacked algorithm. This is particularly important for the HD-DPM algorithm since a separate distribution is fitted to PDAC cases and controls. The number of synthetic samples had to be similar to those of real samples, otherwise the classifiers learn the distribution of the synthetic data instead.

These additional approaches did not improve the performance either in the training set or the test sets (see Supplementary Figures 31 and 32) and thus renders the option of synthesizing data and enlarging the number of representative examples to aid the training process not viable. Both techniques were only run for joined time-groups, as the number of samples in each single time-group was not sufficient to implement either the HD-DPM or the SMOTE algorithm. Moreover, there is evidence that for high-dimensional problems techniques such as over-sampling/under-sampling of the minority/majority class outperforms methodologies such as SMOTE [7]. We also tested the ROSE algorithm [8] for synthesizing data during cross-validation resampling steps, a technique similar to HD-DPM but which does not provide the benefits of non-parametric modelling. Overall, the results obtained with ROSE were far inferior to any of the other 3 algorithms, and as is the case for synthetizing samples with HD-DPM, a lengthy process. Therefore, we ran the remaining models with the oversampling of the minority class as a strategy to develop all the ensembles of base-learners reported in the Main Text.

PDAC signatures developed with additional proteomic data for a sub-group of UKCTOCS participants

In addition to the data set used to generate the models reported in the Main Text, we also had additional biomarkers that had been measured only in sub-groups of samples. Sub-set I includes the biomarkers AFP, TEK and IGFBP1 (see Supplementary Figure 2 to 5 and Supplementary Tables 5, 6 and 7), while sub-set II includes the additional biomarkers LRG1, PIGR, REG3A, F12, AGR2, IL17RA, SERPINA1 and THBS1 (see Supplementary Figures 6 to 8 and Supplementary Tables 8, 9 and 10). These were biomarkers motivated by a previous paper [1]. The BMA ensemble stack developed with joined time-group samples in sub-set I of the training set showed inferior performances compared to the original model (Supplementary Figures 4 and 5), whereas the stack developed in the sub-set II improved the predictive potential in sub-set II of the test set, with performances as high as 0.94 (95% CI 0.75-1.00) and 0.88 (95% CI 0.71-0.99), in 0-1 and 0-2 samples, respectively (Supplementary Figures 7 and 8). Also noteworthy is the importance attributed to each new feature across base-learners (Supplementary Figure 9). For the models developed

in sub-set II, 4 of the new biomarkers are ranked in the top 20 markers by feature importance across joined time-groups: IL17RA; AGR2; SERPINA1 and LRG1. REG3A [1] and THBS1[3] have been studied before in association with PDAC. REG3A was found to be a late marker adding little to combined models with CA19-9 and MUC16 [1]. Despite this, it is attributed an importance different from zero across the ensemble classifier developed here (Supplementary Figure 9). Further developments in the UKCTOCS dataset presented here, for example the measurement of biomarkers AFP, TEK, IGFBP1, LRG1, PIGR, REG3A, F12, AGR2, IL17RA, SERPINA1 and THBS1 in all samples, will constitute an important step towards verifying if increased performances can be attained.

Supplementary Figures:

Supplementary Figure 1 Characteristic distribution for Body Mass Index (BMI) and age in the training and test sets. a and **b** Single time-groups. **c** and **d** Joined time-groups. P values were calculated according to a logistic regression model with a bias reduction method. The purple dashed line corresponds to -Log [0.05]. Performances, including Sensitivity (Sens), Positive Predictive Value (PPV) and Negative Predictive Value (NPV) at 90% Specificity (Spec) were determined with the respective unidimensional feature model developed in the training set. The Receiver Operating curve (ROC) Area Under the Curve (AUC) significance threshold is also represented by a purple dashed line at 0.5. Error bars in figures corresponding to the test set pertain to 95% Confidence Intervals (CI) for AUCs, calculated by stratified bootstrapping 2000 times. For a, the number of independent samples was n=107 (0-1), n=73 (1-2), n=72 (2-3), n=57 (3-4) and n=54 (4+). For b, the number of independent samples was n=26 (0-1), n=34 (1-2), n=22 (2-3), n=16 (3-4) and n=16 (4+). For c n=180 (0-2), n=252 (0-3), n=309 (3-4) and n=363 (0-4+). For c n=180 (0-2), n=252 (0-3), n=309 (3-4) and n=363 (0-4+). For d n=60 (0-2), n=82 (0-3), n=98 (3-4) and n=114 (0-4+). See Supplementary table 12 for further details on case and control samples. See Statistical Analysis in Methods (main text) for further details.

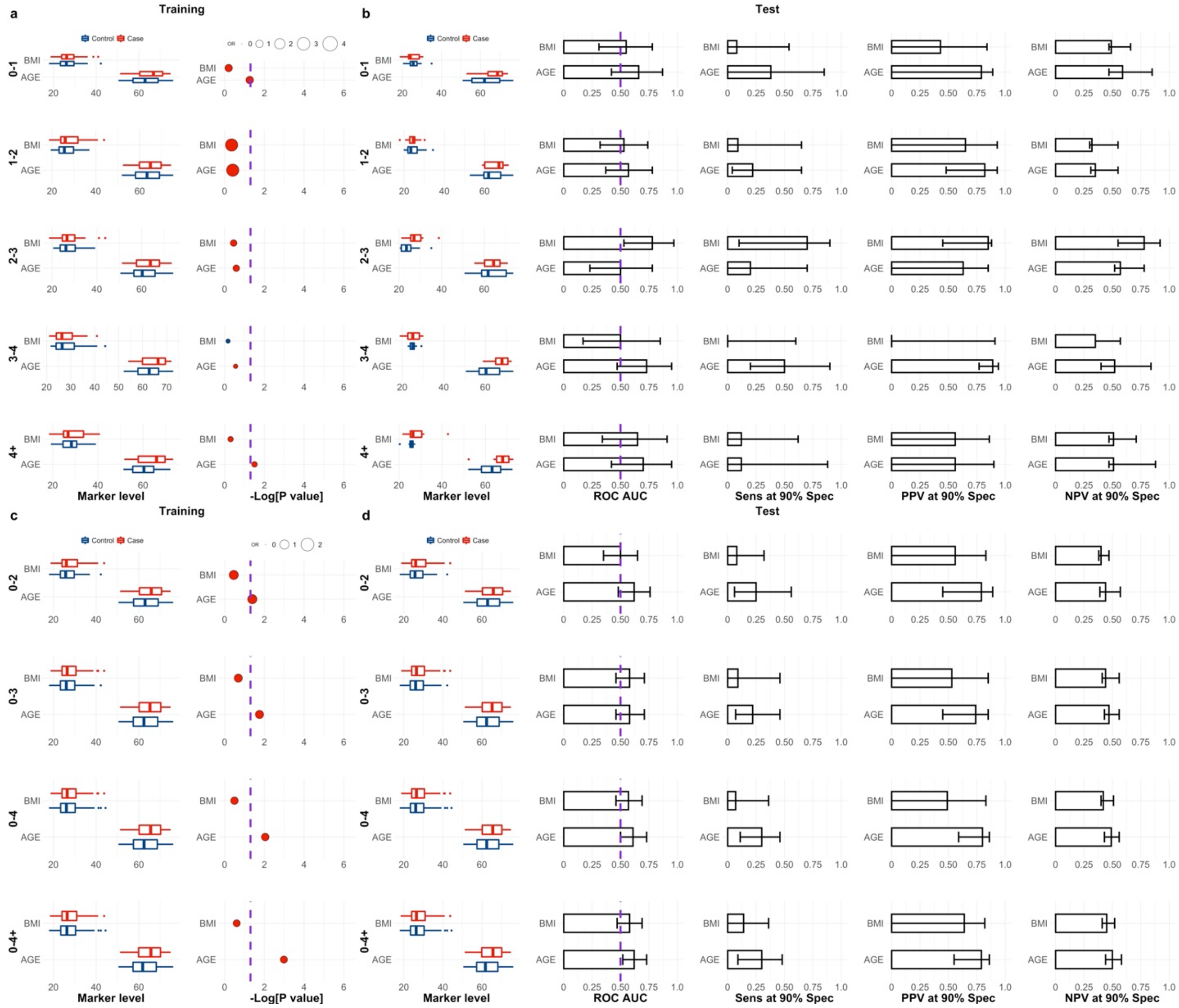

Supplementary Figure 2 Characteristic proportions for DIABETES, Hormone Replacement Therapy (HRT) and Oral Contraceptive Pill (OCP) use in the training and test sets. a and **b** Single time-groups. **c** and **d** Joined time-groups. P values were calculated according to a logistic regression model with a bias reduction method. Purple dashed line corresponds to -Log[0.05]. Performances, including Sensitivity (Sens), Positive Predictive Value (PPV) and Negative Predictive Value (NPV) at 90% Specificity (Spec) were determined with the respective unidimensional feature model developed in the training set. The Receiver Operating curve (ROC) Area Under the Curve (AUC) significance threshold is also represented by a purple dashed line at 0.5. Error bars in figures corresponding to the test set pertain to 95% Confidence Intervals (CI) for AUCs, calculated by stratified bootstrapping 2000 times. For a, the number of independent samples was n=107 (0-1), n=73 (1-2), n=72 (2-3), n=57 (3-4) and n=54 (4+). For b, the number of independent samples was n=26 (0-1), n=34 (1-2), n=22 (2-3), n=16 (3-4) and n=16 (4+). For c n=180 (0-2), n=252 (0-3), n=309 (3-4) and n=363 (4+). For c n=180 (0-2), n=252 (0-3), n=309 (3-4) and n=363 (4+). For d n=60 (0-2), n=82 (0-3), n=98 (0-4) and n=114 (0-4+). See Supplementary table 12 for further details on case and control samples. See Statistical Analysis in Methods (main text) for further details.

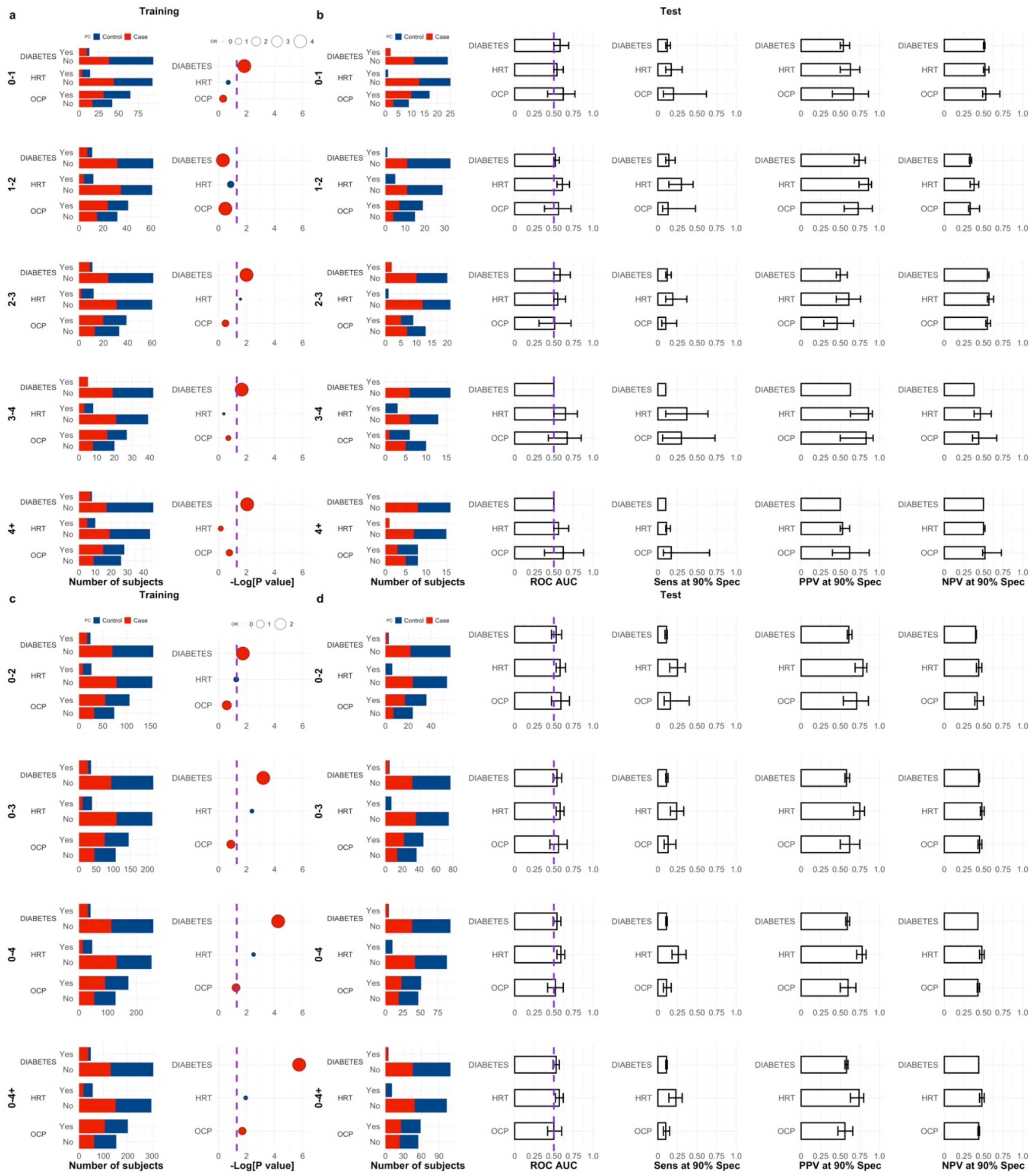

a
Training
b
Test
c
Training
d
Test
PC Control Case
0-1
1-2
2-3
3-4
4+
0-2
0-3
0-4
0-4+
DIABETES
HRT
OCP
Yes
No
Number of subjects
-Log[P value]
ROC AUC
Sens at 90% Spec
PPV at 90% Spec
NPV at 90% Spec

Supplementary Figure 3 Characteristic proportions for DIABETES, HRT and OCP, and feature ranks in the training set per joined time group according to a logistic regression model with a bias reduction method for a subset of UKCTOCS participants for which AFP, TEK and IGFBP1 was measured. a, c and **e** Distribution and ranks of biomarkers by p values in the training set. The purple dashed line corresponds to -Log[0.05]. **b** Performance of single marker models in the samples belonging to the sub test set. OR stands for odds-ratio. Red and blue OR points represent OR > 1 (favours PDAC status) and OR < 1 (favours Control status), respectively. P values were calculated according to a logistic regression model with a bias reduction methods. Performances, including Sensitivity (Sens), Positive Predictive Value (PPV) and Negative Predictive Value (NPV) at 90% Specificity (Spec) were determined with the respective unidimensional feature model developed in the training set. The Receiver Operating curve (ROC) Area Under the Curve (AUC) significance threshold is also represented by a purple dashed line at 0.5. Error bars in figures corresponding to the test set pertain to 95% Confidence Intervals (CI) for AUCs, calculated by stratified bootstrapping 2000 times. For a, c and e the number of independent samples was n=74 (0-1), n=131 (0-2), n=189 (0-3), n=225 (0-4), n=258 (0-4+). For b, d and f n=18 (0-1), n=43 (0-2), n=62 (0-3), n=72 (0-4), n=76 (0-4+). See Supplementary tables 5,6 and 7 for further details on case and control samples. See Statistical Analysis in Methods (main text) for further details.

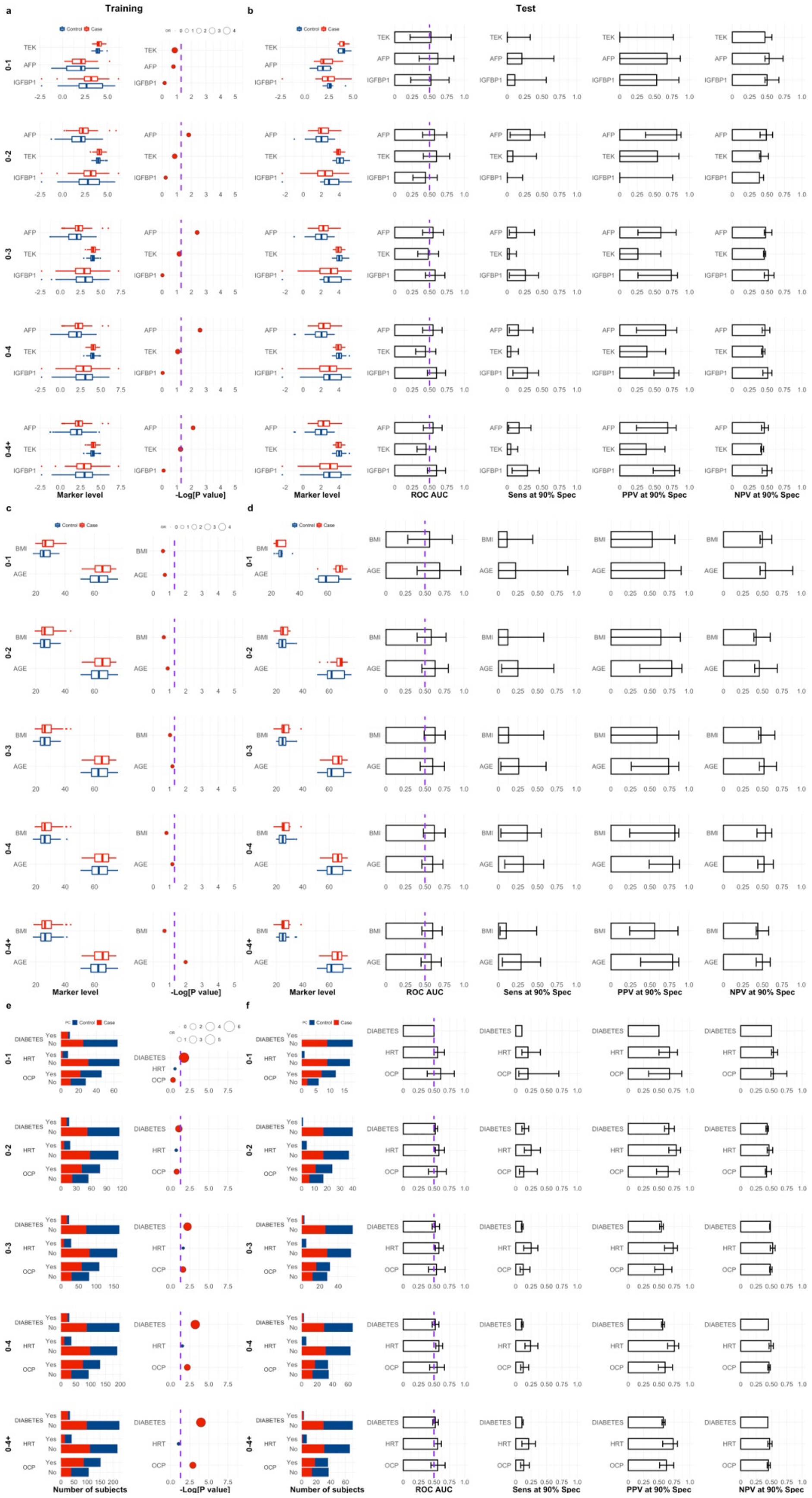

Training
Test
a
b
c
d
e
f
Control
Case
TEK
AFP
IGFBP1
BMI
AGE
DIABETES
HRT
OCP
Yes
No
0-1
0-2
0-3
0-4
0-4+
Marker level
-Log[P value]
ROC AUC
Sens at 90% Spec
PPV at 90% Spec
NPV at 90% Spec
Number of subjects

Supplementary Figure 4 Performance with the Bayesian Model Averaging (BMA) meta-learner for a subset of UKCTOCS participants for which AFP, TEK and IGFBP1 was also measured. a Receiver Operating curve (ROC) Area Under the Curve (AUC) across training folds for the enhanced Joined Time Group 2 Layer (JTG2L) model**.** Red error bars and dot represent the 95% Confidence Interval (CI) for the mean and the mean, generated by bootstrapping with the *boot* R package (version 1.3-25)**. b** ROC AUC in the test set with the respective model developed in the training set**.** Error bars in test set figures correspond to 95% CI for AUCs, calculated with stratified bootstrapping 2000 times. For a the number of independent samples was n=74 (0-1), n=131 (0-2), n=189 (0-3), n=225 (0-4), n=258 (0-4+). For b n=18 (0-1), n=43 (0-2), n=62 (0-3), n=72 (0-4), n=76 (0-4+). See Supplementary Figure 3, Supplementary tables 5, 6 and 7 for further details on case and control samples. Different shades of blue correspond to different time-groups. See Statistical Analysis in Methods (main text) for further details.

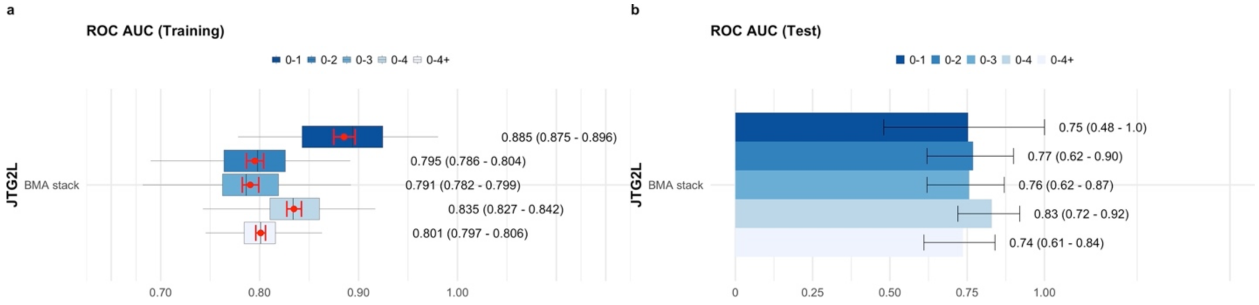

Supplementary Figure 5 Sensitivity, positive and negative predictive value with the Bayesian Model Averaging (BMA) meta-learner for a subset of UKCTOCS participants for which AFP, TEK and IGFBP1 was also measured. a Sensitivity (Sens), **b** Positive predictive value (PPV) and **c** Negative predictive value (NPV) at 90% Specificity (Spec) for the enhanced Joined Time Group 2 Layer (JTG2L) model. Error bars correspond to 95% Confidence Intervals (CI), calculated with stratified bootstrapping 2000 times. For a, the number of independent samples was n=74 (0-1), n=131 (0-2), n=189 (0-3), n=225 (0-4), n=258 (0-4+). For b n=18 (0-1), n=43 (0-2), n=62 (0-3), n=72 (0-4), n=76 (0-4+). See Supplementary Figure 3, Supplementary tables 5, 6 and 7 for further details on case and control samples. Different shades of blue correspond to different time-groups. See Statistical Analysis in Methods (main text) for further details.

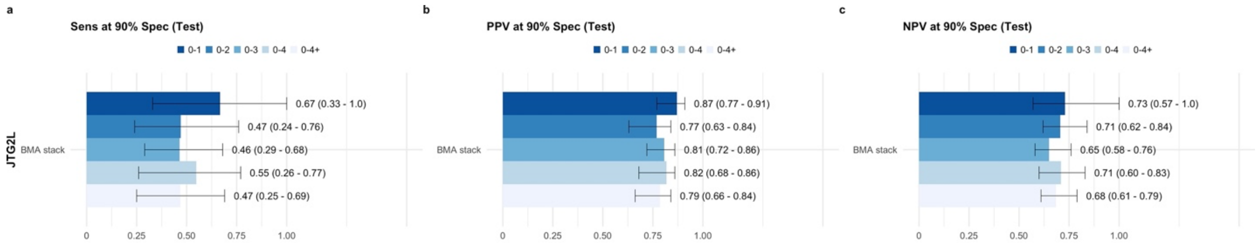

Supplementary Figure 6 Characteristic proportions for Diabetes, Hormonal Replacement Therapy (HRT) and Oral Contraceptive Pill (OCP), and feature ranks in the training set per joined time group according to a logistic regression model with a bias reduction method for a subset of UKCTOCS participants for which LRG1, PIGR, REG3A, F12, AGR2, IL17RA, SERPINA1, THBS1 was measured. a, c and **e** Distribution and ranks of biomarkers by p values in the training set. The purple dashed line corresponds to -Log[0.05]. **b** Performance of single marker models in the samples belonging to the sub test set. OR stands for odds-ratio, and dot size is proportional to the calculated value. Red and blue OR points represent OR > 1 (favours PDAC status) and OR < 1 (favours Control status), respectively. P values were calculated according to a logistic regression model with a bias reduction methods. Performances were calculated with the single feature models developed in the training set. The Receiver Operating curve (ROC) Area Under the Curve (AUC) significance threshold is also represented by a purple dashed line at 0.5. Error bars in figures corresponding to the test set correspond to 95% Confidence Intervals (CI), calculated by stratified bootstrapping 2000 times. For a, c and e the number of independent samples was n=60 (0-1), n=83 (0-2), n=108 (0-3), n=128 (0-4), n=149 (0-4+). For b, d and f n=15 (0-1), n=30 (0-2), n=34 (0-3), n=43 (0-4), n=55 (0-4+). See Supplementary tables 8, 9 and 10 for further details on case and control samples. See Statistical Analysis in Methods (main text) for further details.

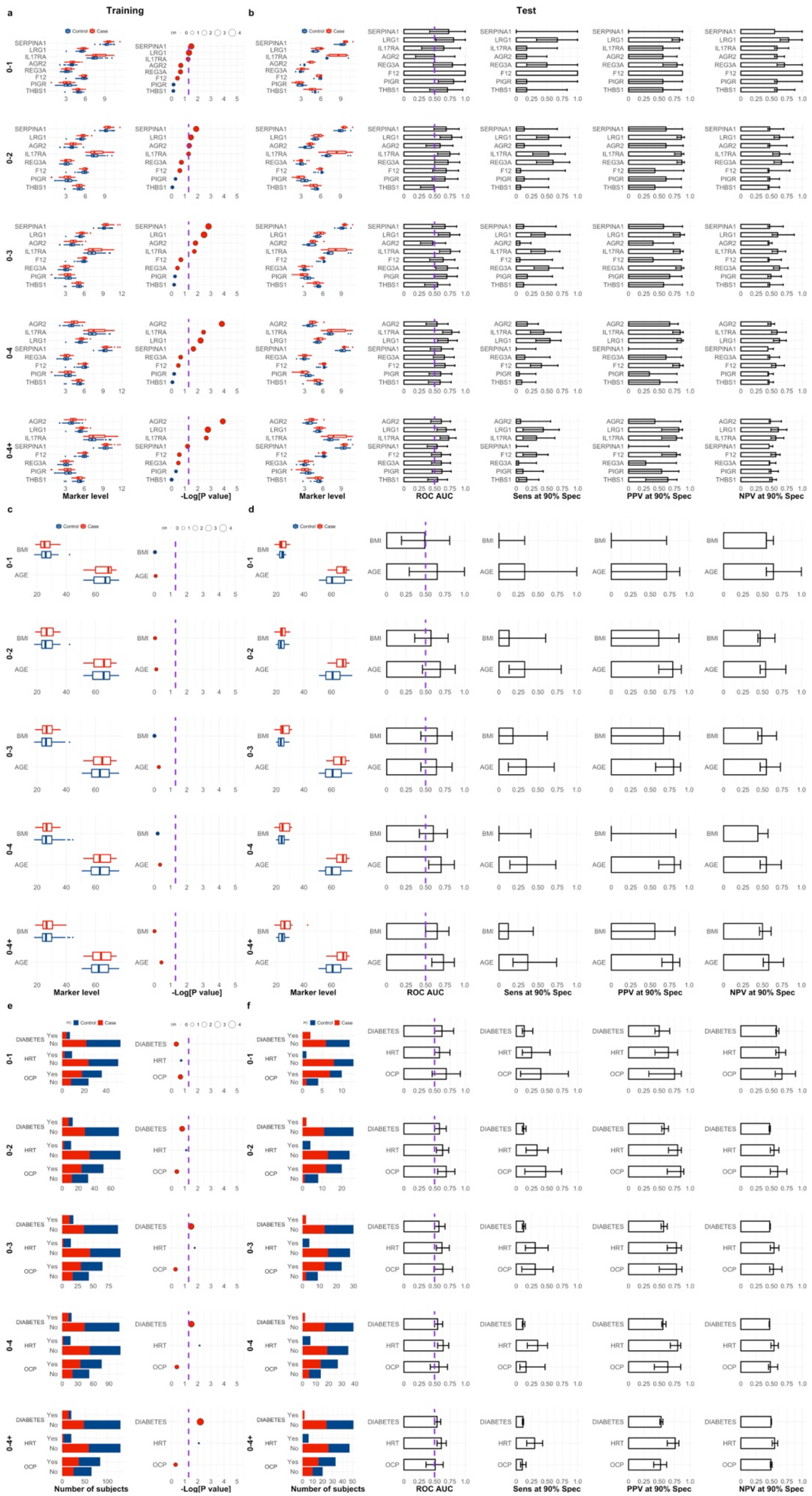

Supplementary Figure 7 Performance with the Bayesian Model Averaging (BMA) meta-learner for a subset of UKCTOCS participants for which LRG1, PIGR, REG3A, F12, AGR2, IL17RA, SERPINA1 and THBS1 were also measured. a Receiver Operating Curve (ROC) Area Under the Curve (AUC) across training folds for the enhanced Joined Time Group 2 Layer (JTG2L) model**.** Red error bars and dot represent the 95% Confidence Intervals (CI) for the mean and the mean, generated by bootstrapping with the *boot* R package (version 1.3-25)**. b** ROC AUC in the test set with the respective model developed in the training set**.** Error bars in test set figures correspond to 95% CIs, calculated with stratified bootstrapping 2000 times. For a, the number of independent samples was n=60 (0-1), n=83 (0-2), n=108 (0-3), n=128 (0-4), n=149 (0-4+). For b, n=15 (0-1), n=30 (0-2), n=34 (0-3), n=43 (0-4), n=55 (0-4+). See Supplementary Figure 6, Supplementary tables 8, 9 and 10 for further details on case and control samples. Different shades of blue correspond to different time-groups. See Statistical Analysis in Methods (main text) for further details.

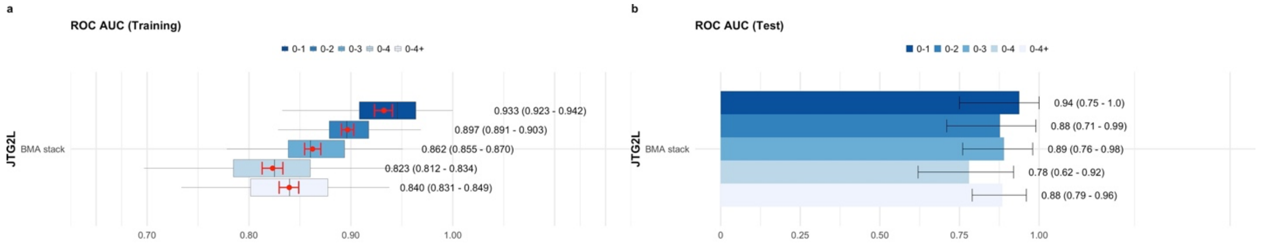

Supplementary Figure 8 Sensitivity, positive and negative predictive value with the Bayesian Model Averaging (BMA) meta-learner for a subset of UKCTOCS participants for which LRG1, PIGR, REG3A, F12, AGR2, IL17RA, SERPINA1 and THBS1 were also measured. a Sensitivity (Sens), **b** Positive predictive value (PPV) and **c** Negative predictive value (NPV) at 90% Specificity (Spec) for the enhanced JTG2L model. Error bars correspond to 95% Confidence Intervals (CI), calculated with stratified bootstrapping 2000 times. For a, b and c, n=15 (0-1), n=30 (0-2), n=34 (0-3), n=43 (0-4), n=55 (0-4+). See Supplementary Figure 6, Supplementary tables 8, 9 and 10 for further details on case and control samples. Different shades of blue correspond to different time-groups. See Statistical Analysis in Methods (main text) for further details.

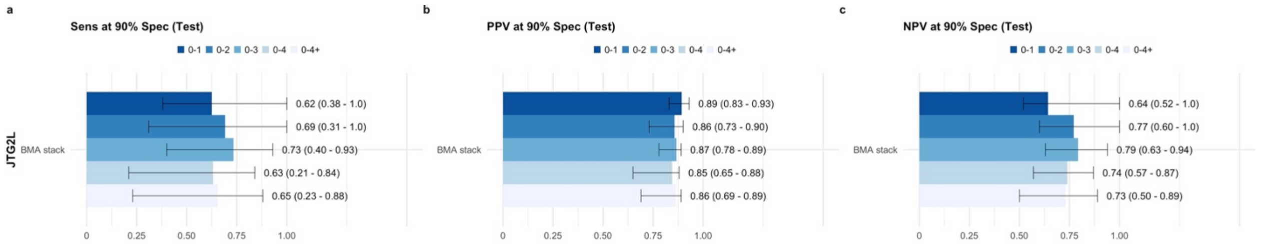

Supplementary Figure 9 Signatures for each joined time-group for the models presented in Supplementary Figures 4 and 7. a Scaled feature importance across base-learners for the pancreatic ductal adenocarcinoma (PDAC) signature developed in a subset of UKCTOCS participants for which AFP, TEK and IGFBP1 were also measured. **b** Scaled feature importance across base-learners for the PDAC signature developed for a subset of UKCTOCS participants for which LRG1, PIGR, REG3A, F12, AGR2, IL17RA, SERPINA1 and THBS1 were also measured. Feature importance was calculated with a model-agnostic method based on a simple feature importance ranking measure, implemented in the R package *vip*. Different shades of blue correspond to different time-groups. See Statistical Analysis in Methods (main text) for further details.

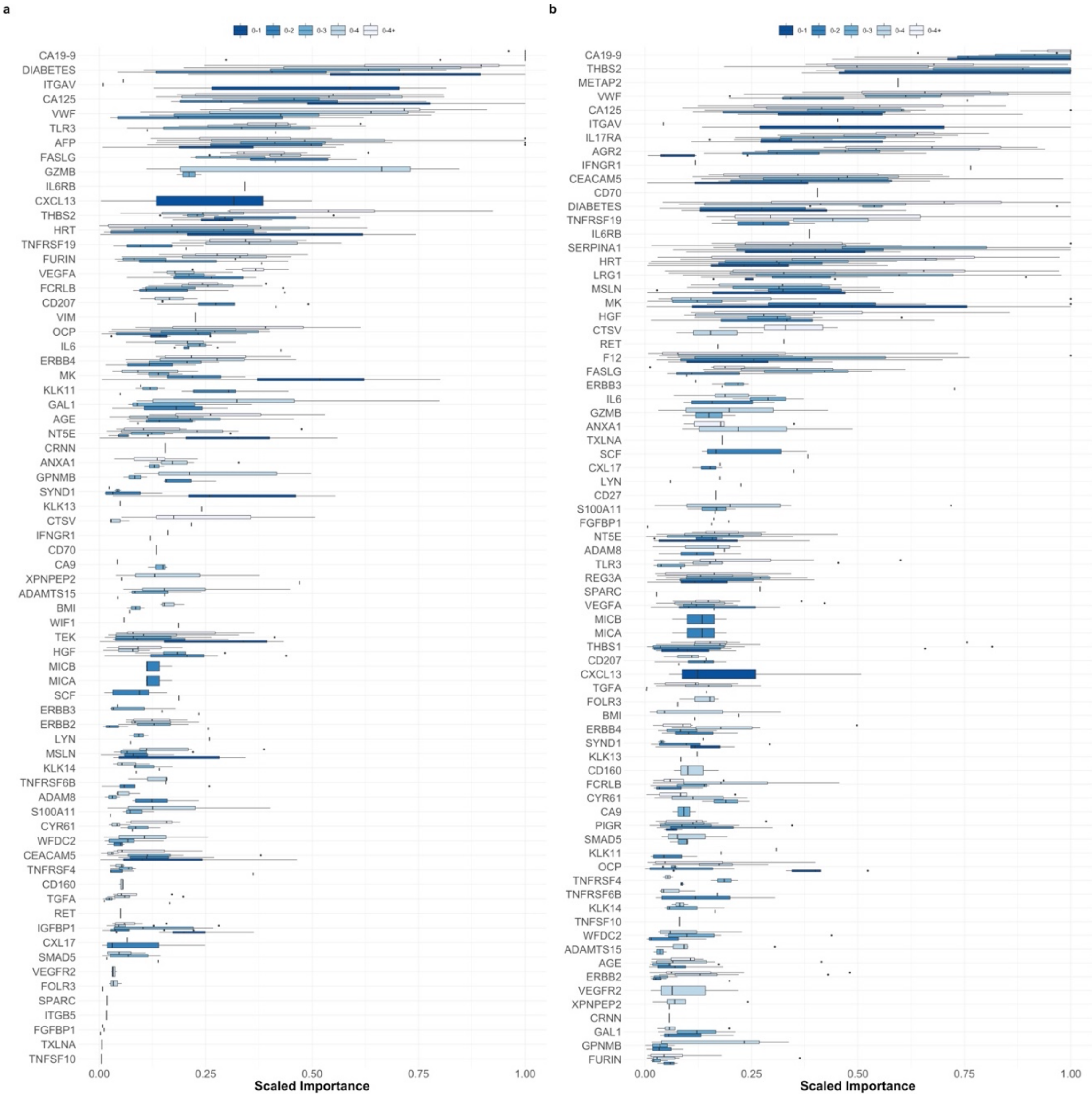

Supplementary Figure 10 Distributions for state-of-the-art markers and clinical covariates in the training and test sets, per single time-groups. a Marker distributions and p-values for CA19-9, MUC16, CEACAM5, THBS2, HGF and CEACAM1, in the training set. P values were calculated according to a logistic regression model with a bias reduction method. Purple dashed lines correspond to -Log [0.05]. **b** Marker distribution, Receiver Operating Curve (ROC) Area Under the Curve (AUC), Sensitivity (Sens), Positive Predictive Value (PPV) and Negative Predictive Value (NPV) at 90% Specificity (Spec) for the same biomarkers, but in the test set. Performances were determined with the respective unidimensional marker model developed in the training set. Error bars in test set figures correspond to 95% Confidence Intervals (CI), calculated with stratified bootstrapping 2000 times. **c** Marker values and respective p-values for AGE and Body Mass Index (BMI), in the training set. **d** Similar to b but for AGE and BMI. **e** and **f** Same as above but for Diabetes, Hormone Replacement Therapy (HRT) and Oral Contraceptive Pill (OCP) use. In b, d, f the ROC AUC significance threshold is also represented by a purple dashed line at 0.5. OR stands for odds-ratio, with dot size proportional to calculated values. Red and blue OR points represent OR > 1 (favours PDAC status) and OR < 1 (favours Control status), respectively. For a, c and e the number of independent samples was n=107 (0-1), n=73 (1-2), n=72 (2-3), n=57 (3-4) and n=54 (4+). For b, d and f the number of independent samples was n=26 (0-1), n=34 (1-2), n=22 (2-3), n=16 (3-4) and n=16 (4+). See Supplementary table 12 for further details on case and control samples. See Statistical Analysis in Methods (main text) for further details.

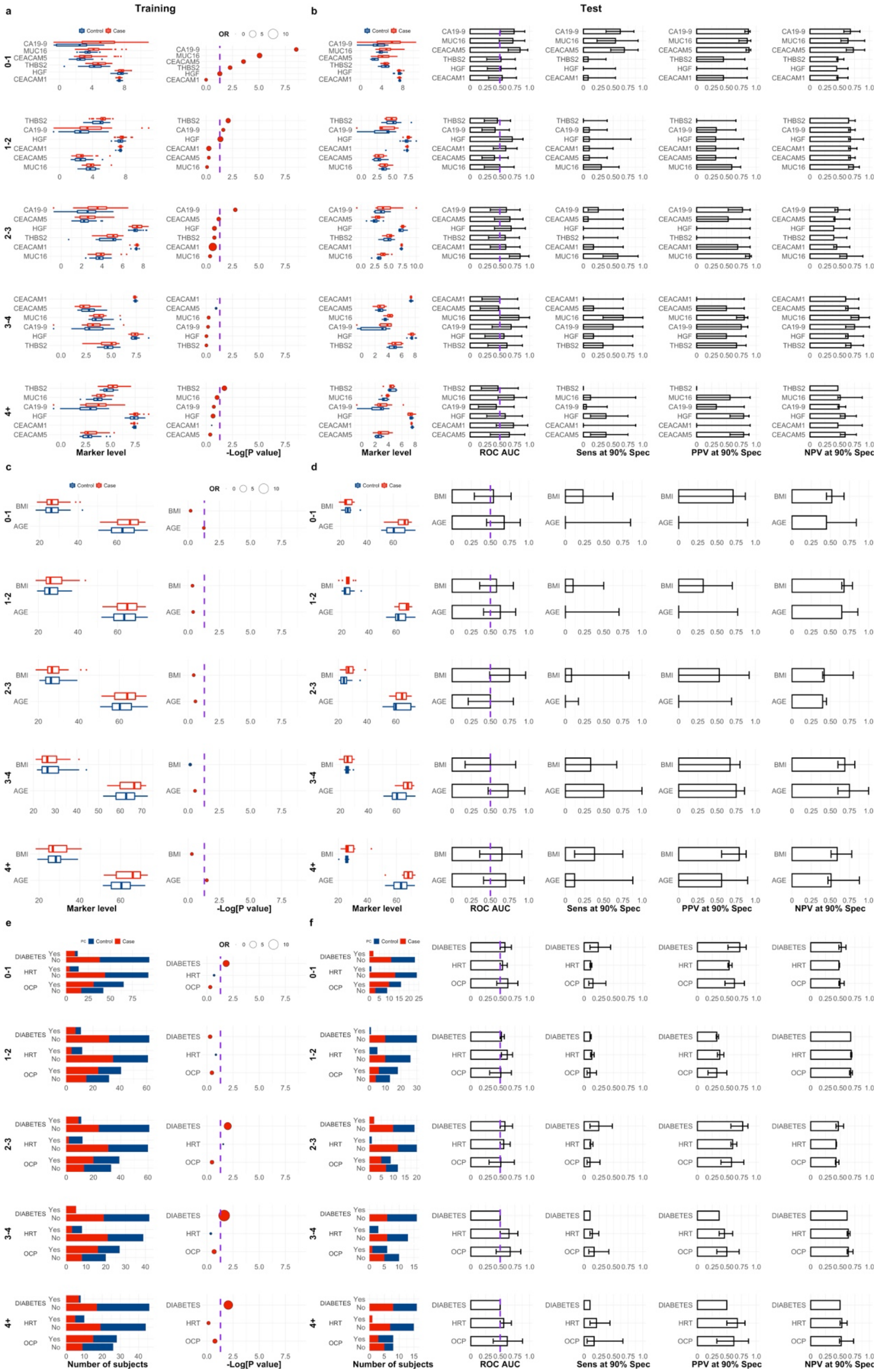

Training
Test
Marker level
-Log[P value]
ROC AUC
Sens at 90% Spec
PPV at 90% Spec
NPV at 90% Spec
Number of subjects

Supplementary Figure 11 Number of UKCTOCS participants according to pancreatic ductal adenocarcinoma (PDAC) status and speculated CA19-9 Lewis negative status (Le-). a and **b** Single time-groups. **c** and **d** Joined time-groups. P values were calculated according to a logistic regression model with a bias reduction method. Purple dashed line corresponds to -Log [0.05]. Receiver Operating Curve (ROC) Area Under the Curve (AUC) values were calculated with the single feature models developed in the training set. Error bars in test set figures correspond to 95% Confidence Intervals (CI), calculated with stratified bootstrapping 2000 times, for ROC AUC, and Sensitivity (Sens), Positive Predictive Value (PPV) and Negative Predictive Value (NPV) at 90% Specificity (Spec). The Le- character associated with each participant was not determined by sequencing but by the absence of CA19-9 in the sample, which makes it a speculative feature. For a, the number of independent samples was n=107 (0-1), n=73 (1-2), n=72 (2-3), n=57 (3-4) and n=54 (4+). For b the number of independent samples was n=34 (1-2), n=22 (2-3), n=16 (3-4) and n=16 (4+). For c n=180 (0-2), n=252 (0-3), n=309 (0-4) and n=363 (0-4+). For d n=60 (0-2), n=82 (0-3), n=98 (0-4) and n=114 (0-4+). See Supplementary Table 12 for further details on case and control samples. See Statistical Analysis in Methods (main text) for further details.

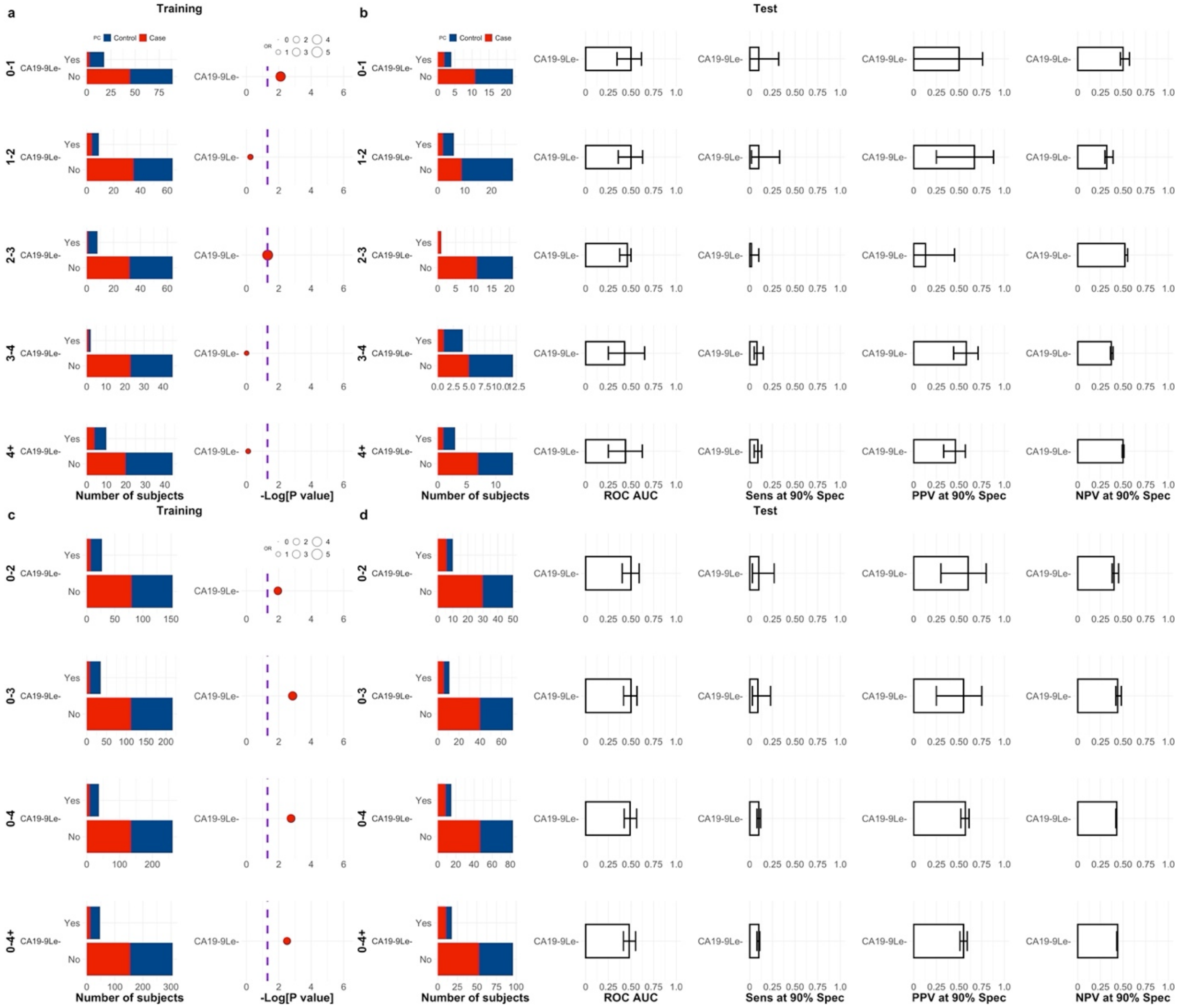

Supplementary Figure 12 Feature ranks in the training, time-group 0-1 years to diagnosis. a Distribution and ranks of biomarkers by p values in the training set. Purple dashed line corresponds to -Log[0.05]. **b** Receiver Operating Curve (ROC) Area Under the Curve (AUC), Sensitivity (Sens), Positive Predictive Value (PPV) and Negative Predictive Value (NPV) at 90% Specificity (Spec) performance of single marker models in the test set,. OR stands for odds-ratio, with dot size proportional to the calculated values. Red and blue OR points represent OR > 1 (favours pancreatic ductal adenocarcinoma (PDAC) case status) and OR < 1 (favours Control status), respectively. P values were calculated according to a logistic regression model with a bias reduction method. Performances were calculated with the single feature models developed in the training set. The ROC AUC significance threshold is also represented by a purple dashed line at 0.5. Error bars in figures corresponding to the test set correspond to 95% Confidence Intervals (CI), calculated by stratified bootstrapping 2000 times. For a and b, the number of independent samples was n=107 (0-1) and n=26 (0-1), respectively. See Supplementary table 12 for further details on case and control samples. See Statistical Analysis in Methods (main text) for further details.

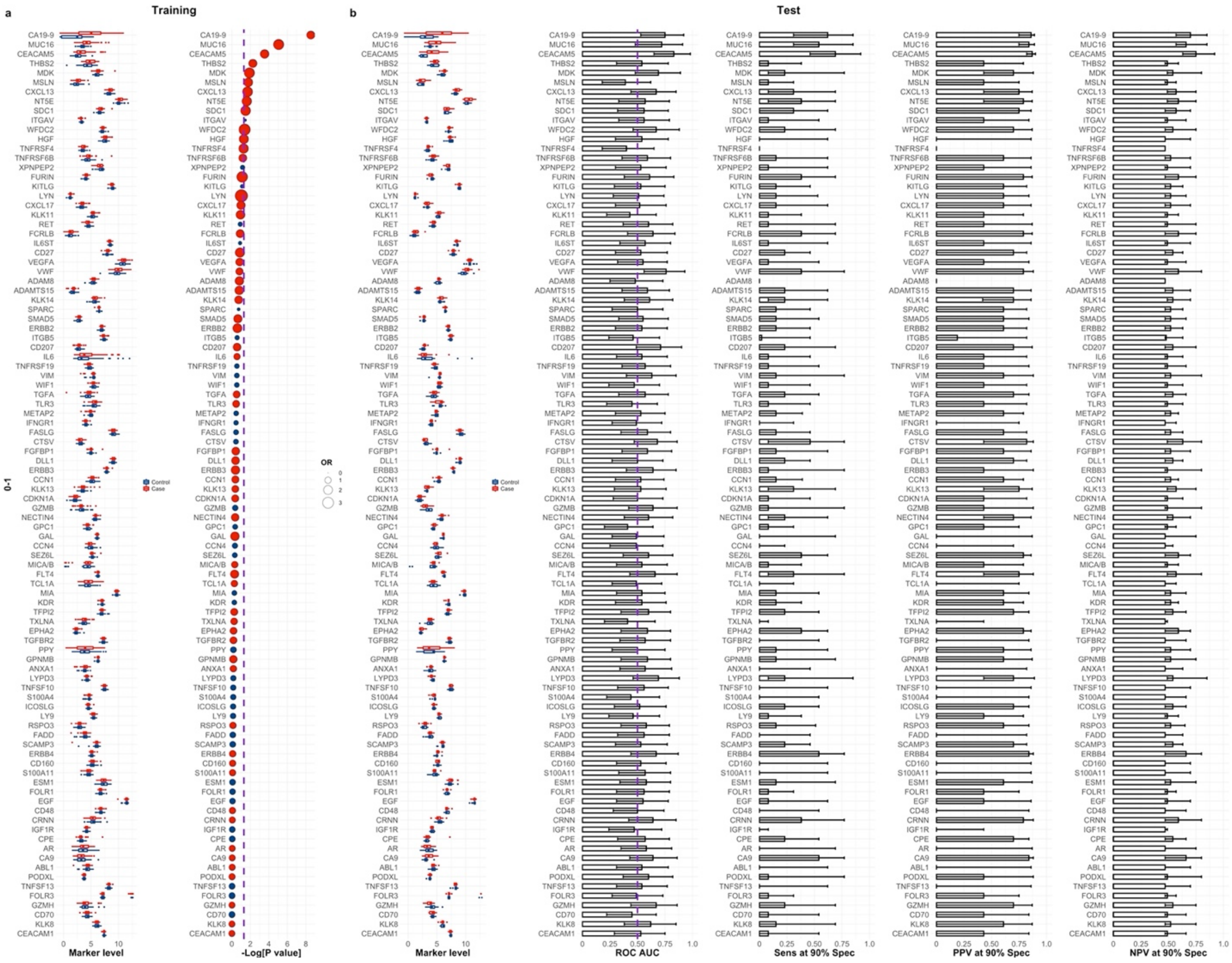

Supplementary Figure 13 Feature ranks in the training, time-group 1-2 years to diagnosis. a Distribution and ranks of biomarkers by p values in the training set. Purple dashed line corresponds to -Log[0.05]. **b** Receiver Operating Curve (ROC) Area Under the Curve (AUC), Sensitivity (Sens), Positive Predictive Value (PPV) and Negative Predictive Value (NPV) at 90% Specificity (Spec) performance of single marker models in the test set,. OR stands for odds-ratio, with dot size proportional to the calculated values. Red and blue OR points represent OR > 1 (favours pancreatic ductal adenocarcinoma (PDAC) case status) and OR < 1 (favours Control status), respectively. P values were calculated according to a logistic regression model with a bias reduction method. Performances were calculated with the single feature models developed in the training set. The ROC AUC significance threshold is also represented by a purple dashed line at 0.5. Error bars in figures corresponding to the test set correspond to 95% Confidence Intervals (CI), calculated by stratified bootstrapping 2000 times. For a and b the number of independent samples was n=73 (1-2) and n=34 (1-2), respectively. See Supplementary table 12 for further details on case and control samples. See Statistical Analysis in Methods (main text) for further details.

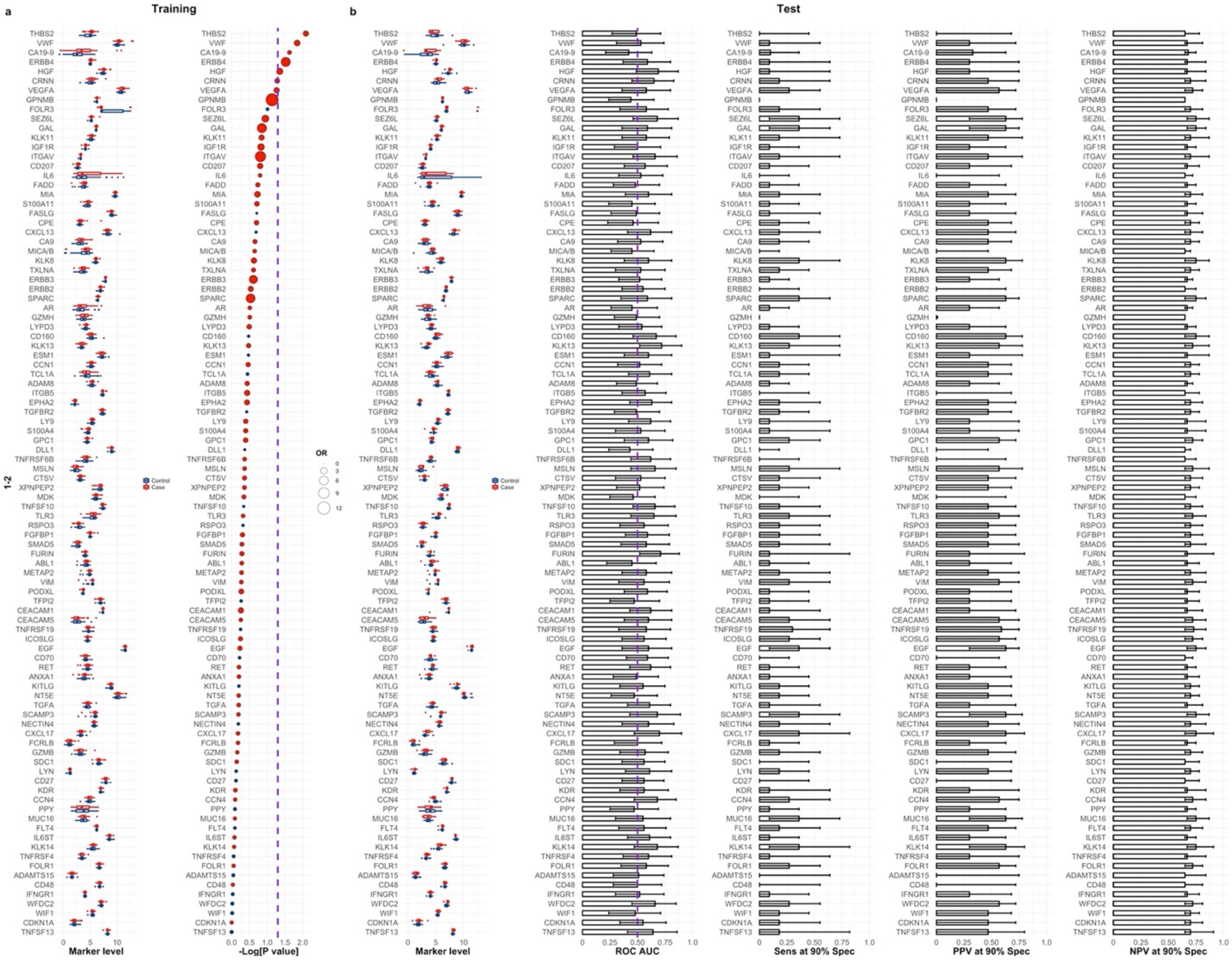

Supplementary Figure 14 Feature ranks in the training, time-group 2-3 years to diagnosis. a Distribution and ranks of biomarkers by p values in the training set. Purple dashed line corresponds to -Log[0.05]. **b** Receiver Operating Curve (ROC) Area Under the Curve (AUC), Sensitivity (Sens), Positive Predictive Value (PPV) and Negative Predictive Value (NPV) at 90% Specificity (Spec) performance of single marker models in the test set,. OR stands for odds-ratio, with dot size proportional to the calculated values. Red and blue OR points represent OR > 1 (favours pancreatic ductal adenocarcinoma (PDAC) case status) and OR < 1 (favours Control status), respectively. P values were calculated according to a logistic regression model with a bias reduction method. Performances were calculated with the single feature models developed in the training set. The ROC AUC significance threshold is also represented by a purple dashed line at 0.5. Error bars in figures corresponding to the test set correspond to 95% Confidence Intervals (CI), calculated by stratified bootstrapping 2000 times. For a and b the number of independent samples was n=72 (2-3) and n=22 (2-3), respectively. See Supplementary table 12 for further details on case and control samples. See Statistical Analysis in Methods (main text) for further details.

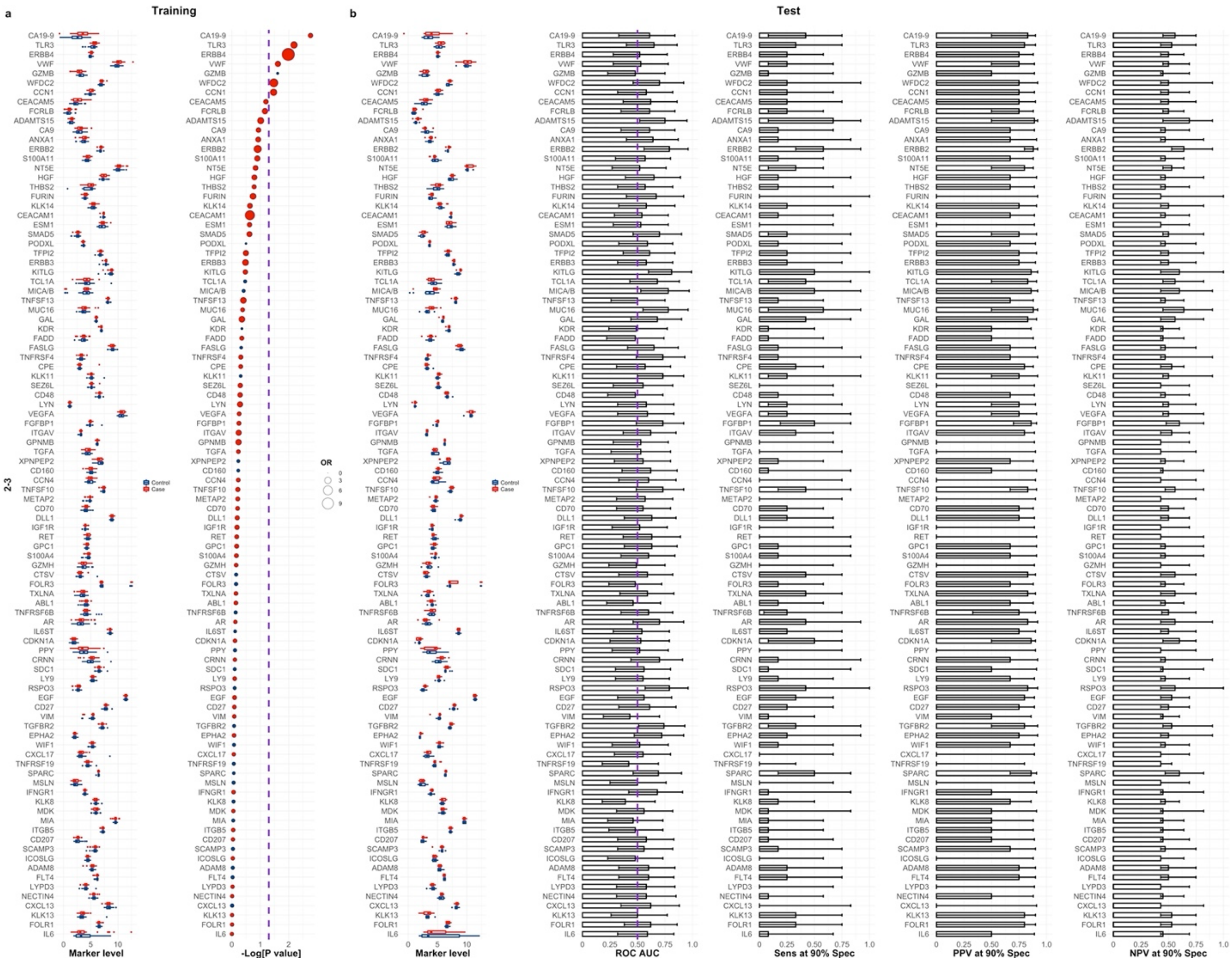

Supplementary Figure 15 Feature ranks in the training, time-group 3-4 years to diagnosis. a Distribution and ranks of biomarkers by p values in the training set. Purple dashed line corresponds to -Log[0.05]. **b** Receiver Operating Curve (ROC) Area Under the Curve (AUC), Sensitivity (Sens), Positive Predictive Value (PPV) and Negative Predictive Value (NPV) at 90% Specificity (Spec) performance of single marker models in the test set. OR stands for odds-ratio, with dot size proportional to the calculated values. Red and blue OR points represent OR > 1 (favours pancreatic ductal adenocarcinoma (PDAC) case status) and OR < 1 (favours Control status), respectively. P values were calculated according to a logistic regression model with a bias reduction method. Performances were calculated with the single feature models developed in the training set. The ROC AUC significance threshold is also represented by a purple dashed line at 0.5. Error bars in figures corresponding to the test set correspond to 95% Confidence Intervals (CI), calculated by stratified bootstrapping 2000 times. For a and b, the number of independent samples was n=57 (3-4) and n=16 (3-4), respectively. See Supplementary table 12 for further details on case and control samples. See Statistical Analysis in Methods (main text) for further details.

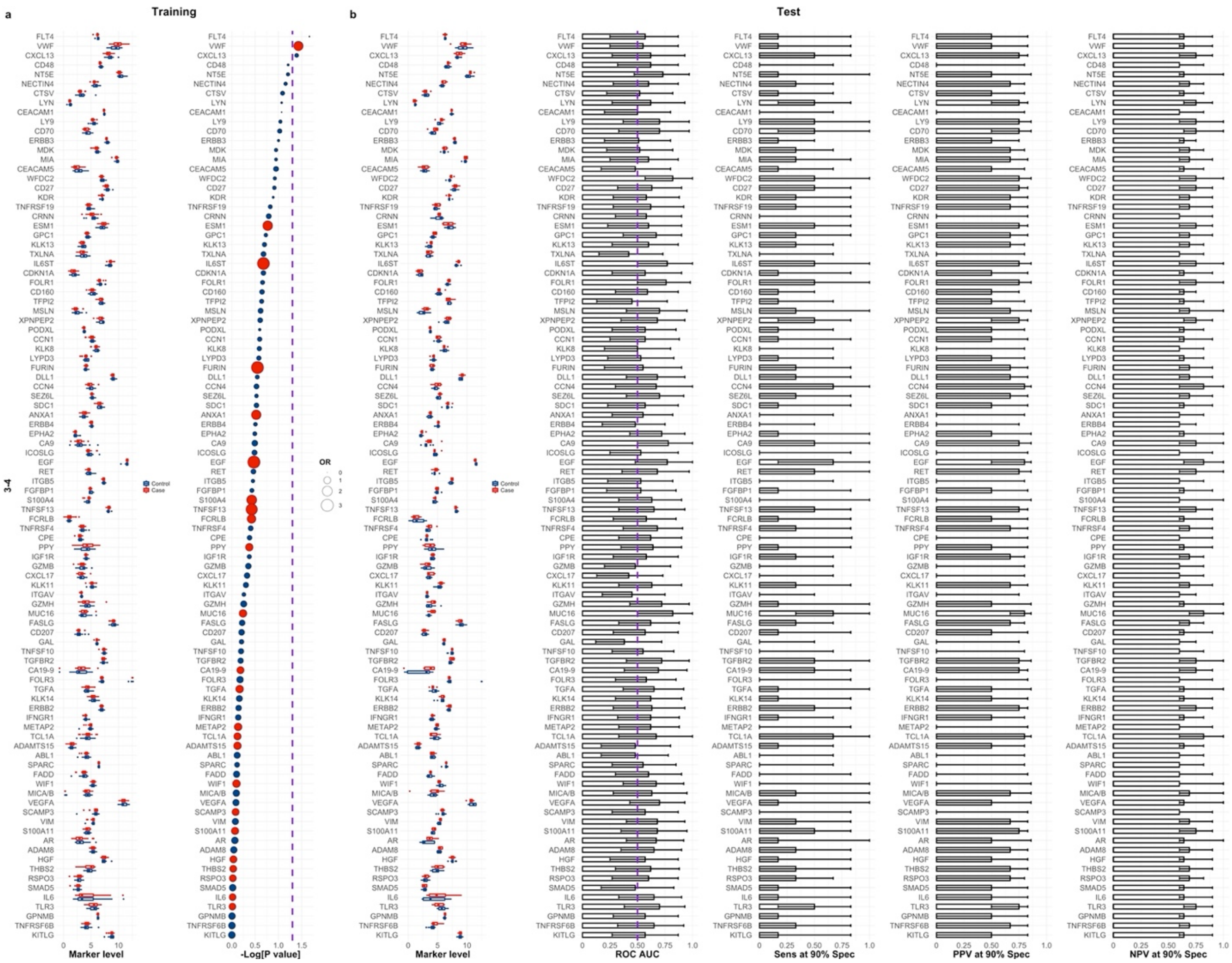

Supplementary Figure 16 Feature ranks in the training, time-group 4+ years to diagnosis. a Distribution and ranks of biomarkers by p values in the training set. Purple dashed line corresponds to -Log [0.05]. **b** Receiver Operating Curve (ROC) Area Under the Curve (AUC), Sensitivity (Sens), Positive Predictive Value (PPV) and Negative Predictive Value (NPV) at 90% Specificity (Spec) performance of single marker models in the test set. OR stands for odds-ratio, with dot size proportional to the calculated values. Red and blue OR points represent OR > 1 (favours pancreatic ductal adenocarcinoma (PDAC) case status) and OR < 1 (favours Control status), respectively. P values were calculated according to a logistic regression model with a bias reduction method. Performances were calculated with the single feature models developed in the training set. The ROC AUC significance threshold is also represented by a purple dashed line at 0.5. Error bars in figures corresponding to the test set correspond to 95% Confidence Intervals (CI), calculated by stratified bootstrapping 2000 times. For a and b, the number of independent samples was n=54 (4+) and n=16 (4+), respectively. See Supplementary table 12 for further details on case and control samples. See Statistical Analysis in Methods (main text) for further details.

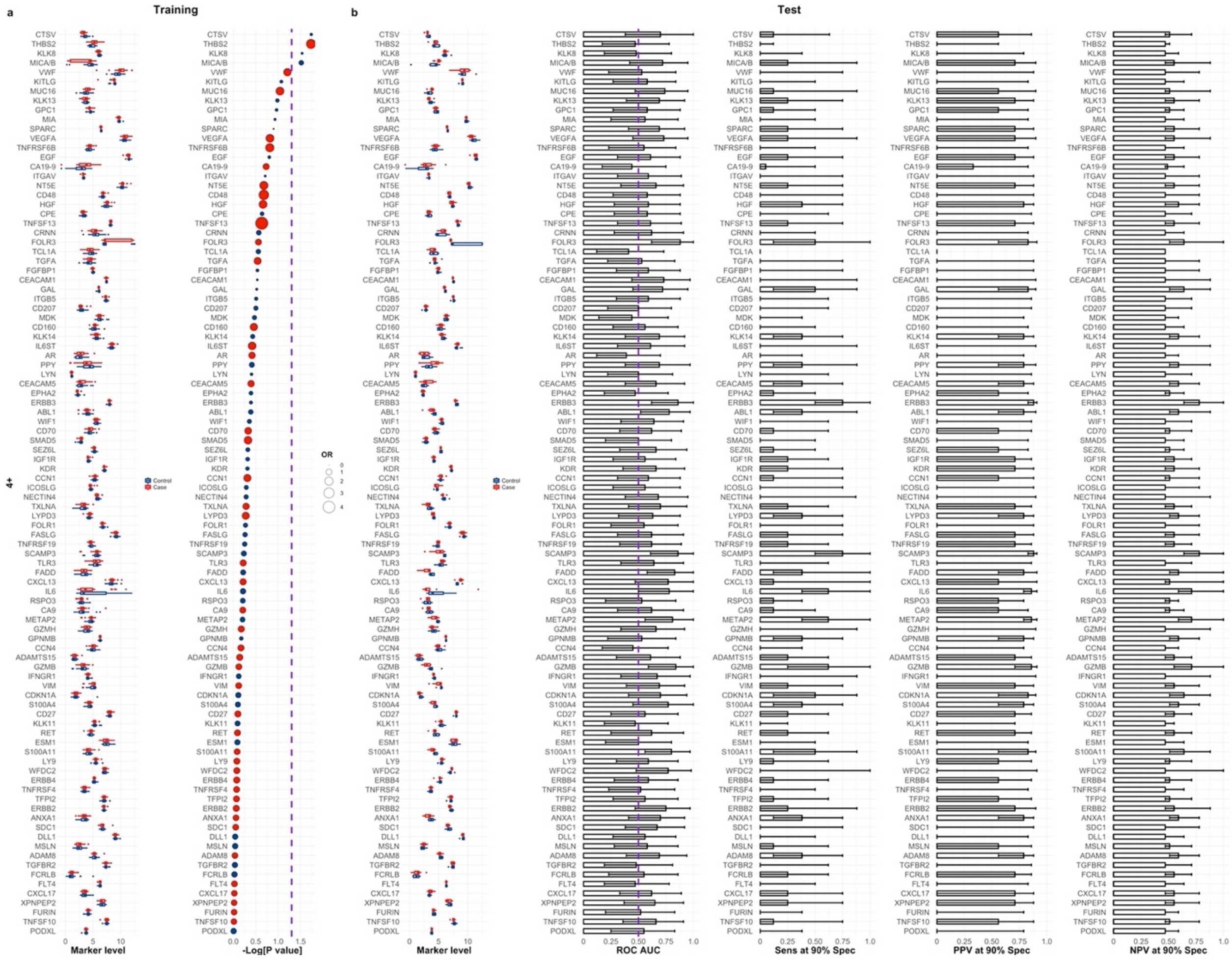

Supplementary Figure 17 Feature ranks in the training, time-group 0-2 years to diagnosis. a Distribution and ranks of biomarkers by p values in the training set. Purple dashed line corresponds to -Log [0.05]. **b** Receiver Operating Curve (ROC) Area Under the Curve (AUC), Sensitivity (Sens), Positive Predictive Value (PPV) and Negative Predictive Value (NPV) at 90% Specificity (Spec) performance of single marker models in the test set. OR stands for odds-ratio, with dot size proportional to the calculated values. Red and blue OR points represent OR > 1 (favours pancreatic ductal adenocarcinoma (PDAC) case status) and OR < 1 (favours Control status), respectively. P values were calculated according to a logistic regression model with a bias reduction method. Performances were calculated with the single feature models developed in the training set. The ROC AUC significance threshold is also represented by a purple dashed line at 0.5. Error bars in figures corresponding to the test set correspond to 95% Confidence Intervals (CI), calculated by stratified bootstrapping 2000 times. For a and b the number of samples was n=180 (0-2) and n=60 (0-2), respectively. See Supplementary table 12 for further details on case and control samples. See Statistical Analysis in Methods (main text) for further details.

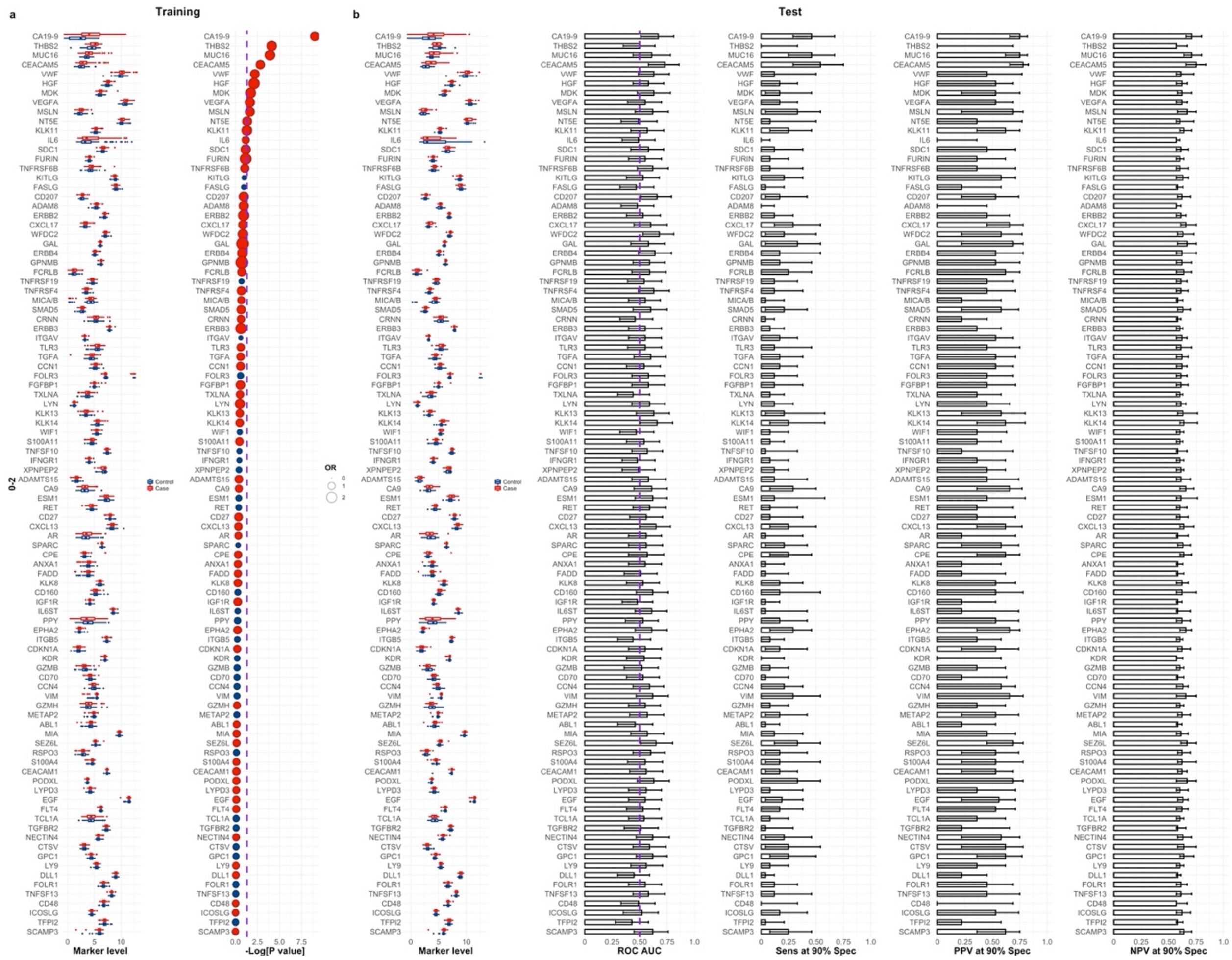

Supplementary Figure 18 Feature ranks in the training, time-group 0-3 years to diagnosis. a Distribution and ranks of biomarkers by p values in the training set. Purple dashed line corresponds to -Log [0.05]. **b** Receiver Operating Curve (ROC) Area Under the Curve (AUC), Sensitivity (Sens), Positive Predictive Value (PPV) and Negative Predictive Value (NPV) at 90% Specificity (Spec) performance of single marker models in the test set. OR stands for odds-ratio, with dot size proportional to the calculated values. Red and blue OR points represent OR > 1 (favours pancreatic ductal adenocarcinoma (PDAC) case status) and OR < 1 (favours Control status), respectively. P values were calculated according to a logistic regression model with a bias reduction method. Performances were calculated with the single feature models developed in the training set. The ROC AUC significance threshold is also represented by a purple dashed line at 0.5. Error bars in figures corresponding to the test set correspond to 95% Confidence Intervals (CI), calculated by stratified bootstrapping 2000 times. For a and b, the number of samples was n=252 (0-3) and n=82 (0-3), respectively. See Supplementary table 12 for further details on case and control samples. See Statistical Analysis in Methods (main text) for further details.

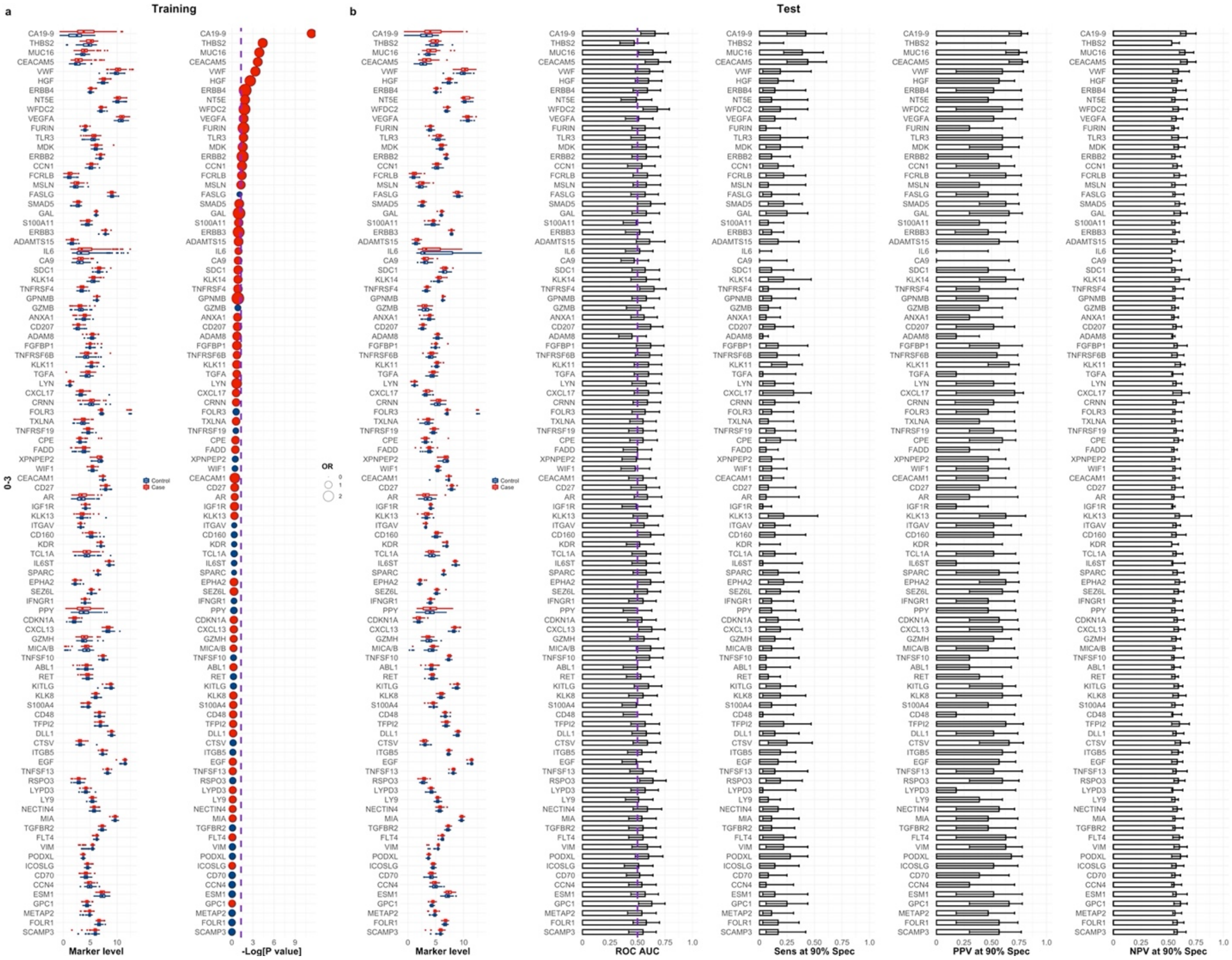

Supplementary Figure 19 Feature ranks in the training, time-group 0-4 years to diagnosis. a Distribution and ranks of biomarkers by p values in the training set. The purple dashed line corresponds to -Log [0.05]. **b** Receiver Operating Curve (ROC) Area Under the Curve (AUC), Sensitivity (Sens), Positive Predictive Value (PPV) and Negative Predictive Value (NPV) at 90% Specificity (Spec) performance of single marker models in the test set. OR stands for odds-ratio, with dot size proportional to the calculated values. Red and blue OR points represent OR > 1 (favours pancreatic ductal adenocarcinoma (PDAC) case status) and OR < 1 (favours Control status), respectively. P values were calculated according to a logistic regression model with a bias reduction method. Performances were calculated with the single feature models developed in the training set. The ROC AUC significance threshold is also represented by a purple dashed line at 0.5. Error bars in figures corresponding to the test set correspond to 95% Confidence Intervals (CI), calculated by stratified bootstrapping 2000 times. For a and b, the number of samples was n=309 (0-4) and n=98 (0-4), respectively. See Supplementary table 12 for further details on case and control samples. See Statistical Analysis in Methods (main text) for further details.

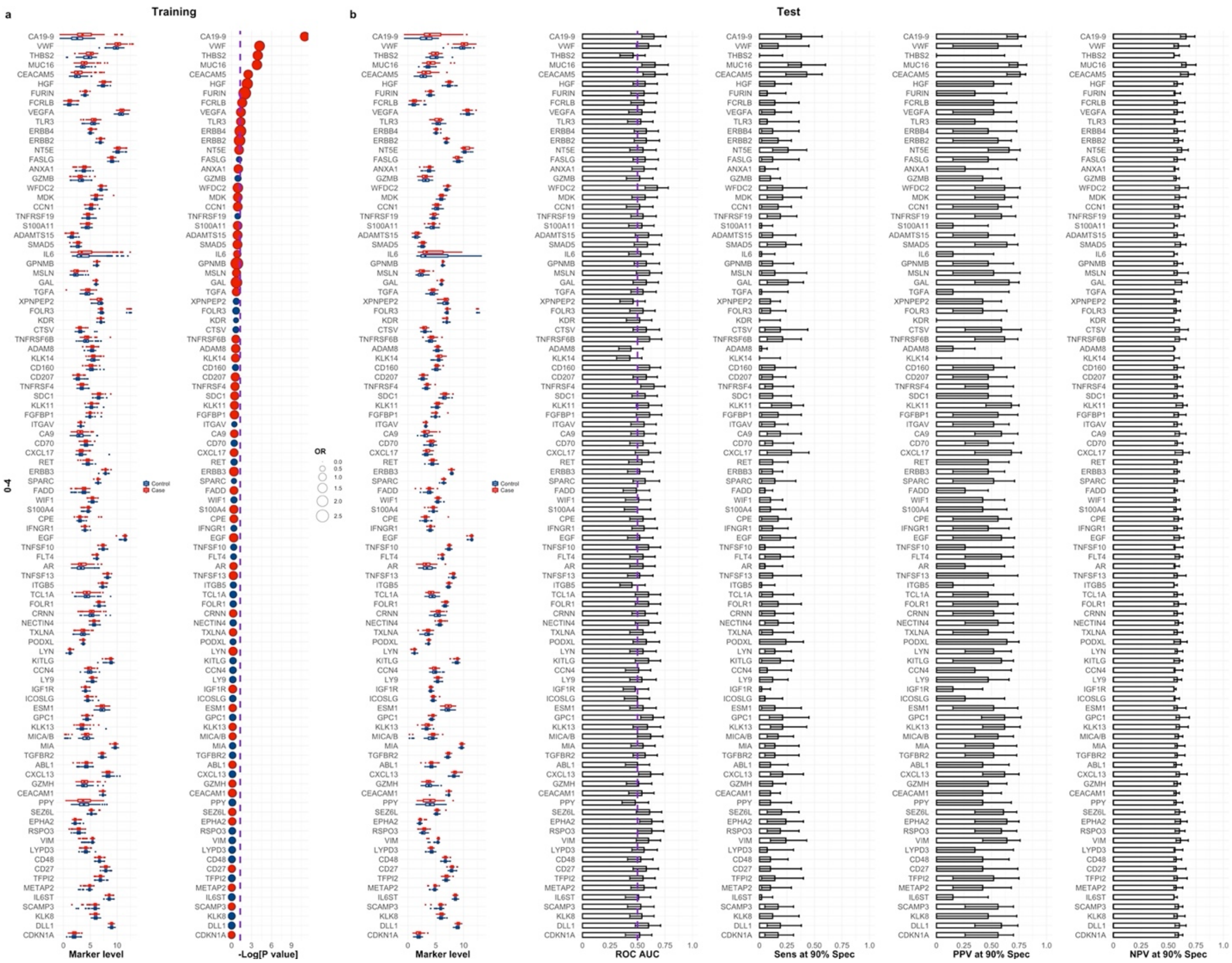

Supplementary Figure 20 Feature ranks in the training, time-group 0-4+ years to diagnosis. a Distribution and ranks of biomarkers by p values in the training set. The purple dashed line corresponds to -Log [0.05]. **b** Receiver Operating Curve (ROC) Area Under the Curve (AUC), Sensitivity (Sens), Positive Predictive Value (PPV) and Negative Predictive Value (NPV) at 90% Specificity (Spec) performance of single marker models in the test set. OR stands for odds-ratio, with dot size proportional to the calculated values. Red and blue OR points represent OR > 1 (favours pancreatic ductal adenocarcinoma (PDAC) case status) and OR < 1 (favours Control status), respectively. P values were calculated according to a logistic regression model with a bias reduction method. Performances were calculated with the single feature models developed in the training set. The ROC AUC significance threshold is also represented by a purple dashed line at 0.5. Error bars in figures corresponding to the test set correspond to 95% Confidence Intervals (CI), calculated by stratified bootstrapping 2000 times. For a and b the number of samples was n=363 (0-4+) and n=114 (0-4+), respectively. See Supplementary table 12 for further details on case and control samples. See Statistical Analysis in Methods (main text) for further details.

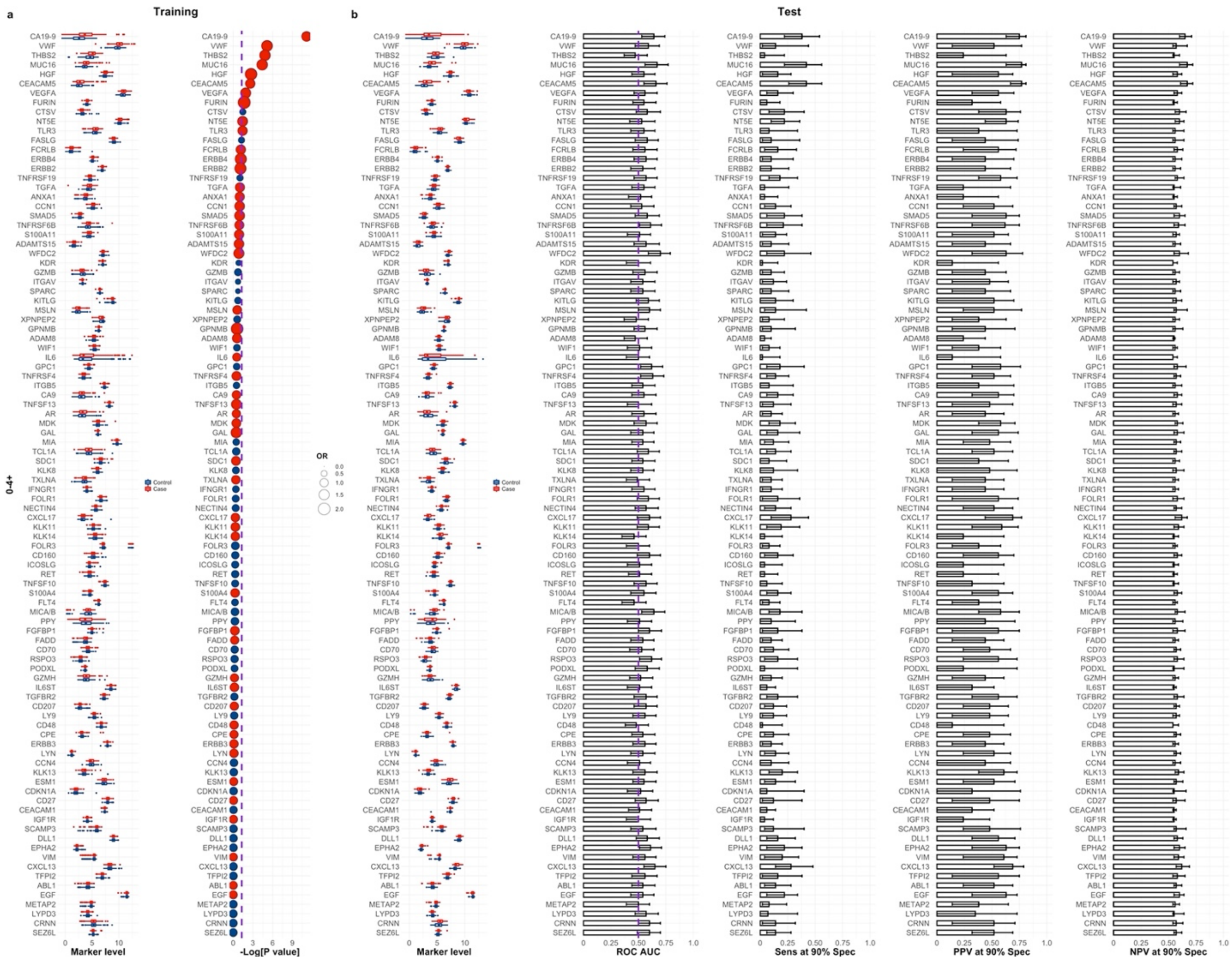

Supplementary Figure 21 Performances for state-of-the-art multi-marker and clinical covariate combinations in a logistic regression model, top models for joined time-groups (Test set). a Receiver Operating Curve (ROC) Area Under the Curve (AUC). **b** Sensitivity (Sens), **c** Positive predictive value (PPV) and **d** Negative predictive value (NPV) at 90% Specificity (Spec)**.** Values were calculated with the combinations of features developed in the training set. Models are ranked according to their performance in the training set with a 5 times 10-fold cross-validation resampling strategy. Error bars correspond to 95% Confidence Intervals (CI) for AUCs, determined by stratified bootstrapping 2000 times. For a, b, c and d the number of independent training samples was n=107 (0-1), n=180 (0-2), n=252 (0-3), n=309 (0-4) and n=363 (0-4+), and test samples n=26 (0-1), n=60 (0-2), n=82 (0-3), n=98 (0-4) and n=114 (0-4+). Different shades of blue correspond to different time-groups. See Supplementary table 12 for further details on case and control samples. See Statistical Analysis in Methods (main text) for further details.

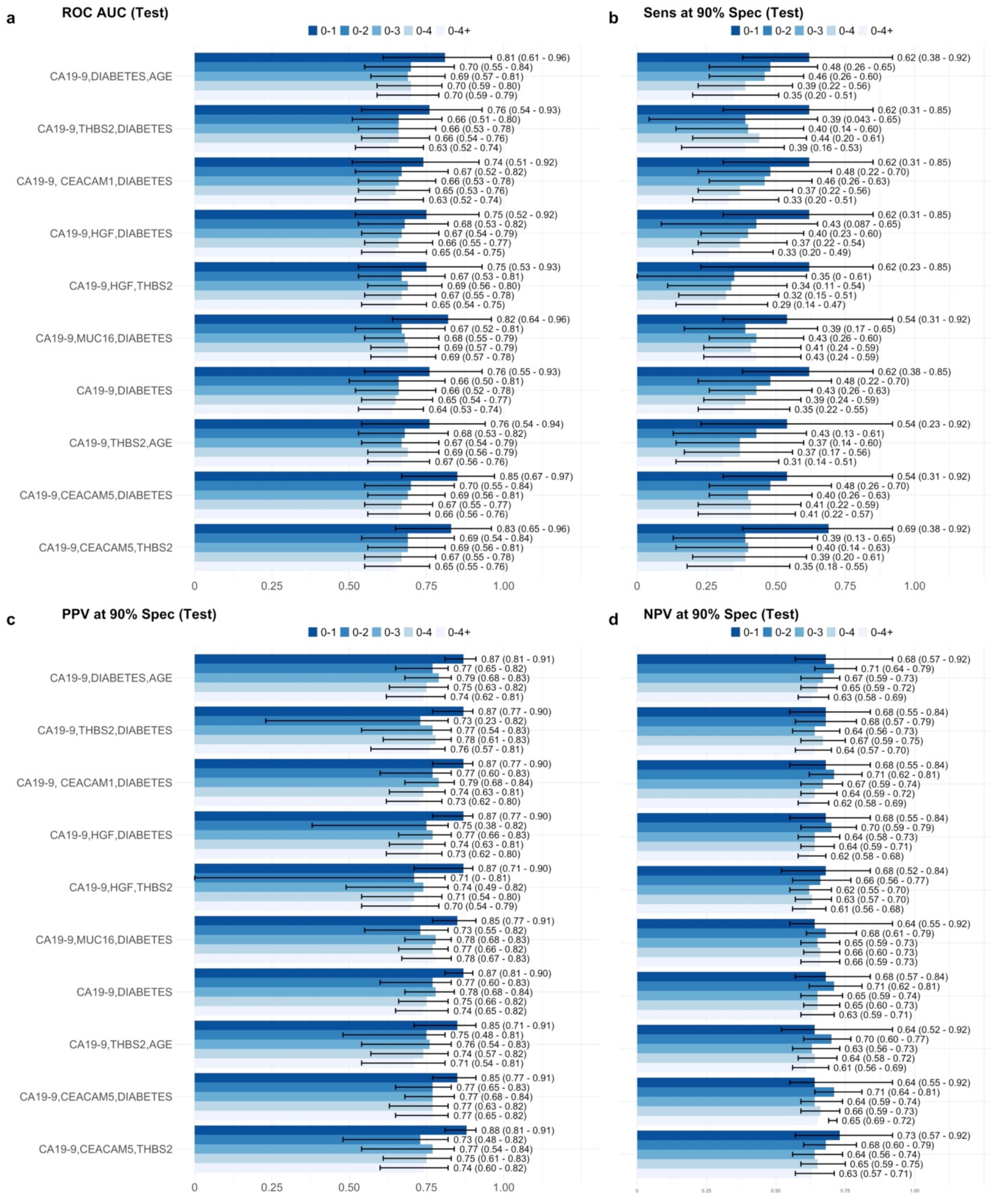
a
ROC AUC (Test)
b
Sens at 90% Spec (Test)
c
PPV at 90% Spec (Test)
d
NPV at 90% Spec (Test)
0-1
0-2
0-3
0-4
0-4+
CA19-9,DIABETES,AGE
CA19-9,THBS2,DIABETES
CA19-9, CEACAM1,DIABETES
CA19-9,HGF,DIABETES
CA19-9,HGF,THBS2
CA19-9,MUC16,DIABETES
CA19-9,DIABETES
CA19-9,THBS2,AGE
CA19-9,CEACAM5,DIABETES
CA19-9,CEACAM5,THBS2
0
0.25
0.50
0.75
1.00

Supplementary Figure 22 Performances for state-of-the-art multi-marker and clinical covariate combinations in a logistic regression model, top models for single time-groups (Test set). a Receiver Operating Curve (ROC) Area Under the Curve (AUC). **b** Sensitivity (Sens), **c** Positive predictive value (PPV) and **d** Negative predictive value (NPV) at 90% Specificity (Spec)**.** Values were calculated with the combinations of features developed in the training set. Models are ranked according to their performance in the training set with a 5 times 10-fold cross-validation resampling strategy. Error bars correspond to 95% Confidence Intervals (CI), determined by stratified bootstrapping 2000 times. For a, b, c and d the number of independent training samples was n=107 (0-1), n=73 (1-2), n=72 (2-3), n=57 (3-4) and n=54 (4+), and test samples n=34 (1-2), n=22 (2-3), n=16 (3-4) and n=16 (4+). Different shades of blue correspond to different time-groups. See Supplementary table 12 for further details on case and control samples. See Statistical Analysis in Methods (main text) for further details.

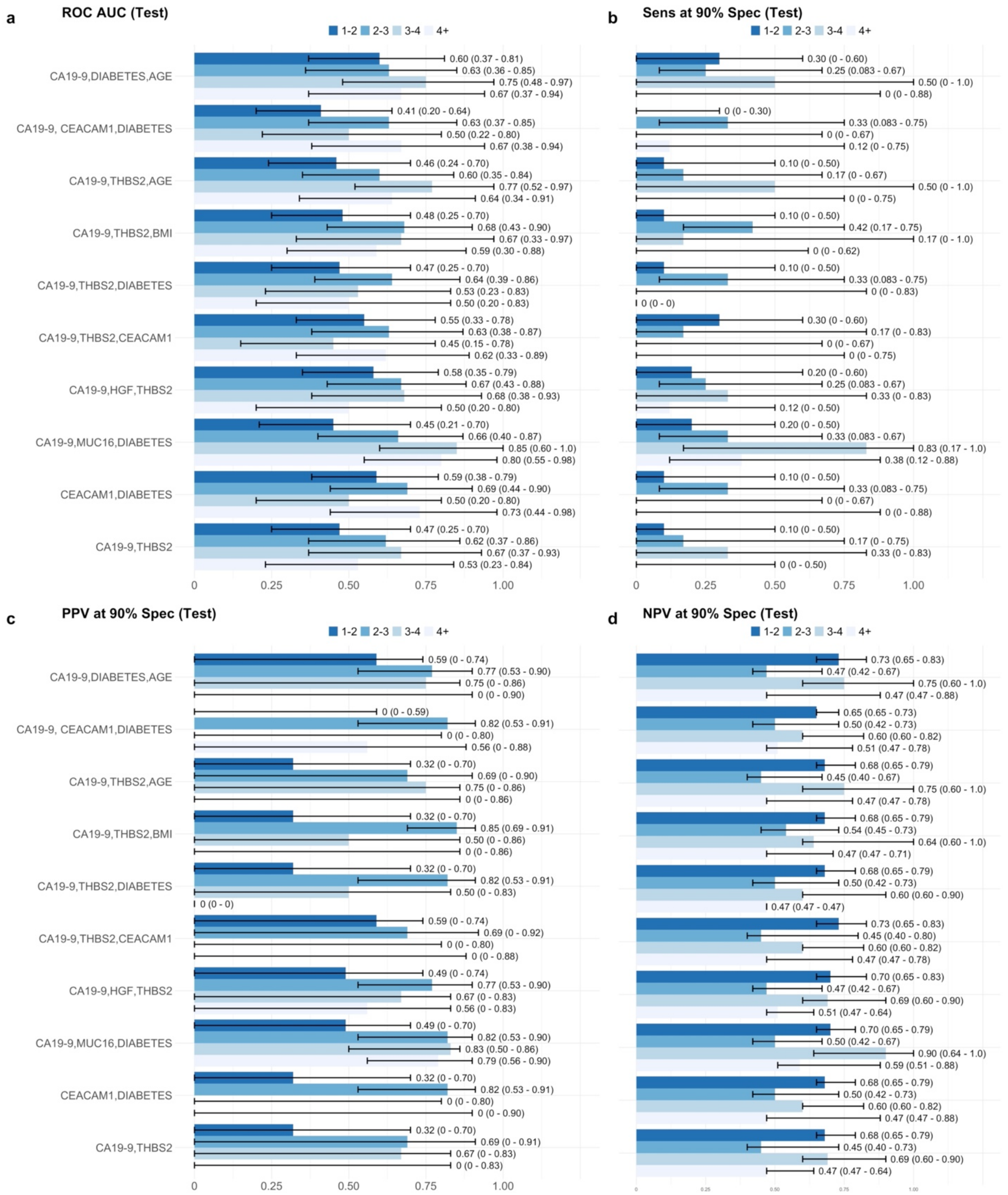

a
ROC AUC (Test)
1-2 2-3 3-4 4+
CA19-9,DIABETES,AGE
0.60 (0.37 - 0.81)
0.63 (0.36 - 0.85)
0.75 (0.48 - 0.97)
0.67 (0.37 - 0.94)
CA19-9, CEACAM1,DIABETES
0.41 (0.20 - 0.64)
0.63 (0.37 - 0.85)
0.50 (0.22 - 0.80)
0.67 (0.38 - 0.94)
CA19-9,THBS2,AGE
0.46 (0.24 - 0.70)
0.60 (0.35 - 0.84)
0.77 (0.52 - 0.97)
0.64 (0.34 - 0.91)
CA19-9,THBS2,BMI
0.48 (0.25 - 0.70)
0.68 (0.43 - 0.90)
0.67 (0.33 - 0.97)
0.59 (0.30 - 0.88)
CA19-9,THBS2,DIABETES
0.47 (0.25 - 0.70)
0.64 (0.39 - 0.86)
0.53 (0.23 - 0.83)
0.50 (0.20 - 0.83)
CA19-9,THBS2,CEACAM1
0.55 (0.33 - 0.78)
0.63 (0.38 - 0.87)
0.45 (0.15 - 0.78)
0.62 (0.33 - 0.89)
CA19-9,HGF,THBS2
0.58 (0.35 - 0.79)
0.67 (0.43 - 0.88)
0.68 (0.38 - 0.93)
0.50 (0.20 - 0.80)
CA19-9,MUC16,DIABETES
0.45 (0.21 - 0.70)
0.66 (0.40 - 0.87)
0.85 (0.60 - 1.0)
0.80 (0.55 - 0.98)
CEACAM1,DIABETES
0.59 (0.38 - 0.79)
0.69 (0.44 - 0.90)
0.50 (0.20 - 0.80)
0.73 (0.44 - 0.98)
CA19-9,THBS2
0.47 (0.25 - 0.70)
0.62 (0.37 - 0.86)
0.67 (0.37 - 0.93)
0.53 (0.23 - 0.84)
0 0.25 0.50 0.75 1.00
b
Sens at 90% Spec (Test)
1-2 2-3 3-4 4+
0.30 (0 - 0.60)
0.25 (0.083 - 0.67)
0.50 (0 - 1.0)
0 (0 - 0.88)
0 (0 - 0.30)
0.33 (0.083 - 0.75)
0 (0 - 0.67)
0.12 (0 - 0.75)
0.10 (0 - 0.50)
0.17 (0 - 0.67)
0.50 (0 - 1.0)
0 (0 - 0.75)
0.10 (0 - 0.50)
0.42 (0.17 - 0.75)
0.17 (0 - 1.0)
0 (0 - 0.62)
0.10 (0 - 0.50)
0.33 (0.083 - 0.75)
0 (0 - 0.83)
0 (0 - 0)
0.30 (0 - 0.60)
0.17 (0 - 0.83)
0 (0 - 0.67)
0 (0 - 0.75)
0.20 (0 - 0.60)
0.25 (0.083 - 0.67)
0.33 (0 - 0.83)
0.12 (0 - 0.50)
0.20 (0 - 0.50)
0.33 (0.083 - 0.67)
0.83 (0.17 - 1.0)
0.38 (0.12 - 0.88)
0.10 (0 - 0.50)
0.33 (0.083 - 0.75)
0 (0 - 0.67)
0 (0 - 0.88)
0.10 (0 - 0.50)
0.17 (0 - 0.75)
0.33 (0 - 0.83)
0 (0 - 0.50)
0 0.25 0.50 0.75 1.00
c
PPV at 90% Spec (Test)
1-2 2-3 3-4 4+
CA19-9,DIABETES,AGE
0.59 (0 - 0.74)
0.77 (0.53 - 0.90)
0.75 (0 - 0.86)
0 (0 - 0.90)
CA19-9, CEACAM1,DIABETES
0 (0 - 0.59)
0.82 (0.53 - 0.91)
0 (0 - 0.80)
0.56 (0 - 0.88)
CA19-9,THBS2,AGE
0.32 (0 - 0.70)
0.69 (0 - 0.90)
0.75 (0 - 0.86)
0 (0 - 0.86)
CA19-9,THBS2,BMI
0.32 (0 - 0.70)
0.85 (0.69 - 0.91)
0.50 (0 - 0.86)
0 (0 - 0.86)
CA19-9,THBS2,DIABETES
0.32 (0 - 0.70)
0.82 (0.53 - 0.91)
0.50 (0 - 0.83)
0 (0 - 0)
CA19-9,THBS2,CEACAM1
0.59 (0 - 0.74)
0.69 (0 - 0.92)
0 (0 - 0.80)
0 (0 - 0.88)
CA19-9,HGF,THBS2
0.49 (0 - 0.74)
0.77 (0.53 - 0.90)
0.67 (0 - 0.83)
0.56 (0 - 0.83)
CA19-9,MUC16,DIABETES
0.49 (0 - 0.70)
0.82 (0.53 - 0.90)
0.83 (0.50 - 0.86)
0.79 (0.56 - 0.90)
CEACAM1,DIABETES
0.32 (0 - 0.70)
0.82 (0.53 - 0.91)
0 (0 - 0.80)
0 (0 - 0.90)
CA19-9,THBS2
0.32 (0 - 0.70)
0.69 (0 - 0.91)
0.67 (0 - 0.83)
0 (0 - 0.83)
0 0.25 0.50 0.75 1.00
d
NPV at 90% Spec (Test)
1-2 2-3 3-4 4+
0.73 (0.65 - 0.83)
0.47 (0.42 - 0.67)
0.75 (0.60 - 1.0)
0.47 (0.47 - 0.88)
0.65 (0.65 - 0.73)
0.50 (0.42 - 0.73)
0.60 (0.60 - 0.82)
0.51 (0.47 - 0.78)
0.68 (0.65 - 0.79)
0.45 (0.40 - 0.67)
0.75 (0.60 - 1.0)
0.47 (0.47 - 0.78)
0.68 (0.65 - 0.79)
0.54 (0.45 - 0.73)
0.64 (0.60 - 1.0)
0.47 (0.47 - 0.71)
0.68 (0.65 - 0.79)
0.50 (0.42 - 0.73)
0.60 (0.60 - 0.90)
0.47 (0.47 - 0.47)
0.73 (0.65 - 0.83)
0.45 (0.40 - 0.80)
0.60 (0.60 - 0.82)
0.47 (0.47 - 0.78)
0.70 (0.65 - 0.83)
0.47 (0.42 - 0.67)
0.69 (0.60 - 0.90)
0.51 (0.47 - 0.64)
0.70 (0.65 - 0.79)
0.50 (0.42 - 0.67)
0.90 (0.64 - 1.0)
0.59 (0.51 - 0.88)
0.68 (0.65 - 0.79)
0.50 (0.42 - 0.73)
0.60 (0.60 - 0.82)
0.47 (0.47 - 0.88)
0.68 (0.65 - 0.79)
0.45 (0.40 - 0.73)
0.69 (0.60 - 0.90)
0.47 (0.47 - 0.64)

Supplementary Figure 23 Receiver Operating Curve (ROC) Area Under the Curve (AUC) performance of base-learners across training folds. a Joined time-groups. **b** Single time-groups. For a the number of independent samples used for training was n=107 (0-1), n=180 (0-2), n=252 (0-3), n=309 (0-4) and n=363 (0-4+). For b the number of independent samples was n=73 (1-2), n=72 (2-3), n=57 (3-4) and n=54 (4+). Different shades of blue correspond to different time-groups. See Supplementary table 12 for further details on case and control samples. See Statistical Analysis in Methods (main text) for further details.

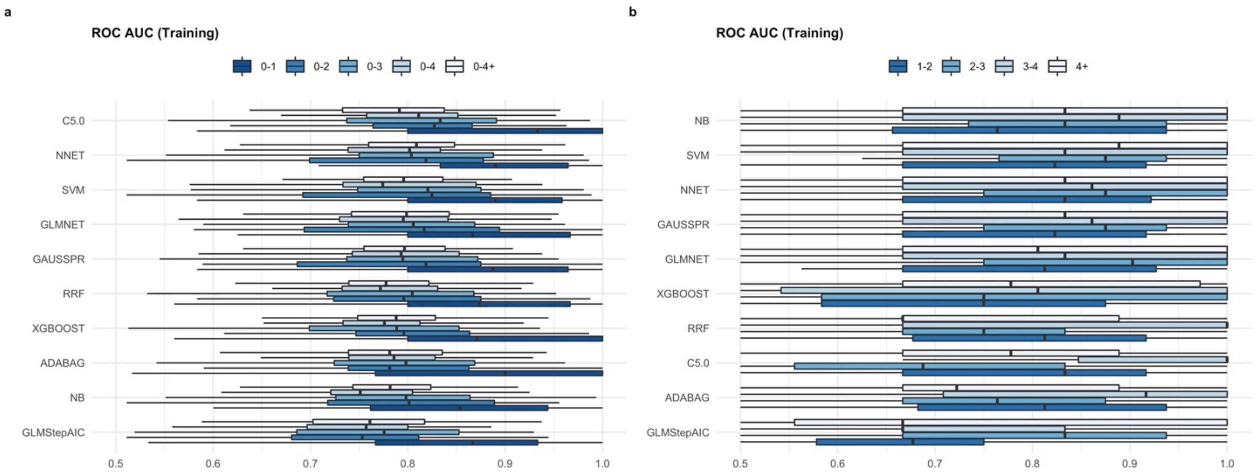

Supplementary Figure 24 Flow diagram for the joined time-group ensemble classifier, JTG2L. Each Joined Time Group 2 Layer (JTG2L) model presented in the main text, e.g., a model developed by training with 0-1 samples, was built according to this diagram. The feature matrix is used to train each of the base-learners. The output of this step is a probability vector of length N for each base-learner, where each entry corresponds to the probability of being a case according to the respective base-learner. The set of 10 probability vectors is then concatenated to generate a probability matrix that is used to train the meta-learner. From this a final probability output vector is computed. The base-learners and the meta-learner are trained with the resampling techniques highlighted in the methods section (main text). For the purpose of applying the resulting trained models to the test set, the flow of the diagram is the same as before but the feature matrix will have a different number of samples.

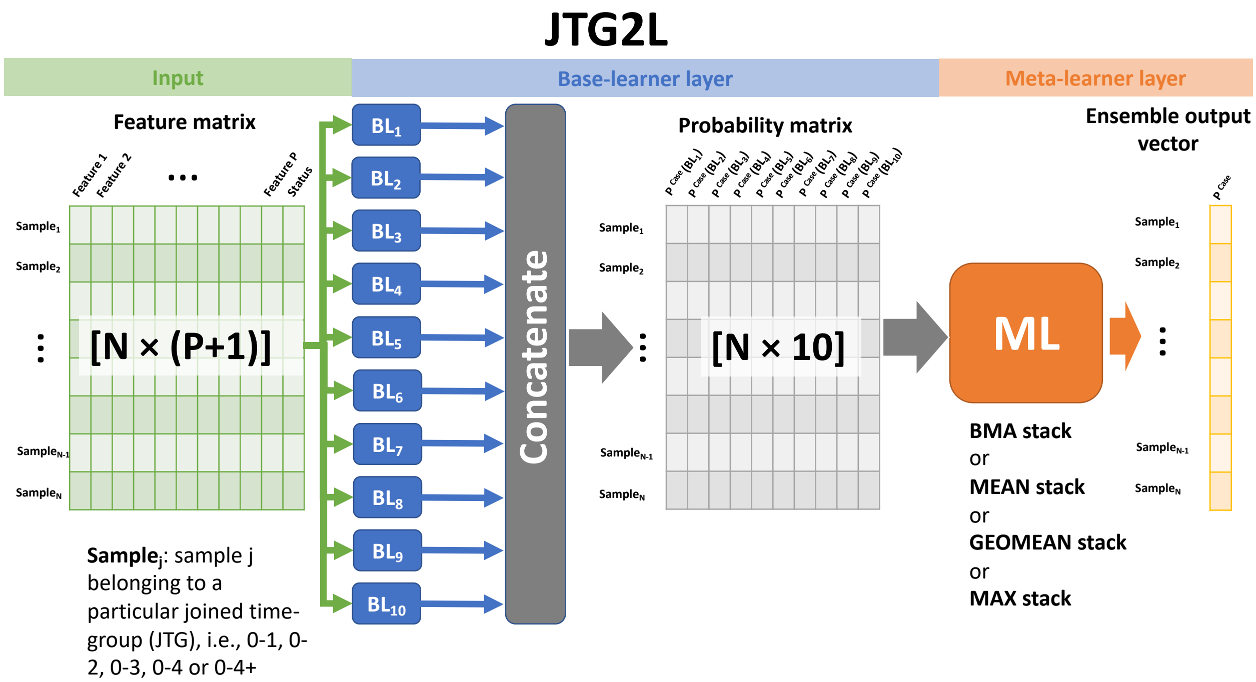

Supplementary Figure 25 Flow diagram for the specialized single time-group ensemble classifier, STG2L. Each Single Time Group 2 Layer (STG2L) model presented in the main text was built according to this diagram, where base-learners are trained in single time-groups and subsequently stacked. The stacking procedure has 2 steps. First, the probability output vectors for each base-learner trained in each single time-group are concatenated, thus leading to n probability matrices, where n is the number of single time-groups within a joined time group that we want to predict in the test set. If we are predicting pancreatic adenocarcinoma (PDAC) status in 0-2 samples, we will have 2 matrices. Second, these matrices are subsequently used to populate the diagonal blocks of a larger probability matrix. The off-diagonal probability blocks are generated by using the base models trained in a specific single time-group, which therefore amounts to computing cross time-group predictions. The resulting large matrix has 10*n columns and is then used to train the meta-learner which outputs the final vector. For the purposes of applying the resulting trained models to the test set, the flow of the diagram is the same as before, but the feature matrix will have a different number of samples.

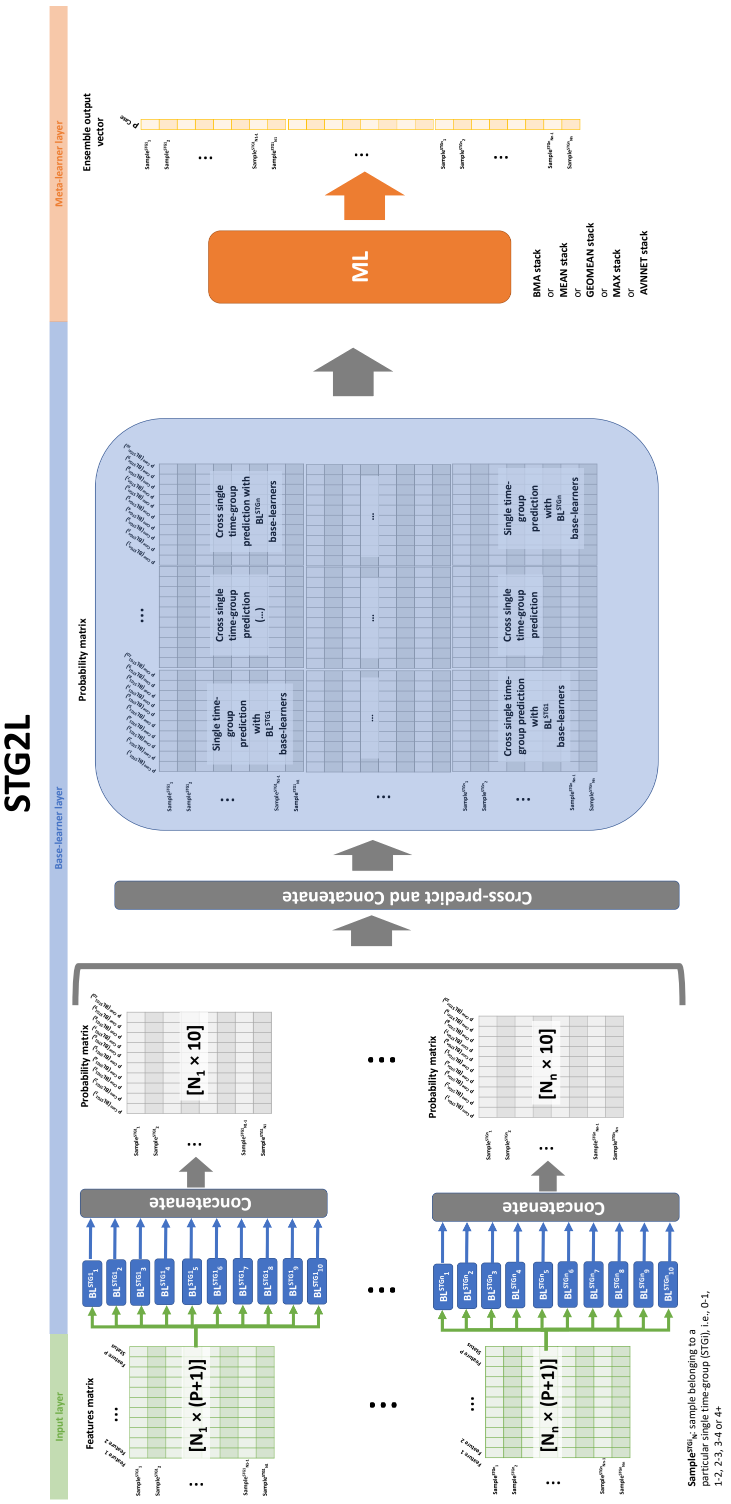
STG2L
Input layer
Base-learner layer
Meta-learner layer
Features matrix
[N1 × (P+1)]
[Nn × (P+1)]
Concatenate
Probability matrix
[N1 × 10]
[Nn × 10]
Cross-predict and Concatenate
Single time-group prediction with BLSTG1 base-learners
Cross single time-group prediction (…)
Cross single time-group prediction with BLSTGn base-learners
Cross single time-group prediction with BLSTG1 base-learners
Cross single time-group prediction
Single time-group prediction with BLSTGn base-learners
ML
BMA stack
or
MEAN stack
or
GEOMEAN stack
or
MAX stack
or
AVNNET stack
Ensemble output vector
SampleSTGiN: sample belonging to a particular single time-group (STGi), i.e., 0-1, 1-2, 2-3, 3-4 or 4+

Supplementary Figure 26 Flow diagram for the specialized single time-group ensemble classifier, STG3L. Each Single Time Group 3 Layer (STG3L) model presented in the main text was built according to this diagram, where base-learners are trained in single time-groups and subsequently stacked. The stacking procedure has 3 steps. First, the probability output vectors for each base-learner trained in each single time-group are concatenated, thus leading to n probability matrices, where n is the number of single time-groups within a joined time group that we want to predict in the test set. If we are predicting pancreatic adenocarcinoma (PDAC) status in 0-2 samples, we will have 2 matrices. Second, these matrices are used to train a BMA stack outputting 1-column probability vectors. From this intermediate step we have to populate the diagonal 1-column blocks of a larger probability matrix with n columns. The off-diagonal probability blocks are generated by using the base models and intermediate BMA stack trained in a specific single time-group, thus leading to cross time-group predictions. For the purposes of applying the resulting trained models to the test set, the flow of the diagram is the same as before, but the feature matrix will have a different number of samples.

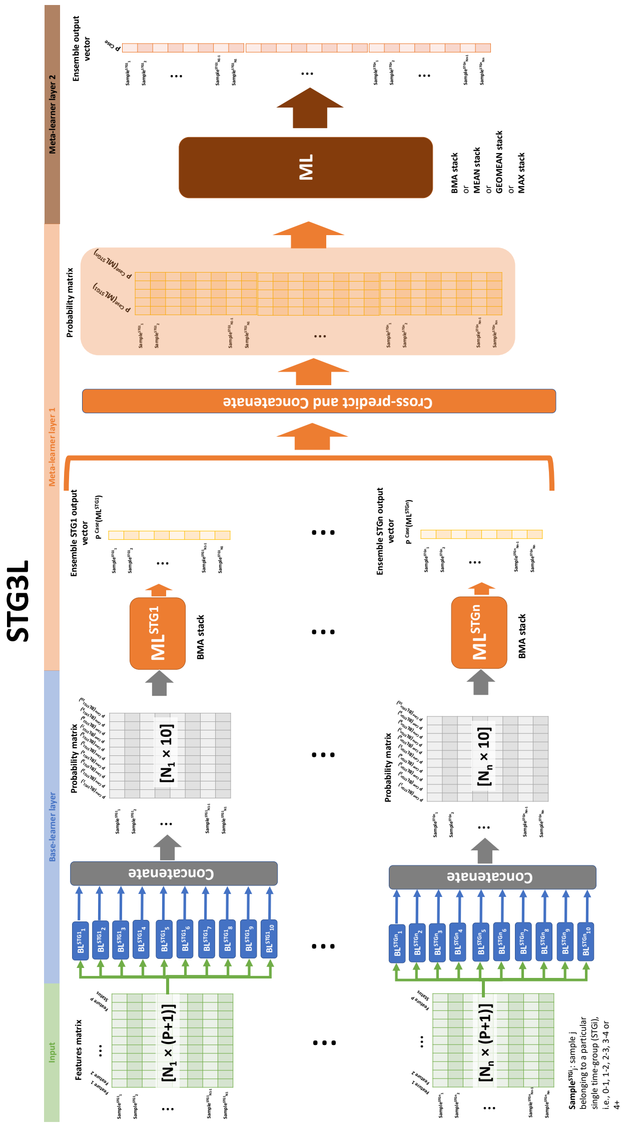
STG3L
Input
Base-learner layer
Meta-learner layer 1
Meta-learner layer 2
Features matrix
[N1 × (P+1)]
[Nn × (P+1)]
Concatenate
Probability matrix
[N1 × 10]
[Nn × 10]
BMA stack
Ensemble STG1 output vector
Ensemble STGn output vector
Cross-predict and Concatenate
Probability matrix
ML
BMA stack
or
MEAN stack
or
GEOMEAN stack
or
MAX stack
Ensemble output vector
Sample; sample j belonging to a particular single time-group (STGi), i.e., 0-1, 1-2, 2-3, 3-4 or 4+

Supplementary Figure 27 Performance for each of the meta-learners and models tested in this work. a Receiver Operating Curve **(**ROC) Area Under the Curve (AUC) across training folds for the Joined Time Group 2 Layer (JTG2L) model**.** Red error bars and dot represent the 95% Confidence Intervals (CI) for the mean and the mean, generated by bootstrapping with the *boot* R package (version 1.3-25)**. b** ROC AUC when the underlying base-learners and the stack is fitted to the full training set. **c** ROC AUC in the test set with the respective model developed in the training set**. d, e** and **f** Single Time Group 2 Layer (STG2L) model**. g, h** and **i** Single Time Group 3 Layer **(**STG3L) model**.** For a and b, the number of independent samples was n=107 (0-1) n=180 (0-2), n=252 (0-3), n=309 (0-4) and n=363 (0-4+). For c, n=107 (0-1), n=60 (0-2), n=82 (0-3), n=98 (0-4) and n=114 (0-4+). For d, e, g and h, n=107 (0-1), n=73 (1-2), n=72 (2-3), n=57 (3-4) and n=54 (4+). For f and i, n=26 (0-1), n=34 (1-2), n=22 (2-3), n=16 (3-4) and n=16 (4+). Different shades of blue correspond to different time-groups. See Statistical Analysis in Methods (main text) for further details. See also Supplementary Figure 28.

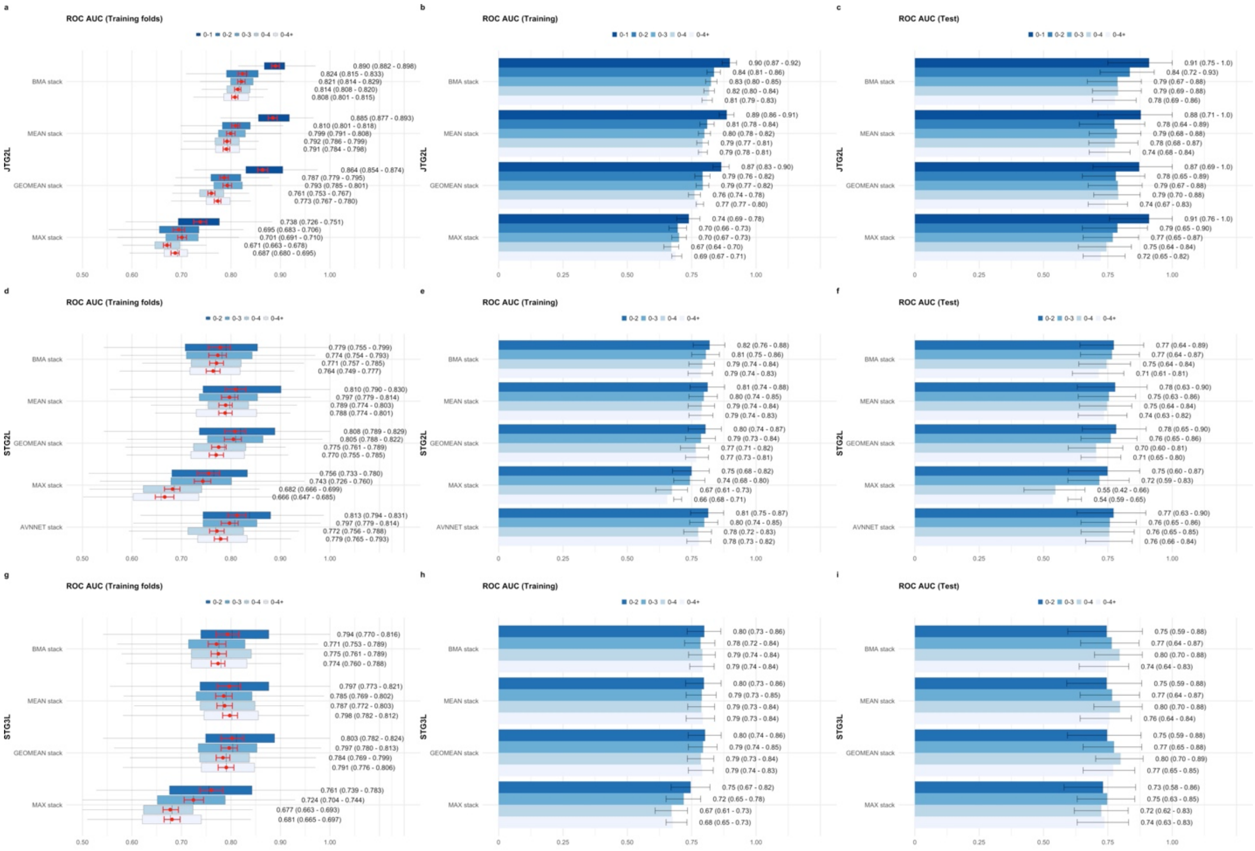

Supplementary Figure 28 Sensitivity, positive and negative predictive value for meta-learners and models tested in this work. a Sensitivity (Sens), **b** Positive predictive value (PPV) and **c** Negative predictive value (NPV) at 90% Specificity (Spec) for the Joined Time Group 2 Layer (JTG2L) model. **d, e** and **f** Single Time Group 2 Layer (STG2L) model**. g, h** and **i** Single Time Group 3 Layer (STG3L) model**.** All results were obtained with oversampling of minority class. Different shades of blue correspond to different time-groups. See Statistical Analysis in Methods (main text) for further details. See also Supplementary Figure 27.

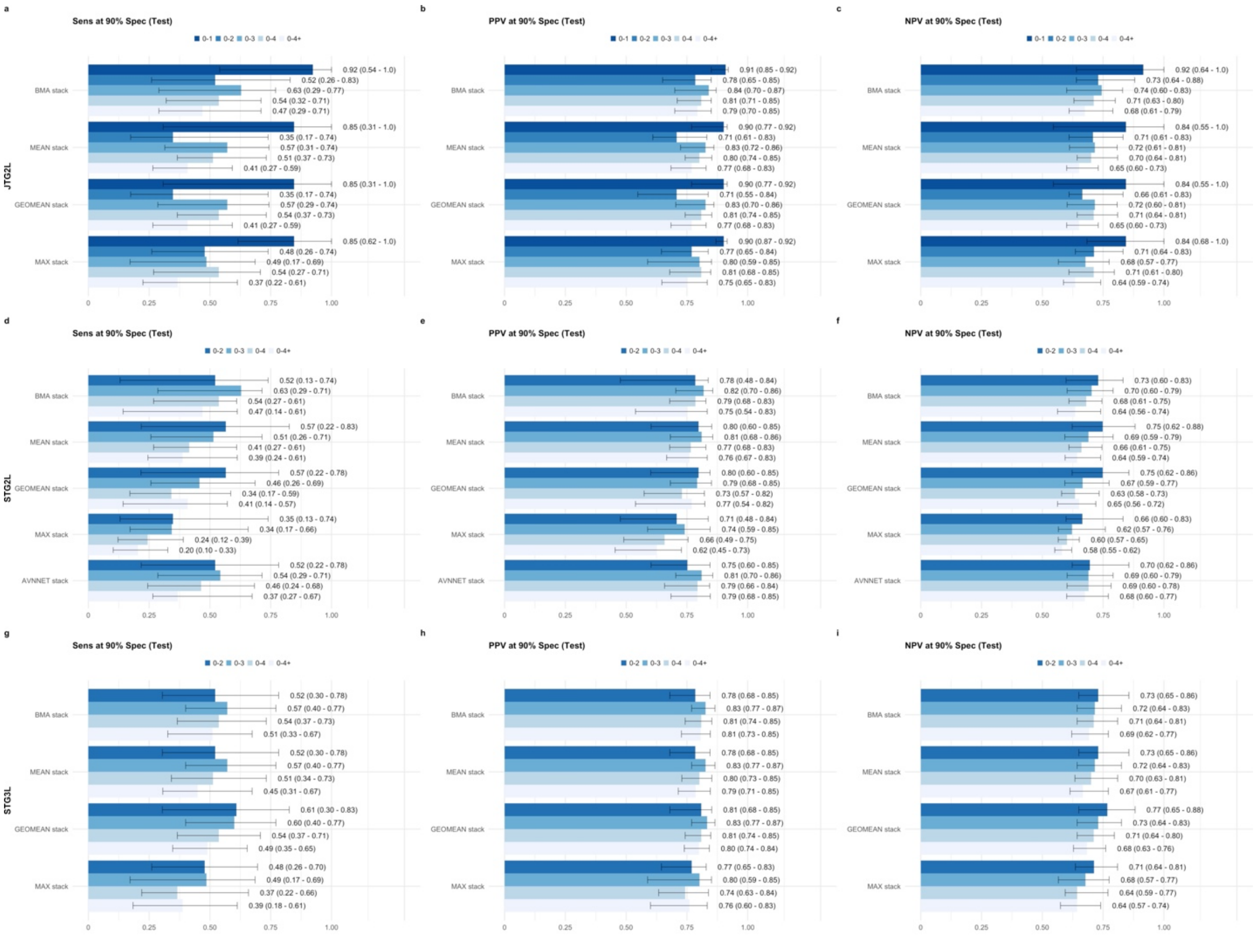

Supplementary Figure 29 Cross time-group performance for meta-learners, Joined Time Group 2 Layer (JTG2L). a and **b** Bayesian Model Averaging (BMA) meta-learner trained in joined time-groups, predicting joined time-groups and single time-groups, respectively. **c** and **d** MEAN meta-learner. **e** and **f** Geometric Mean (GEOMEAN) meta-learner. **g** and **h** MAX meta-learner. ns represents performance whose 95% Confidence Interval (CI) lower limit crosses the 0.5 threshold. See Statistical Analysis in Methods (main text) for further details.

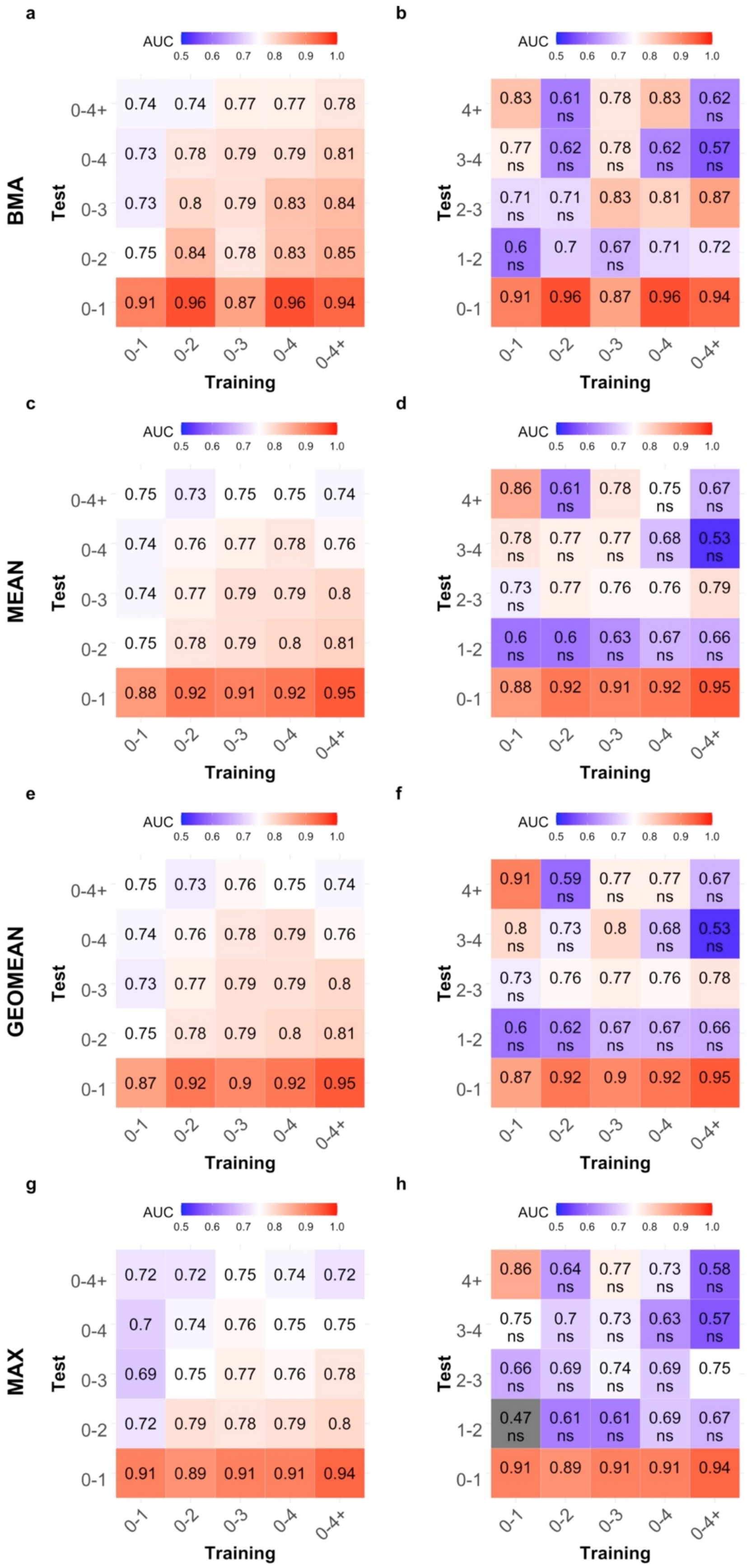
a
b
c
d
e
f
g
h
AUC
BMA
MEAN
GEOMEAN
MAX
Test
Training
0-1
0-2
0-3
0-4
0-4+
1-2
2-3
3-4
4+
ns

Supplementary Figure 30 Matthews correlation coefficients (MCC) at 90% Specificity (Spec) for the Joined Time Group 2 Layer (JTG2L) model with a Bayesian Model Averaging (BMA) stack meta-learner shown in Fig. 1. a Same time-group performance (see also Fig. 1 d, e and f for sensitivities, positive predictive values and negative predictive values). **b** Cross time-group performances for the ensemble trained in 0-4+ samples and tested in 0-1, 0-2, 0-3 and 0-4 samples in the test set (last column in Fig. 1c, see also Fig. 1 g, h, i for sensitivities, positive predictive values and negative predictive values). The red dots and respective numbers correspond to the performance in the full test set for the respective time-group samples. The boxplots correspond to the distribution of MCC values calculated from the true positives, true negatives, false positives and false negatives, from the same stratified bootstraps as those in Figure 1. The number of independent training samples was n=107 (0-1) n=180 (0-2), n=252 (0-3), n=309 (0-4) and n=363 (0-4+), and test set samples n=26 (0-1), n=60 (0-2), n=82 (0-3), n=98 (0-4) and n=114 (0-4+). Different shades of blue correspond to different time-groups. See Statistical Analysis in Methods (main text) for further details.

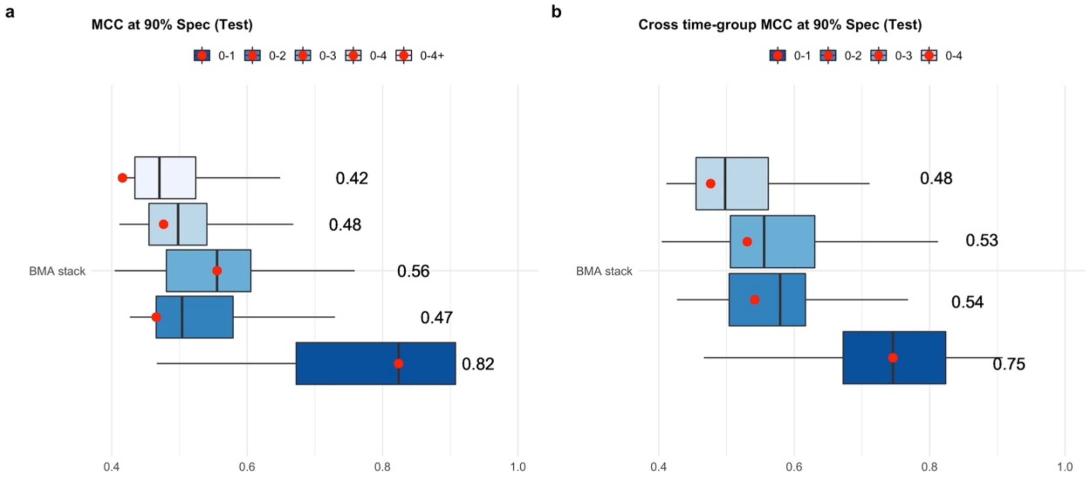

Supplementary Figure 31 Performance for meta-learners with HD-DPM and SMOTE synthetic data generation during resampling, for the JTG2L model. **a** ROC AUC across training folds for the JTG2L model with synthetic data generated by a high-dimensional Dirichlet Mixture model (HD-DPM)**.** Red error bars and dot represent the 95% Confidence Interval (CI) for the mean and the mean, generated by bootstrapping with the *boot* R package (version 1.3-25)**. b** Receiver Operating Curve (ROC) Area Under the Curve (AUC) when the underlying base-learners and the stack is fitted to the full training set. **c** ROC AUC in the test set with the respective models developed in the training set**. d, e** and **f** Similar to a, b and c but with the Synthetic Minority Oversampling Technique (SMOTE). For a, b, d and e the total number of independent training samples used for synthetic data generation was n=107 (0-1) n=180 (0-2), n=252 (0-3), n=309 (0-4) and n=363 (0-4+). For c and f the number of test set samples was n=26 (0-1), n=60 (0-2), n=82 (0-3), n=98 (0-4) and n=114 (0-4+). Different shades of blue correspond to different time-groups. See Statistical Analysis in Methods (main text) for further details.

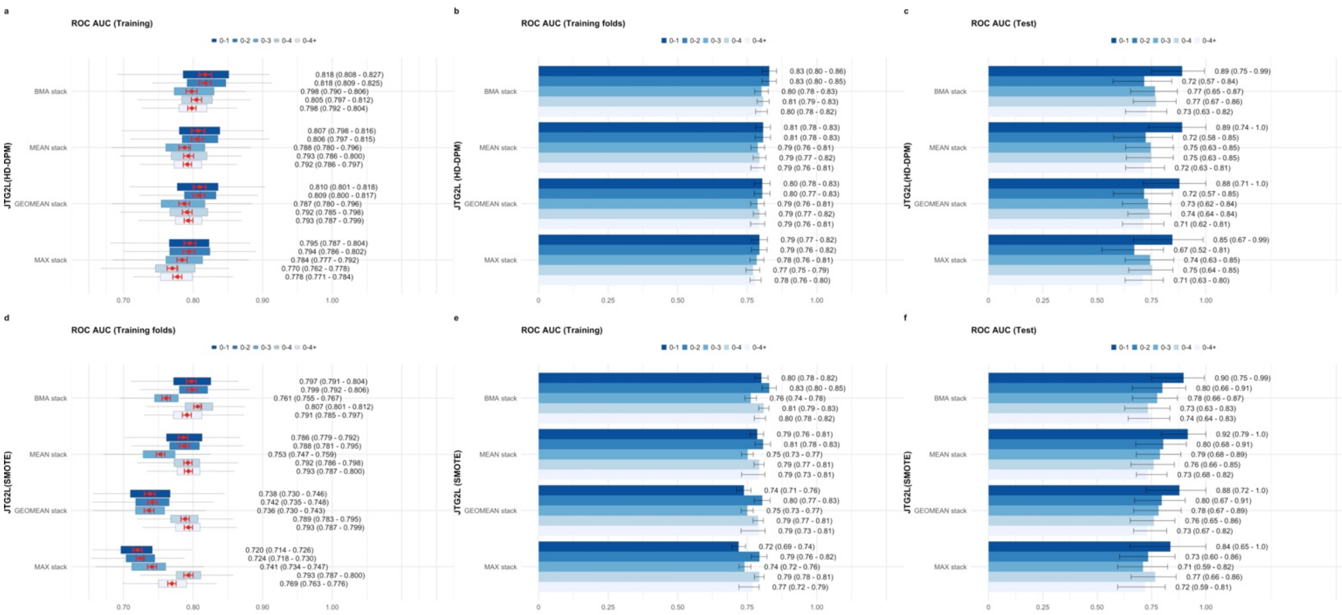

Supplementary Figure 32 Sensitivity, positive and negative predictive value for meta-learners with HD-DPM and SMOTE during resampling, for the Joined Time Group 2 Layer (JTG2L) model. a, b and **c** Sensitivity (Sens), Positive predictive value (PPV) and Negative Predictive Value (NPV) at 90% Specificity (Spec) when the underlying base-learners and the stack is fitted to the full training set but trained with synthetic data generated by a high-dimensional Dirichlet Mixture model (HD-DPM) and is applied to the test set. **d, e** and **f** Similar to a, b and c but with the Synthetic Minority Oversampling Technique (SMOTE). For a, b, d and e the total number of independent training samples used for synthetic data generation was n=107 (0-1) n=180 (0-2), n=252 (0-3), n=309 (0-4) and n=363 (0-4+). For c and f the number of test set samples was n=26 (0-1), n=60 (0-2), n=82 (0-3), n=98 (0-4) and n=114 (0-4+). See Supplementary Figure 31. Different shades of blue correspond to different time-groups. See Statistical Analysis in Methods (main text) for further details.

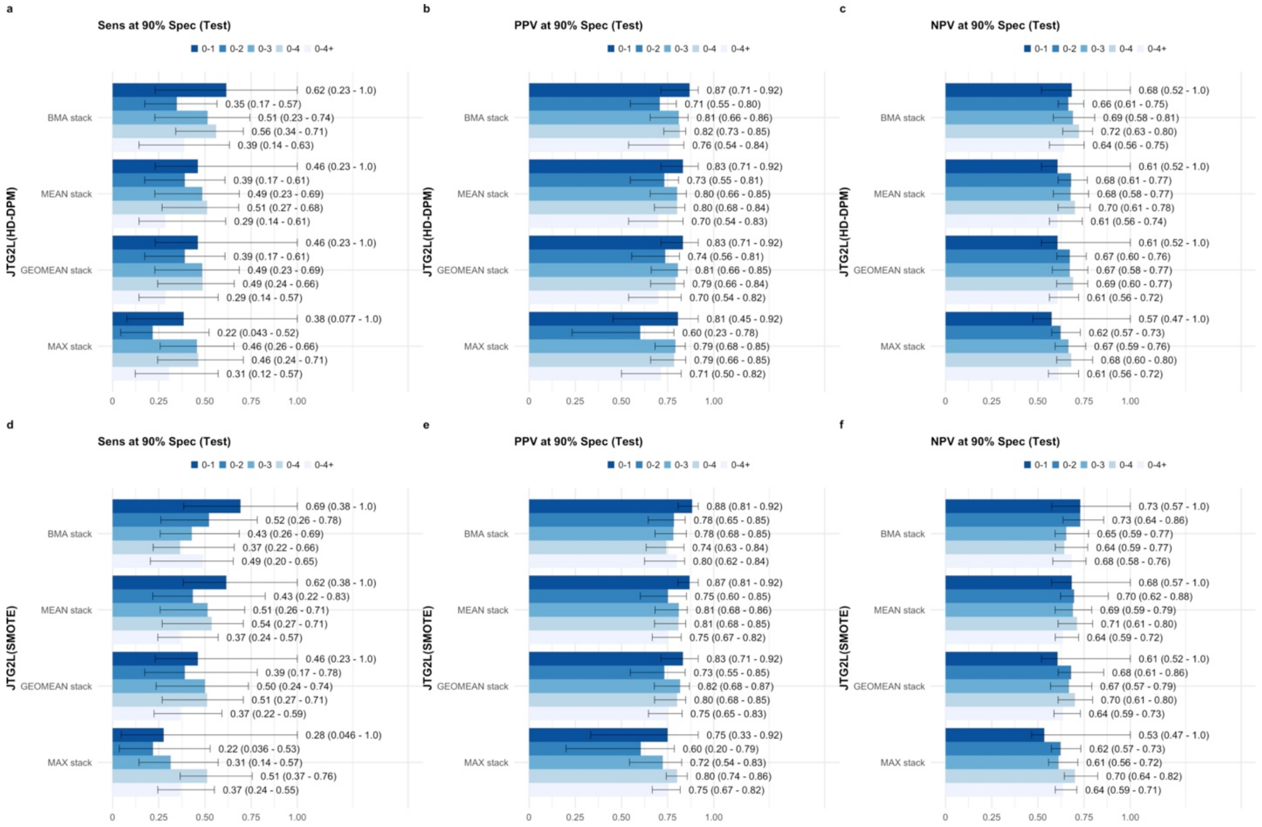

Supplementary Figure 33 Signatures for each joined time-group across all base-learners. a P values for each feature were calculated according to a logistic regression model with a bias reduction method for each of the features in a one-dimensional model (see Methods). Size corresponds to Odds ratio (OR). **b** Feature importance across base-learners according to a model-agnostic method based on a simple feature importance ranking measure, implemented in the R package *vip*. See Fig 2. Different shades of blue correspond to different time-groups. See Statistical Analysis in Methods (main text) for further details.

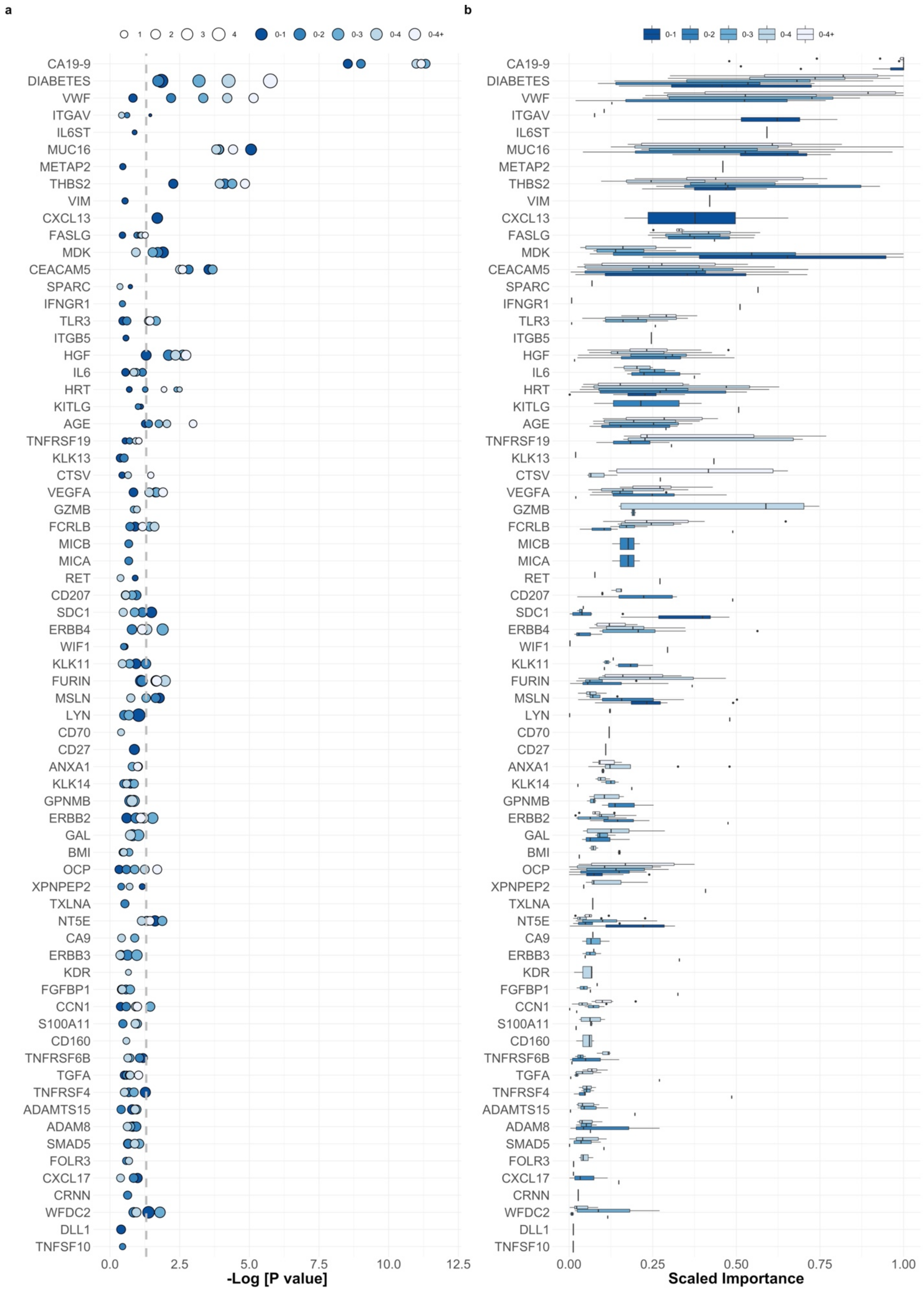
a
1
2
3
4
0-1
0-2
0-3
0-4
0-4+
CA19-9
DIABETES
VWF
ITGAV
IL6ST
MUC16
METAP2
THBS2
VIM
CXCL13
FASLG
MDK
CEACAM5
SPARC
IFNGR1
TLR3
ITGB5
HGF
IL6
HRT
KITLG
AGE
TNFRSF19
KLK13
CTSV
VEGFA
GZMB
FCRLB
MICB
MICA
RET
CD207
SDC1
ERBB4
WIF1
KLK11
FURIN
MSLN
LYN
CD70
CD27
ANXA1
KLK14
GPNMB
ERBB2
GAL
BMI
OCP
XPNPEP2
TXLNA
NT5E
CA9
ERBB3
KDR
FGFBP1
CCN1
S100A11
CD160
TNFRSF6B
TGFA
TNFRSF4
ADAMTS15
ADAM8
SMAD5
FOLR3
CXCL17
CRNN
WFDC2
DLL1
TNFSF10
0.0
2.5
5.0
7.5
10.0
12.5
-Log [P value]
b
0-1
0-2
0-3
0-4
0-4+
0.00
0.25
0.50
0.75
1.00
Scaled Importance

Supplementary Figure 34 Signatures for each single time-group across all base-learners. a P values for each feature were calculated according to a logistic regression model with a bias reduction method for each of the features in a one-dimensional model. Size corresponds to Odds ratio (OR). **b** Feature importance across base-learners according to a model-agnostic method based on a simple feature importance ranking measure, implemented in the R package *vip*. Different shades of blue correspond to different time-groups. See Statistical Analysis in Methods (main text) for further details.

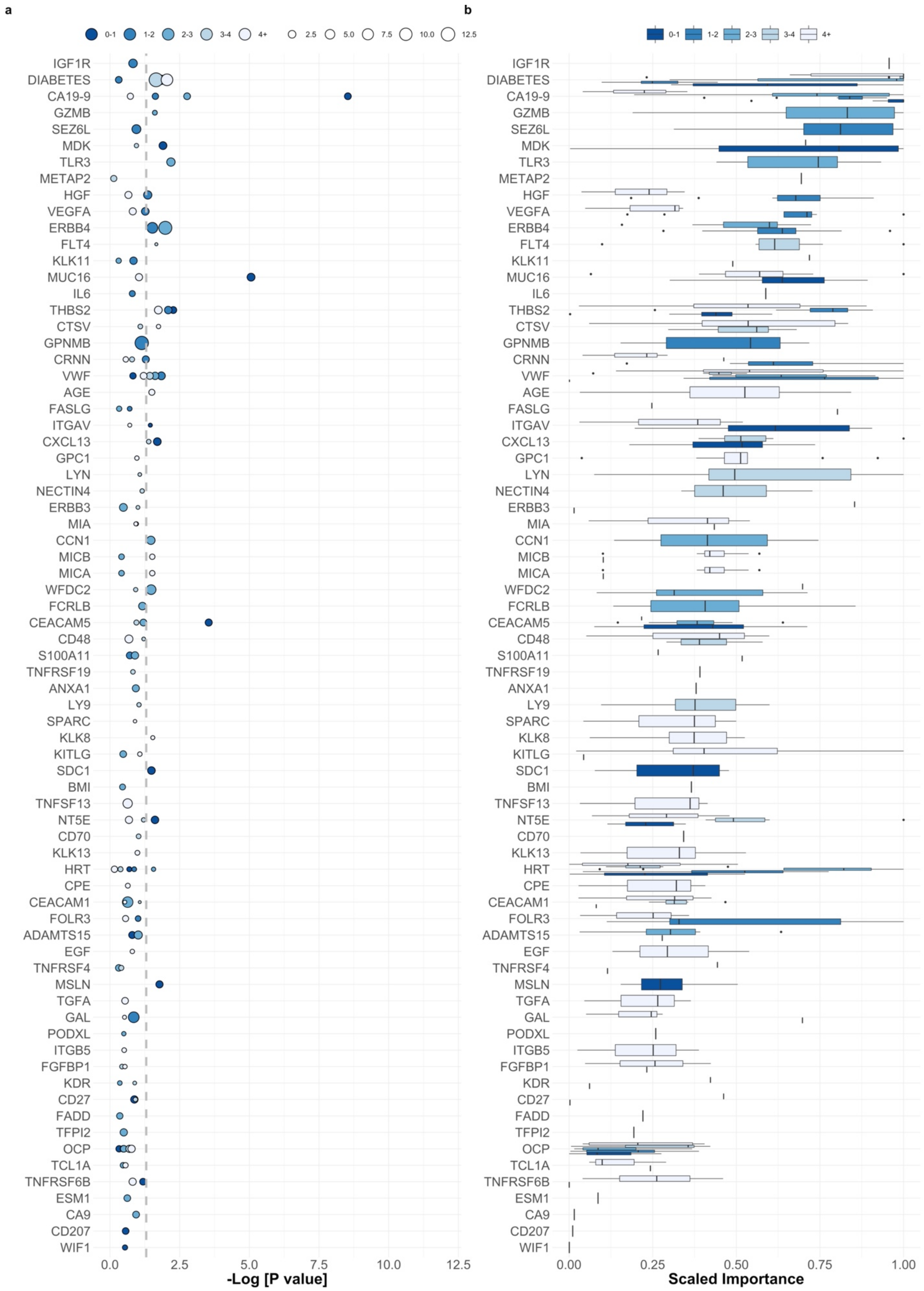
a
b
0-1 1-2 2-3 3-4 4+
2.5 5.0 7.5 10.0 12.5
IGF1R
DIABETES
CA19-9
GZMB
SEZ6L
MDK
TLR3
METAP2
HGF
VEGFA
ERBB4
FLT4
KLK11
MUC16
IL6
THBS2
CTSV
GPNMB
CRNN
VWF
AGE
FASLG
ITGAV
CXCL13
GPC1
LYN
NECTIN4
ERBB3
MIA
CCN1
MICB
MICA
WFDC2
FCRLB
CEACAM5
CD48
S100A11
TNFRSF19
ANXA1
LY9
SPARC
KLK8
KITLG
SDC1
BMI
TNFSF13
NT5E
CD70
KLK13
HRT
CPE
CEACAM1
FOLR3
ADAMTS15
EGF
TNFRSF4
MSLN
TGFA
GAL
PODXL
ITGB5
FGFBP1
KDR
CD27
FADD
TFPI2
OCP
TCL1A
TNFRSF6B
ESM1
CA9
CD207
WIF1
0.0 2.5 5.0 7.5 10.0 12.5
-Log [P value]
0.00 0.25 0.50 0.75 1.00
Scaled Importance

Supplementary Figure 35 Heterogeneity between base-learners, single time-groups. Cohen k-statistic (kappa) for pair-wise base-learner predictions in the test set. All values are significant. For a, b, c and d the number of independent training samples was n=107 (0-1), n=73 (1-2), n=72 (2-3), n=57 (3-4) and n=54 (4+), and test set samples where prediction was done was n=34 (1-2), n=22 (2-3), n=16 (3-4) and n=16 (4+). See Supplementary table 12 for further details on case and control samples. See Statistical Analysis in Methods (main text) for further details.

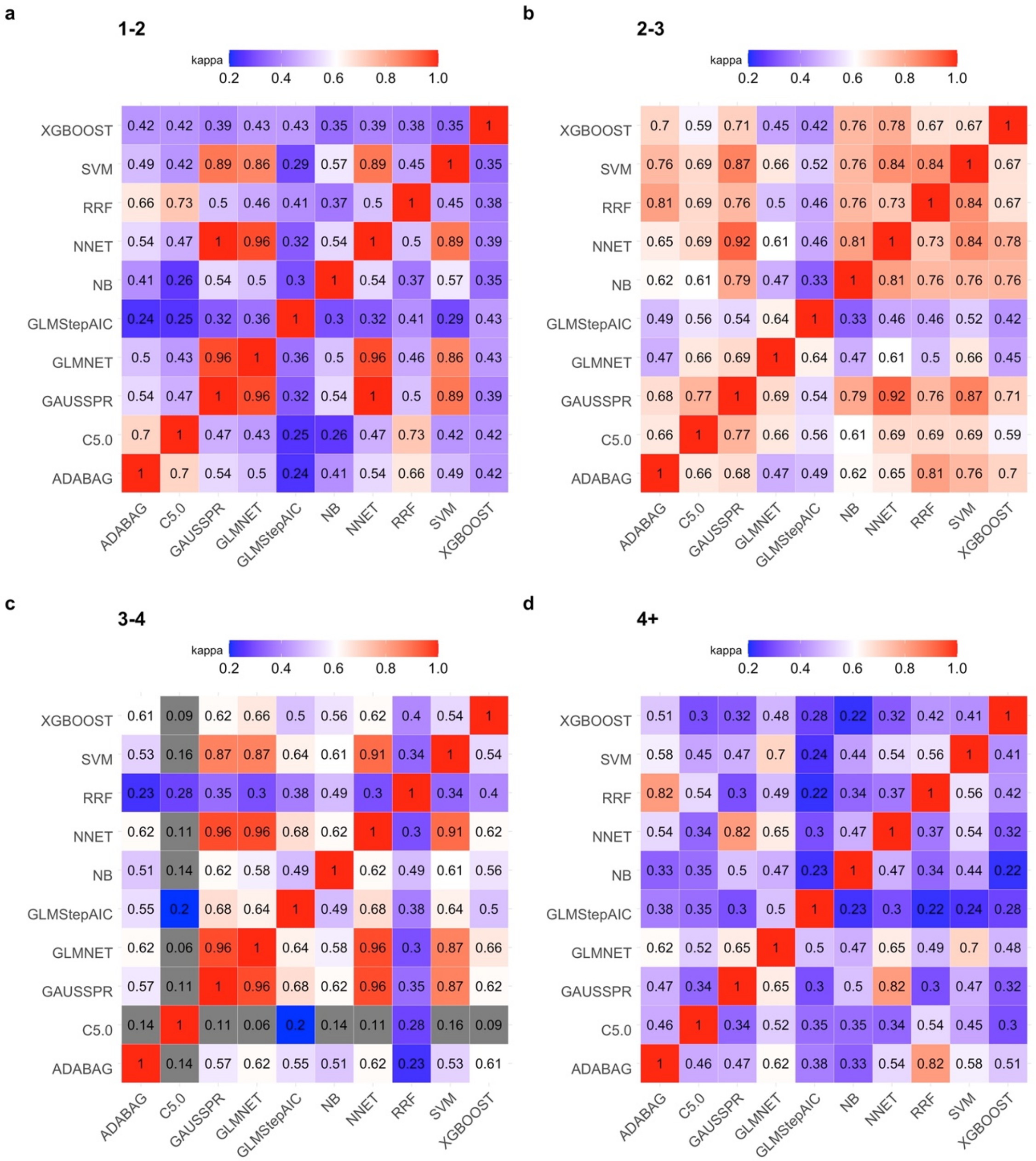

Supplementary Figure 36 Heterogeneity between base-learners, joined time-groups. Cohen k-statistic (kappa) for pair-wise base-learner predictions in the test set. All values are significant. For a, b, c, d and e the number of training For a, b, c and d the number of independent training samples was n=107 (0-1), n=180 (0-2), n=252 (0-3), n=309 (0-4) and n=363 (0-4+), and test samples n=26 (0-1), n=60 (0-2), n=82 (0-3), n=98 (0-4) and n=114 (0-4+), and test samples where prediction was done n=26 (0-1), n=60 (0-2), n=82 (0-3), n=98 (0-4) and n=114 (0-4+). See Supplementary table 12 for further details on case and control samples. See Statistical Analysis in Methods (main text) for further details.

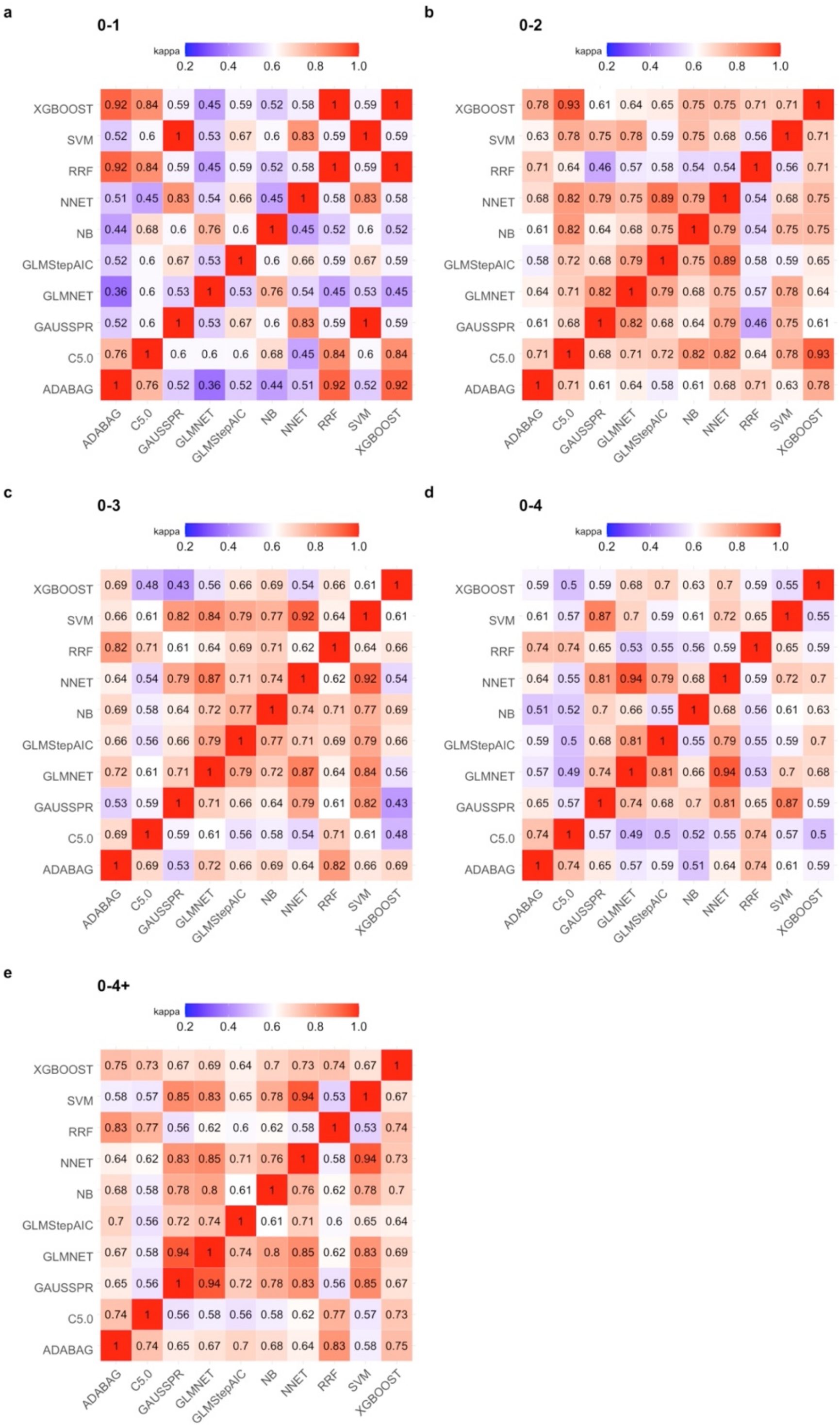

Supplementary Figure 37 Performance at different prevalence values for the Joined Time Group 2 Layer (JTG2L) Bayesian Model Averaging (BMA) stack. **a** Sensitivity (Sens). **b** Positive predictive (PPV). **c** Negative predictive (NPV) at 90% Specificity (Spec). Prevalence is determined by the #Cases/(#Cases+#Controls) ratio times 100. For each prevalence value, we select randomly (Prevalence* #Controls/(1-Prevalence)) Cases from the pool of Cases in the test set and determine the performance of the classifiers by using these Cases against the full set of Controls in the test set. This was repeated 1000 times. Red error bars and dot represent the 95% Confidence Intervals (CI) for the mean and the mean, generated by bootstrapping with the *boot* R package (version 1.3-25)**.** The 0-1 time-group was not tested since the number of cases was not sufficient to perform the study at 8% and 16% prevalence. See also Supplementary Figure 38. Different shades of blue correspond to different time-groups. See Statistical Analysis in Methods (main text) for further details.

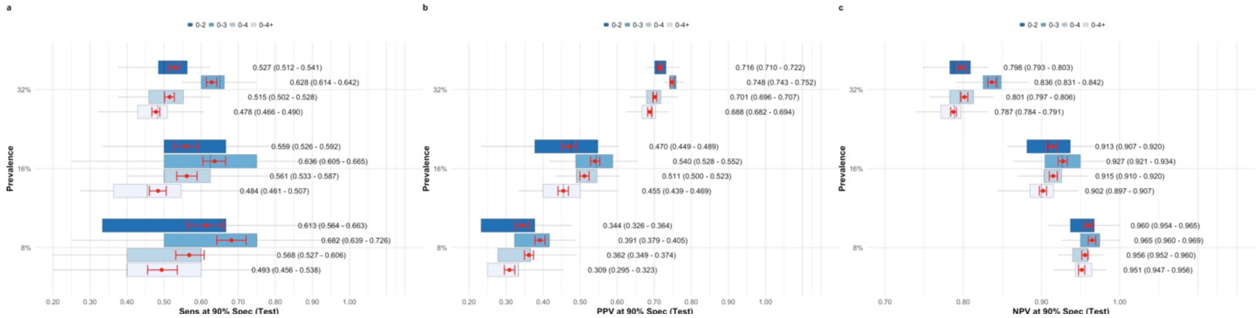

Supplementary Figure 38 Receiver Operating Curve (ROC) Area Under the Curve (AUC) performance at different prevalence values for the Joined Time Group 2 Layer (JTG2L) Bayesian Model Averaging (BMA) stack. Prevalence is determined by the #Cases/(#Cases+#Controls) ratio times 100. For each prevalence value, we select randomly (Prevalence* #Controls/(1-Prevalence)) Cases from the pool of Cases in the test set and determine the performance of the classifiers by using these Cases against the full set of Controls in the test set. This was repeated 1000 times. Red error bars and dot represent the 95% Confidence Intervals (CI) for the mean and the mean, generated by bootstrapping with the *boot* R package (version 1.3-25)**.** The 0-1 time-group was not tested since the number of cases was not sufficient to perform the study at 8% and 16% prevalence. Different shades of blue correspond to different time-groups.

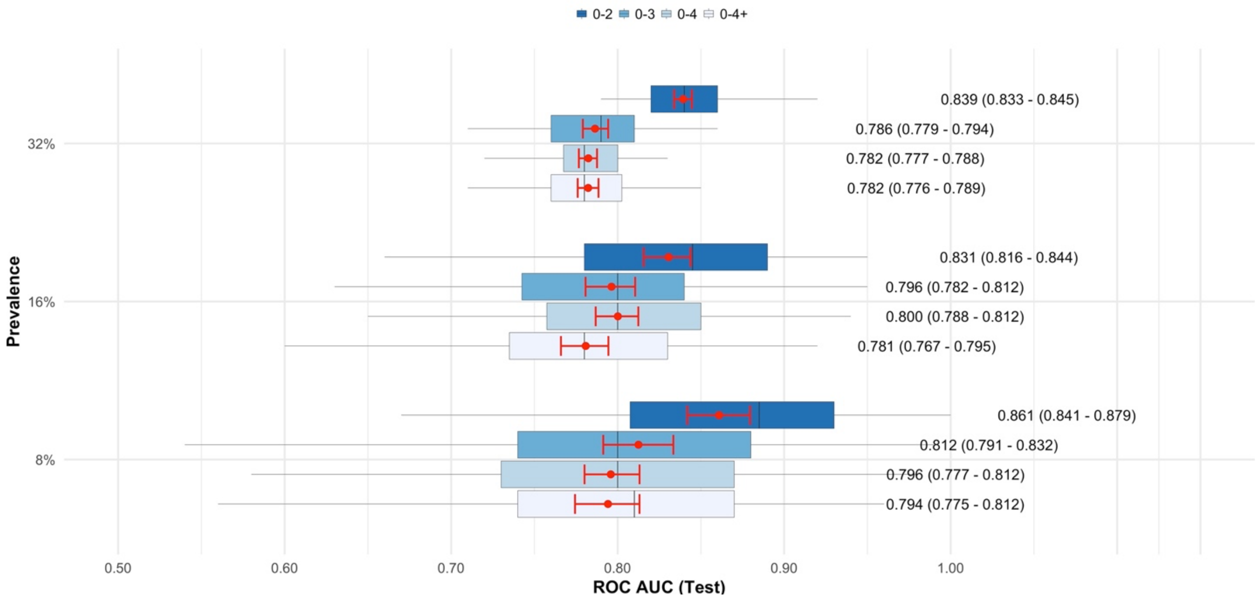

Supplementary Figure 39 Matthews correlation coefficient (MCC) calculated for the external validation dataset collected from the ADEPTS cohort. The performances correspond to 1000 datasets whose difference from the original ADEPTS subset selected for this study is the random allocation of the missing features Hormone Replacement Therapy (HRT) and Oral Contraceptive Pill (OCP) use to female participants. The red dots and respective numbers correspond to estimates of the mean performance (by bootstrapping with the *boot* R package (version 1.3-25)) for the respective model developed in UKCTOCS time-grouped samples. See Study Design and Statistical Analysis sections in Methods for further details, and Supplementary Data 7. The total number of independent UKCTOCS training samples used was n=107 (0-1) n=180 (0-2), n=252 (0-3), n=309 (0-4) and n=363 (0-4+). The number of independent samples from ADEPTS where the models were tested was n=34. See also Figure 4.

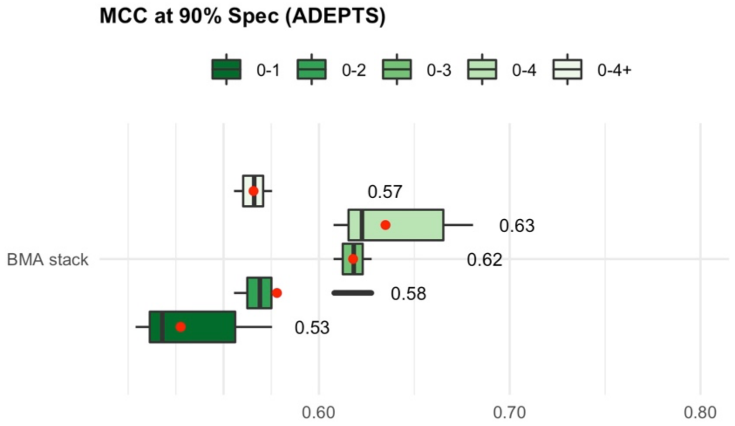

Supplementary Tables:

Supplementary Table 1 Training set characteristics. P values were calculated according to a logistic regression model with a bias reduction method (see Methods), for the whole distribution in the training set. This table corresponds to taking all samples from all time-groups, i.e., 0 to 4+. See also Supplementary Figure 1 and 2. na: not applicable. BMI represents Body Mass Index. HRT stands for Hormone Replacement Therapy. OCP represents Oral Contraceptive Pill use, ever. OR stands for odds ratio.

Variable		Cases	Controls	*P* value
No. samples		168	185	
Tumour site				
	tail	8	na	
	body	17	na	
	body/tail	2	na	
	head	67	na	
	unspecified	74	na	
Mean time to spin (h) (range)		21.64 (4.65 – 46.53)	21.85 (4.88 - 46.01)	0.38
Mean age at sample draw (yr) (range)		64.63 (51.19 - 74.87)	62.32 (50.44 - 76.08)	0.0010 (OR=1.06)
Mean BMI (kg/m2) (range)		27.83 (18.34 - 43.74)	27.19 (17.91 – 44.39)	0.25
Mean time from sample collection to diagnosis (months) (range)		25.9 (1.02 - 70.09)	na	
HRT use (at randomisation)				
	yes	18	38	0.012 (OR=0.47)
	no	150	147	
OCP use (ever)				
	yes	106	94	0.02 (OR=1.65)
	no	63	91	
Diabetes				
	yes	38	10	<0.0001 (OR=4.93)
	no	130	175	

Supplementary Table 2 Test set characteristics. P values were calculated according to a logistic regression model with a bias reduction method (see Methods), for the whole distribution in the test set. This table corresponds to taking all samples from all time-groups, i.e., 0 to 4+. See also Supplementary Figure 1 and 2. na: not applicable. BMI represents Body Mass Index. HRT stands for Hormone Replacement Therapy. OCP represents Oral Contraceptive Pill use, ever. OR stands for odds ratio.

Variable		Cases	Controls	*P* value
No. samples		50	64	
Tumour site				
	tail	5	na	
	body	2	na	
	body/tail	1	na	
	head	21	na	
	unspecified	21	na	
Mean time to spin (h) (range)		22.03 (2 – 46.35)	21.61 (0.32 – 46.24)	0.75
Mean age at sample draw (yr) (range)		65.96 (52.28 - 74.12)	62.96 (50.59 – 76.86)	0.0088 (OR=1.08)
Mean BMI (kg/m2) (range)		26.21 (17.80 – 42.72)	25.04 (19.74 - 35.14)	0.13
Mean time from sample collection to diagnosis (months) (range)		26.61 (0.99 - 66.07)	n.a.	
HRT use (at randomisation)				
	yes	1	10	0.013 (OR=0.16)
	no	49	54	
OCP use (ever)				
	yes	26	33	0.96
	no	24	31	
Diabetes				
	yes	4	1	0.11
	no	46	63	

Supplementary Table 3 External validation set characteristics that concerns to pancreatic ductal adenocarcinoma (PDAC) stage. Samples were collected from the ADEPTS cohort (see Study Design section in Methods).

Variable	Cases	Controls
No. samples	17	17
PDAC stage		
1	2	na
2	3	na
3	3	na
4	6	na
Unknown	3	na

Supplementary Table 4 Quantitative ELISA assays` intra-assay coefficient of variation. *CLIA assay, Cobas CA125 II CLIA, was run on Cobas E411 analyser. na stands for not applicable.

Assay	Dilution factor	CV (%)
CA19-9(A)	1:4	6.9
CA125/MUC16 (A)*	na	4.1
LRG1	1:2000	5.5
PIGR	1:500	15.5
REG3A/PAP	1:100	21.9
Factor XII (F12)	1:1000	10.5
vWF	1:100	13.5
THBS1	1:100	17.9
AGR2	1:25	18.5
THBS2	1:10	12.5

Supplementary Table 5 Subset characteristics within the training set for which AFP, TEK and IGFBP1 were also measured. P values were calculated according to a logistic regression model with a bias reduction method (see Methods), for the whole distribution. This table corresponds to taking all samples from all time-groups, i.e., 0 to 4+. na: not applicable. See Methods section in the main text for details. BMI represents Body Mass Index. HRT stands for Hormone Replacement Therapy. OCP represents Oral Contraceptive Pill use, ever. OR stands for odds ratio.

Variable		Cases	Controls	*P* value
No. samples		126	132	
Tumour site				
	tail	6	na	
	body	12	na	
	body/tail	1	na	
	head	49	na	
	unspecified	58	na	
Mean time to spin (h) (range)		21.38 (0.6 – 46.53)	21.90 (0.58 – 46.01)	0.56
Mean age at sample draw (yr) (range)		64.63 (51.19 – 74.87)	62.56 (50.44 – 76.08)	0.01 (OR=1.05)
Mean BMI (kg/m2) (range)		27.83 (18.34 – 43.74)	27.03 (41.09 – 17.91)	0.20
Mean time from sample collection to diagnosis (months) (range)		26.31 (1.02 – 70.09)	n.a.	
HRT use (at randomisation)				
	yes	15	26	0.089
	no	111	106	
OCP use (ever)				
	yes	87	65	0.0012 (OR=2.28)
	no	39	67	
Diabetes				
	yes	27	7	0.00011 (OR=4.62)
	no	99	125	

Supplementary Table 6 Subset characteristics within the test set for which AFP, TEK and IGFBP1 were also measured. P values were calculated according to a logistic regression model with a bias reduction method (see Methods), for the whole distribution. This table corresponds to taking all samples from all time-groups, i.e., 0 to 4+. na: not applicable. See Methods in the main text for details. BMI represents Body Mass Index. HRT stands for Hormone Replacement Therapy. OCP represents Oral Contraceptive Pill use, ever. OR stands for odds ratio.

Variable		Cases	Controls	*P* value
No. samples		32	44	
Tumour site				
	tail	2	na	
	body	4	na	
	body/tail	0	na	
	head	12	na	
	unspecified	14	na	
Mean time to spin (h) (range)		23.75 (3.22 – 46.35)	21.37 (0.32 - 43.2)	0.14
Mean age at sample draw (yr) (range)		65.34 (52.28 – 74.12)	63.57 (50.62 – 76.86)	0.26
Mean BMI (kg/m2) (range)		26.28 (17.8 – 38.70)	25.33 (19.74 – 35.14)	0.29
Mean time from sample collection to diagnosis (months) (range)		21.29 (0.99 - 52.01)	24.18 (0.99 – 70.09)	0.40
HRT use (at randomisation)				
	yes	1	6	0.14
	no	31	38	
OCP use (ever)				
	yes	18	18	0.19
	no	14	26	
Diabetes				
	yes	2	1	0.40
	no	30	43	

Supplementary Table 7 Number of UKCTOCS samples per time-group per data set for which AFP, TEK and IGFBP1 were also measured. See Methods section in the main text for details. PDAC stands for pancreatic ductal adenocarcinoma.

Single time-group	Training set number of PDAC samples	Training set number of Control samples	Test set number of PDAC samples	Test set number of Control samples
0-1	33	41	9	9
1-2	30	27	8	17
2-3	26	32	11	8
3-4	20	16	3	7
4+	17	16	1	3

Supplementary Table 8 Subset characteristics within the training set for which LRG1, PIGR, REG3A, F12, AGR2, IL17RA, SERPINA1 and THBS1 were also measured. P values were calculated according to a logistic regression model with a bias reduction method (see Methods), for the whole distribution. This table corresponds to taking all samples from all time-groups, i.e., 0 to 4+. na: not applicable. See Methods section in the main text for details. BMI represents Body Mass Index. HRT stands for Hormone Replacement Therapy. OCP represents Oral Contraceptive Pill use, ever. OR stands for odds ratio.

Variable		Cases	Controls	*P* value
No. samples		62	87	
Tumour site				
	tail	5	na	
	body	10	na	
	body/tail	2	na	
	head	23	na	
	unspecified	22	na	
Mean time to spin (h) (range)		22.04 (0.48 – 46.40)	21.56 (0.82 – 45.82)	0.64
Mean age at sample draw (yr) (range)		63.81 (51.57 – 74.35)	62.75 (50.56 – 76.08)	0.36
Mean BMI (kg/m2) (range)		18.64 (27.44 – 40. 06)	27.43 (17.91 – 44.39)	0.99
Mean time from sample collection to diagnosis (months) (range)		22.59 (1.02 – 66.01)	na	
HRT use (at randomisation)				
	yes	3	17	0.0083 (OR=0.24)
	no	59	70	
OCP use (ever)				
	yes	37	47	0.50
	no	25	15	
Diabetes				
	yes	14	6	0.0064 (OR=3.75)
	no	48	81	

Supplementary Table 9 Subset characteristics within the test set for which LRG1, PIGR, REG3A, F12, AGR2, IL17RA, SERPINA1 and THBS1 were also measured. P values were calculated according to a logistic regression model with a bias reduction method (see Methods), for the whole distribution. This table corresponds to taking all samples from all time-groups, i.e., 0 to 4+. na: not applicable. See Methods in the main text for details. BMI represents Body Mass Index. HRT stands for Hormone Replacement Therapy. OCP represents Oral Contraceptive Pill use, ever. OR stands for odds ratio.

Variable		Cases	Controls	*P* value
No. samples		27	28	
Tumour site				
	tail	1	na	
	body	2	na	
	body/tail	1	na	
	head	11	na	
	unspecified	12	na	
Mean time to spin (h) (range)		20.09 (2 – 25.84)	22.09 (4.64 – 46.24)	0.27
Mean age at sample draw (yr) (range)		66.85 (56.17 – 73.11)	61.81 (50.59 – 74.95)	0.0054 (OR=1.12)
Mean BMI (kg/m2) (range)		26.33 (18.64 – 42.72)	24.35 (19.74 – 29.88)	0.086
Mean time from sample collection to diagnosis (months) (range)		29.09 (0.99 – 66.07)	na	
HRT use (at randomisation)				
	yes	0	6	0.010 (OR=0.063)
	no	27	2	
OCP use (ever)				
	yes	17	18	0.92
	no	10	10	
Diabetes				
	yes	2	0	0.20
	no	25	28	

Supplementary Table 10 Number of UKCTOCS samples per time-group per data set for which LRG1, PIGR, REG3A, F12, AGR2, IL17RA, SERPINA1 and THBS1 were also measured. See Methods section in the main text for details.

Single time-group	Training set number of PC samples	Training set number of Control samples	Test set number of PC samples	Test set number of Control samples
0-1	26	34	8	7
1-2	10	13	6	9
2-3	11	14	2	2
3-4	8	12	4	5
4+	7	14	7	5

Supplementary Table 11 Cancer-associated proteins measured on the Olink Oncology II panel used in this project. The remaining biomarkers were done in-house. Protein names are listed to be consistent with those provided by Olink.

PODXL	VEGF-A	VEGFR-2/KDR	VEGFR-3/LFT4	RSPO3	IL6	IFN-gamma-R1/IFNGR1
CXCL17	MK/MDK	MIC-A/B	WFDC2	ESM-1	MSLN	CEACAM1
ITGAV	GPC1	SYND1/SDC1	PVRL4/NECTIN4	TXLNA	Gal-1/GAL	LYPD3
IGF1R	LYN	ABL1	EPHA2	CDKN1A	PPY	SCF/KITLG
EGF	AREG/AR	ErbB2/HER2	ErbB3/HER3	ErbB4/HER4	WISP-1/CCN4	WIF-1
TRAIL/TNFSF10	TNFSF13	TNFRSF19	TNFRSF6B	TLR3	SMAD5/MAD5	FADD
KLK8/hK8	KLK11/hK11	KLK13	KLK14/hK14	CPE	XPNPEP2	CTSV
TFPI-2	SCAMP3	GZMB	GZMH	CYR61/CCN1	ADAM8	ADAM-TS 15
FCRLB	TCL1A	CD27	CD48	CD70	CD160	CD207
ANXA1	S100A4	S100A11	VIM	CRNN	DLL1	SEZ6L
FOLR3/FOL-gamma	5'-NT/NT5E	LY9	CA9/CAIX	FGF-BP1	ICOSLG	FOLR1/FR-alpha
MIA	CEACAM5/CEA	SPARC	HGF	TGFR-2/TGFRB2	FASLG/FasL	MetAP 2
CXCL13	ITGB5	RET	TGF-alpha/TGFA	TNFRSF4	GPNMB	FUR/FURIN

Supplementary Table 12 Number of UKCTOCS samples per time-group per data set. See also Supplementary Tables 1 and 2. PDAC: pancreatic ductal adenocarcinoma.

Single time-group	Training set number of PDAC samples	Training set number of Control samples	Test set number of PDAC samples	Test set number of Control samples
0-1	48	59	13	13
1-2	39	34	11	23
2-3	33	39	12	10
3-4	24	23	6	10
4+	24	30	8	8

Supplementary Table 13 Optimal hyperparameters per base-learner and joined time-group. #ranked features in input space corresponds to the optimal number of top ranked input features according to a logistic regression model with bias correction. See Methods section in the main text for details on ranking and base-learner selection.

Base-learner		Joined time-group				
		0-1	0-2	0-3	0-4	0-4+
C5.0	trials	86	86	78	93	78
	model	tree	tree	tree	rules	tree
	winnow	FALSE	FALSE	FALSE	FALSE	FALSE
	#ranked features in input space	10	30	30	40	20
NNET	size	2	13	10	8	7
	decay	0.12	3.00	3.18	3.47	6.30
	#ranked features in input space	10	10	10	30	10
SVM	sigma	0.077	0.0078	0.018	0.051	0.011
	C	0.48	0.034	0.42	1.23	0.039
	#ranked features in input space	10	10	10	20	10
GLMNET	alpha	0.0032	0.0032	0.021	0.0032	0.0032
	lambda	0.20	0.20	0.061	0.20	0.20
	#ranked features in input space	50	20	10	30	20
GAUSSPR	sigma	0.061	0.0055	0.0064	0.039	0.0060
	#ranked features in input space	10	20	20	20	20
RRF	mtry	7	13	14	35	15
	coefReg	0.97	0.92	0.98	0.98	0.93
	coefImp	0.00019	0.098	0.059	0.058	0.014
	#ranked features in input space	10	20	40	40	20
XGBOOST	nrounds	451	469	883	834	317
	max_depth	3	4	6	7	9
	eta	0.16	0.14	0.42	0.083	0.014
	gamma	1.93	4.11	0.18	5.06	1.34
	subsample	0.91	0.77	0.88	0.91	0.93
	colsample_bytree	0.65	0.48	0.36	0.45	0.49
	rate-drop	0.14	0.30	0.086	0.20	0.10
	skip_drop	0.50	0.24	0.15	0.73	0.33
	min_child_weight	1	0	1	1	0
	#ranked features in input space	10	30	10	30	20
ADABAG	mfinal	74	62	94	92	98
	maxdepth	3	6	23	27	23
	#ranked features in input space	10	50	40	50	20
NB	fL	0	0	0	0	0
	usekernel	TRUE	TRUE	TRUE	FALSE	TRUE
	adjust	1	1	1	1	1
	#ranked features in input space	10	10	10	10	10
GLMStepAIC	-	-	-	-	-	-
	#ranked features in input space	10	10	10	30	20

Supplementary References:

1 O'Brien, D. P. *et al.* Serum CA19-9 is significantly up-regulated up to 2 years prior to diagnosis with pancreatic cancer: implications for early disease detection. *Clin Cancer Res* **21**, 622-631, doi:doi:10.1158/1078-0432.CCR-14-0365 (2015).

2 Kim, J. *et al.* Detection of early pancreatic ductal adenocarcinoma with thrombospondin-2 and CA19-9 blood markers. *Sci Transl Med* **9**, doi:10.1126/scitranslmed.aah5583 (2017).

3 Ansari, D., Toren, W., Zhou, Q., Hu, D. & Andersson, R. Proteomic and genomic profiling of pancreatic cancer. *Cell Biol Toxicol* **35**, 333-343, doi:10.1007/s10565-019-09465-9 (2019).

4 Pereira, S. P. *et al.* Early detection of pancreatic cancer. *Lancet Gastroenterol Hepatol* **5**, 698-710, doi:10.1016/S2468-1253(19)30416-9 (2020).

5 Root, A., Allen, P., Tempst, P. & Yu, K. Protein Biomarkers for Early Detection of Pancreatic Ductal Adenocarcinoma: Progress and Challenges. *Cancers (Basel)* **10**, doi:10.3390/cancers10030067 (2018).

6 Blei, D. M. & Jordan, M. I. Variational Inference for Dirichlet Process Mixtures. *Bayesian Analysis* **1**, 121-143, doi:Doi 10.1214/06-Ba104 (2006).

7 Blagus, R. & Lusa, L. SMOTE for high-dimensional class-imbalanced data. *BMC Bioinformatics* **14**, 106, doi:10.1186/1471-2105-14-106 (2013).

8 Lunardon, N., Menardi, G. & Torelli, N. ROSE: a Package for Binary Imbalanced Learning. *The R Journal* **6**, 79-89 (2014).
