# Supplementary material for: Serum biomarker-based early detection of pancreatic ductal adenocarcinomas with ensemble learning
Source: Commun Med (Lond). 2023 Jan 20;3:10. doi: 10.1038/s43856-023-00237-5 (PMC9860022; doi:10.1038/s43856-023-00237-5)
Supplement: Supplementary file 10 — Reporting Summary [file 43856_2023_237_MOESM10_ESM.pdf]

Corresponding author(s): Dr Nuno Rocha Nene

Last updated by author(s): 24/11/22

Reporting Summary

Nature Research wishes to improve the reproducibility of the work that we publish. This form provides structure for consistency and transparency in reporting. For further information on Nature Research policies, see our Editorial Policies and the Editorial Policy Checklist.

Statistics

For all statistical analyses, confirm that the following items are present in the figure legend, table legend, main text, or Methods section.

n/a	Confirmed	
☐	☒	The exact sample size (n) for each experimental group/condition, given as a discrete number and unit of measurement
☐	☒	A statement on whether measurements were taken from distinct samples or whether the same sample was measured repeatedly
☐	☒	The statistical test(s) used AND whether they are one- or two-sided<br>*Only common tests should be described solely by name; describe more complex techniques in the Methods section.*
☐	☒	A description of all covariates tested
☐	☒	A description of any assumptions or corrections, such as tests of normality and adjustment for multiple comparisons
☐	☒	A full description of the statistical parameters including central tendency (e.g. means) or other basic estimates (e.g. regression coefficient) AND variation (e.g. standard deviation) or associated estimates of uncertainty (e.g. confidence intervals)
☐	☒	For null hypothesis testing, the test statistic (e.g. F, t, r) with confidence intervals, effect sizes, degrees of freedom and P value noted<br>*Give P values as exact values whenever suitable.*
☐	☒	For Bayesian analysis, information on the choice of priors and Markov chain Monte Carlo settings
☒	☐	For hierarchical and complex designs, identification of the appropriate level for tests and full reporting of outcomes
☐	☒	Estimates of effect sizes (e.g. Cohen's d, Pearson's r), indicating how they were calculated

Our web collection on statistics for biologists contains articles on many of the points above.

Software and code

Policy information about availability of computer code

Data collection	This nested case control discovery study was approved by the Joint UCL/UCLH Research Ethics Committee A (Ref. 05/Q0505/57). Written informed consent was obtained from donors and no data allowing identification of patients was provided. The study set comprised serum from post-menopausal women aged 50-74 recruited to UKCTOCS between 2001 and 2005 and collected according to an SOP. All participants were ‘flagged’ with the national agencies for cancer registrations and deaths using their NHS number. Women subsequently diagnosed with pancreatic ductal adenocarcinoma (cases) were identified by cross-referencing with the Health and Social Care Information Centre cancer registry codes and death codes (ICD10 C25.0/1/2/3/9). Confirmation of diagnosis was sought from GPs and consultants through questionnaire and from the Hospitals Episode Statistics database.<br><br>As an external validation cohort, we resorted to the the Accelerated Diagnosis of neuro Endocrine and Pancreatic TumourS (ADEPTS) study (UCL/UCLH Research Ethics Committee reference 06/Q0512/106), which is an early biomarker study aiming to detect pancreatic cancer in patients at a much earlier stage. The ADEPTS study, previously referred to as TRANSlational research in BILiary tract and pancreatic diseases (TRANSBIL) study, collected serum samples from adult patients who presented to University College London and the Royal Free London Hospitals between 2017-2019 with abdominal symptoms suggestive of hepatobiliary disorders and pancreatic cancer. For the purpose of this work, samples from patients with no underlying gastrointestinal disorders or samples from cases diagnosed with pancreatic cancer were used. 17 PDAC cases and 17 controls were available for the work presented here. The controls from the ADEPTS study are the closest to the control population collected from UKCTOCS as they did not present underlying gastrointestinal pathology. The PDAC cases used here had been matched by age, gender and diabetes status. Hormone replacement therapy (HRT) use at randomization and oral contraceptive pill (OCP) use (ever) information was not collected for the female participants. All patients have given written consent for the use of their samples for research purposes and data were anonymized. The samples were processed according to NIHR standards 28 and diagnoses were confirmed by interrogating patient hospital electronic records at University College London and the Royal Free Hospitals.
Data analysis	- ROC curves were generated with the pROC R package (version 1.15.3).<br>-In order to evaluate the association between each of the single markers, including the clinical covariates, and PDAC status, we resorted to the logistic regression model implemented in the logistf R package (https://cran.r-project.org/web/packages/logistf/logistf.pdf, version 1.23).

-The ensemble models relied on the performance of the base-learners highlighted below (all current versions):
• Decision trees and rule-based models for pattern recognition (C50, https://cran.r-project.org/web/packages/C50/C50.pdf , version 0.1.3.1);
• Support vector machines with radial basis function kernel (SVM, https://cran.r-project.org/web/packages/kernlab/kernlab.pdf, version 0.9.29);
• Regularized random forests (RRF, https://cran.r-project.org/web/packages/RRF/RRF.pdf, version 1.9.1);
• Neural networks with feature extraction (NNET, https://cran.r-project.org/web/packages/nnet/nnet.pdf, version 7.3.14);
• Gaussian process with radial basis function kernel (GAUSSPR, https://cran.r-project.org/web/packages/kernlab/kernlab.pdf, version 0.9.29)
• Lasso and elastic-net regularized generalized linear models (GLMNET, https://cran.r-project.org/web/packages/glmnet/glmnet.pdf, version 4.0.2)
• Bagged Adaptive Boosting (ADABAG, https://cran.r-project.org/web/packages/adabag/adabag.pdf, version 4.2)
• Extreme gradient boosting (XGBOOST, https://cran.r-project.org/web/packages/xgboost/xgboost.pdf, version 1.2.0.1)
• Generalized Linear Model with Stepwise Feature Selection with Akaike Information criterion (GLMStepAIC, https://cran.r-project.org/web/packages/MASS/MASS.pdf, version 7.3.53)
• Naïve Bayes classifier (NB, https://cran.r-project.org/web/packages/klaR/klaR.pdf, version 0.6.15)
- Bayesian Model Averaging (https://cran.r-project.org/web/packages/BMA/BMA.pdf, version 3.18.14) with an underlying logistic regression model
- The variable importance routine selected for evaluating feature importance in each base-learner was a model-agnostic method based on a simple feature importance ranking measure, implemented in the R package vip (https://cran.r-project.org/web/packages/vip/index.html, version 0.3.2).
-The enrichment analysis for each of the signatures developed with single and joined time-groups was performed with the gprofiler2 R package (https://cran.r-project.org/web/packages/gprofiler2/gprofiler2.pdf, version 0.2.0).

For manuscripts utilizing custom algorithms or software that are central to the research but not yet described in published literature, software must be made available to editors and reviewers. We strongly encourage code deposition in a community repository (e.g. GitHub). See the Nature Research guidelines for submitting code & software for further information.

Data

Policy information about availability of data

All manuscripts must include a data availability statement. This statement should provide the following information, where applicable:

- Accession codes, unique identifiers, or web links for publicly available datasets
- A list of figures that have associated raw data
- A description of any restrictions on data availability

Data requestors will need to sign a data access agreement and in keeping with patient consent for secondary use, obtain ethical approval for any new analyses. All packages used in the pre-processing of data and subsequent analysis have been identified to secure full reproducibility.

Field-specific reporting

Please select the one below that is the best fit for your research. If you are not sure, read the appropriate sections before making your selection.

☒ Life sciences ☐ Behavioural & social sciences ☐ Ecological, evolutionary & environmental sciences

For a reference copy of the document with all sections, see nature.com/documents/nr-reporting-summary-flat.pdf

Life sciences study design

All studies must disclose on these points even when the disclosure is negative.

Sample size	In total, for UKCTOCS, 143 cases were identified (with 219 associated serum samples) that had not been registered as having any other cancer since randomization and that had a confirmed diagnosis of pancreatic cancer. Matched non-cancer controls, i.e., with no cancer registry code, from individual women were selected based on collection date and centre to minimize variation due to handling and storage. From this set, 248 controls were selected. 35 of the PDAC cases had longitudinal data, with between 2 and 6 annual longitudinal samples per individual years before diagnosis. Due to the design of the UKCTOCS study, PDAC stage for all the cases at the time of diagnosis was not available.<br><br>For the external validation set, a subset collected from ADEPTS,17 PDAC cases and 17 controls which did have any underlying gastrointestinal disorders were available. The controls from the ADEPTS study are the closest to the control population collected from UKCTOCS as they did not present underlying gastrointestinal pathology.
Data exclusions	No data was excluded
Replication	All attempts at replication were successful
Randomization	We divided the whole set of samples into a UKCTOCS training (2/3) and test (1/3) set, by stratifying for age quartile, HRT use at randomization, OCP use (ever), Diabetes status, BMI quartile, PDAC and control status and sample single time-group, i.e., 0-1,1-2,2-3,3-4 and 4+ YTD, attributed to each sample determined by the time from collection to diagnosis. The PDAC signature developed in the UKCTOCS training set was also applied to the ADEPTS subset of samples.
Blinding	Allocations were done according standard procedures in machine learning. Model development was done in the training set and the reported performances in the test set and external validation set are completely unbiased.

Reporting for specific materials, systems and methods

We require information from authors about some types of materials, experimental systems and methods used in many studies. Here, indicate whether each material, system or method listed is relevant to your study. If you are not sure if a list item applies to your research, read the appropriate section before selecting a response.

Materials & experimental systems

n/a	Involved in the study
☒	☐ Antibodies
☒	☐ Eukaryotic cell lines
☒	☐ Palaeontology and archaeology
☒	☐ Animals and other organisms
☐	☒ Human research participants
☐	☒ Clinical data
☒	☐ Dual use research of concern

Methods

n/a	Involved in the study
☒	☐ ChIP-seq
☒	☐ Flow cytometry
☒	☐ MRI-based neuroimaging

Human research participants

Policy information about studies involving human research participants

Population characteristics

Our main dataset is part of a nested case control study within UK Collaborative Trial of Ovarian Cancer Screening (UKCTOCS) (Menon, U. et al. Lancet Oncol 10, 327-340 (2009), Menon, U. et al. Bmj 337, a2079 (2008) and is comprised of 143 individuals with PDAC and 248 controls. 35 of the PDAC-diagnosed patients provided longitudinal samples, with between 2 and 6 annual samples per individual collected prior to diagnosis. The study set comprised serum from post-menopausal women aged 50-74 recruited to UKCTOCS between 2001 and 2005 and collected according to an SOP.

Time intervals between sample collection and serum/plasma isolation, i.e., time to spin, were comparable between PDAC cases and controls. There was no significant difference in the mean time to spin between cases and controls for the whole study data set (P=0.93), with the ranges being also very similar. The distribution of ages at sample draw showed a significant association (OR=1.06, P < 0.0001) with PDAC status, with cases having a mean value at 64.94 years and controls at 62.48 years. As a single variate, subject BMI was not a significant predictor in the entire data set (P=0.078). HRT, on the other hand, was (OR = 0.41, P=0.0010). This was also the case for OCP (OR = 1.47, P=0.039). Overall, diabetes was the strongest predictor of risk for PDAC among the clinical covariates (OR = 4.99, P<0.0001).

As an external validation cohort, we resorted to the ADEPTS study (IRAS Number: 234637, NIHR Portfolio no. 7343) which is an early biomarker study aiming to detect pancreatic cancer in patients at a much earlier stage. This initiative developed a prospective biobank and an early diagnostic tool that can differentiate early, pancreatic neuroendocrine tumours and high-risk pancreatic lesions from benign disease. The number of cases and controls selected for external validation of the PDAC signature developed in the UKCTOCS samples were the following: 17 PDAC cases and 17 controls which did have any underlying gastrointestinal disorders were available. The controls from the ADEPTS study are the closest to the control population collected from UKCTOCS as they did not present underlying gastrointestinal pathology. The PDAC cases used here had been matched by age, gender and diabetes. HRT and OCP use were not collected for any of the women in the study. Yet, clinical covariates such as Diabetes, Age, Gender and BMI were available. Apart from Age (OR= 1.08 (95% CI 1.03-1.16), P=0.0054), no other had an association with PDAC, but the dataset available for analysis here was relatively small.

Recruitment

The UKCTOCS nested case control discovery study was approved by the Joint UCL/UCLH Research Ethics Committee A (Ref. 05/Q0505/57). Written informed consent was obtained from donors and no data allowing identification of patients was provided. The study set comprised serum from post-menopausal women aged 50-74 recruited to UKCTOCS between 2001 and 2005 and collected according to an SOP 24,25. All participants were 'flagged' with the national agencies for cancer registrations and deaths using their NHS number. Women subsequently diagnosed with pancreatic ductal adenocarcinoma (cases) were identified by cross-referencing with the Health and Social Care Information Centre cancer registry codes and death codes (ICD10 C25.0/1/2/3/9). Confirmation of diagnosis was sought from GPs and consultants through questionnaire and from the Hospitals Episode Statistics database. In total, 143 cases were identified (with 219 associated serum samples) that had not been registered as having any other cancer since randomization and that had a confirmed diagnosis of pancreatic cancer. Matched non-cancer controls, i.e., with no cancer registry code, from individual women were selected based on collection date and centre to minimize variation due to handling and storage. From this set, 248 controls were selected. 35 of the PDAC cases had longitudinal data, with between 2 and 6 annual longitudinal samples per individual years before diagnosis. Due to the design of the UKCTOCS study, PDAC stage for all the cases at the time of diagnosis was not available.

The ADEPTS study (previously known as the TRANSBIL study) collected serum samples from adult patients who presented to University College London and the Royal Free London Hospitals between 2017-2019 with abdominal symptoms suggestive of hepatobiliary disorders and pancreatic cancer. For the purpose of this work, samples from patients with no underlying gastrointestinal disorders or samples from cases diagnosed with pancreatic cancer were used. All patients have given written consent for the use of their samples for research purposes and data were anonymized. The samples were processed according to NIHR standards (Tuck et l.J Proteome Res. 2009 Jan; 8(1): 113–117) and diagnoses were confirmed by interrogating patient hospital electronic records at University College London and the Royal Free Hospitals.

Ethics oversight	UKCTOCS: Joint UCL/UCLH Research Ethics Committee A (Ref. 05/Q0505/57)<br>ADEPTS: UCL/UCLH Research Ethics Committee (Ref. 06/Q0512/106).

Note that full information on the approval of the study protocol must also be provided in the manuscript.

Clinical data

Policy information about clinical studies

All manuscripts should comply with the ICMJE guidelines for publication of clinical research and a completed CONSORT checklist must be included with all submissions.

Clinical trial registration	NCT00058032
Study protocol	The current paper uses data from UKCTOCS, but was not designed to evaluate the same things. The PDAC cases and controls were selected according to what is outlined above. It also uses cases and controls from the ADEPTS study (see details above)
Data collection	For data collection see above
Outcomes	Outcomes were not defined as the paper focuses on data that had been collected for UKCTOCS, which addresses early detection of ovarian cancer.
